# Clinical, microbiologic, and immunologic determinants of mortality in hospitalized patients with HIV-associated tuberculosis: A prospective cohort study

**DOI:** 10.1371/journal.pmed.1002840

**Published:** 2019-07-05

**Authors:** Charlotte Schutz, David Barr, Bruno B. Andrade, Muki Shey, Amy Ward, Saskia Janssen, Rosie Burton, Katalin A. Wilkinson, Bianca Sossen, Kiyoshi F. Fukutani, Mark Nicol, Gary Maartens, Robert J. Wilkinson, Graeme Meintjes

**Affiliations:** 1 Wellcome Centre for Infectious Diseases Research in Africa (CIDRI-Africa), Institute of Infectious Disease and Molecular Medicine, University of Cape Town, Cape Town, South Africa; 2 Department of Medicine, University of Cape Town, Cape Town, South Africa; 3 Wellcome Trust Liverpool Glasgow Centre for Global Health Research, University of Liverpool, Liverpool, United Kingdom; 4 Instituto Gonçalo Moniz, Fundação Oswaldo Cruz, Salvador, Brazil; 5 Multinational Organization Network Sponsoring Translational and Epidemiological Research (MONSTER) Initiative, Salvador, Brazil; 6 Division of Infectious Diseases, Department of Medicine, Vanderbilt University School of Medicine, Nashville, Tennessee, United States of America; 7 Universidade Salvador (UNIFACS), Laureate Universities, Salvador, Brazil; 8 Amsterdam University Medical Center, University of Amsterdam, Amsterdam, the Netherlands; 9 Khayelitsha Hospital, Department of Medicine, Cape Town, South Africa; 10 The Francis Crick Institute, London, United Kingdom; 11 Faculdade de Tecnologia e Ciências (FTC), Salvador, Brazil; 12 Division of Medical Microbiology, University of Cape Town and National Health Laboratory Services, Cape Town, South Africa; 13 Division of Clinical Pharmacology, Department of Medicine, University of Cape Town, Cape Town, South Africa; 14 Department of Medicine, Imperial College, London, United Kingdom; University of Cape Town, SOUTH AFRICA

## Abstract

**Background:**

In high-burden settings, case fatality rates are reported to be between 11% and 32% in hospitalized patients with HIV-associated tuberculosis, yet the underlying causes of mortality remain poorly characterized. Understanding causes of mortality could inform the development of novel management strategies to improve survival. We aimed to assess clinical and microbiologic determinants of mortality and to characterize the pathophysiological processes underlying death by evaluating host soluble inflammatory mediators and determined the relationship between these mediators and death as well as biomarkers of disseminated tuberculosis.

**Methods and findings:**

Adult patients with HIV hospitalized with a new diagnosis of HIV-associated tuberculosis were enrolled in Cape Town between 2014 and 2016. Detailed tuberculosis diagnostic testing was performed. Biomarkers of tuberculosis dissemination and host soluble inflammatory mediators at baseline were assessed. Of 682 enrolled participants, 576 with tuberculosis (487/576, 84.5% microbiologically confirmed) were included in analyses. The median age was 37 years (IQR = 31–43), 51.2% were female, and the patients had advanced HIV with a median cluster of differentiation 4 (CD4) count of 58 cells/L (IQR = 21–120) and a median HIV viral load of 5.1 log_10_ copies/mL (IQR = 3.3–5.7). Antituberculosis therapy was initiated in 566/576 (98.3%) and 487/576 (84.5%) started therapy within 48 hours of enrolment. Twelve-week mortality was 124/576 (21.5%), with 46/124 (37.1%) deaths occurring within 7 days of enrolment. Clinical and microbiologic determinants of mortality included disseminated tuberculosis (positive urine lipoarabinomannan [LAM], urine Xpert MTB/RIF, or tuberculosis blood culture in 79.6% of deaths versus 60.7% of survivors, *p* = 0.001), sepsis syndrome (high lactate in 50.8% of deaths versus 28.9% of survivors, *p* < 0.001), and rifampicin-resistant tuberculosis (16.9% of deaths versus 7.2% of survivors, *p* = 0.002). Using non-supervised two-way hierarchical cluster and principal components analyses, we describe an immune profile dominated by mediators of the innate immune system and chemotactic signaling (interleukin-1 receptor antagonist [IL-1Ra], IL-6, IL-8, macrophage inflammatory protein-1 beta [MIP-1β]/C-C motif chemokine ligand 4 [CCL4], interferon gamma-induced protein-10 [IP-10]/C-X-C motif chemokine ligand 10 [CXCL10], MIP-1 alpha [MIP-1α]/CCL3), which segregated participants who died from those who survived. This immune profile was associated with mortality in a Cox proportional hazards model (adjusted hazard ratio [aHR] = 2.2, 95%CI = 1.9–2.7, *p* < 0.001) and with detection of biomarkers of disseminated tuberculosis. Clinicians attributing causes of death identified tuberculosis as a cause or one of the major causes of death in 89.5% of cases. We did not perform longitudinal sampling and did not have autopsy-confirmed causes of death.

**Conclusions:**

In this study, we did not identify a major contribution from coinfections to these deaths. Disseminated tuberculosis, sepsis syndrome, and rifampicin resistance were associated with mortality. An immune profile dominated by mediators of the innate immune system and chemotactic signaling was associated with both tuberculosis dissemination and mortality. These findings provide pathophysiologic insights into underlying causes of mortality and could be used to inform the development of novel treatment strategies and to develop methods to risk stratify patients to appropriately target novel interventions. Causal relationships cannot be established from this study.

## Abstract 2

In a prospective cohort study, Charlotte Schutz and colleagues identify biomarkers and clinical characteristics associated with tuberculosis dissemination and death in patients with HIV and TB.

## Author summary

**Why was this study done?:**

- Patients with HIV who are hospitalized with new tuberculosis are at high risk of death even after starting on standard antituberculosis treatment, and the reasons for this are not well understood.
- To facilitate the development of novel treatment strategies that improve survival, a better understanding of the cause of these deaths is required.

**What did the researchers do and find?:**

- We enrolled 576 patients with HIV hospitalized with a new diagnosis of tuberculosis and followed them up for 12 weeks.
- We systematically performed diagnostic tests for tuberculosis in blood and urine as a measure of tuberculosis dissemination (the spread of tuberculosis through the body via the bloodstream). We characterized the immune response in blood of these patients and compared these measures in patients who died with those who survived.
- In our study, 22% of patients died within 12 weeks, and those who had a greater number of positive measures for tuberculosis dissemination were more likely to die.
- An immune profile characterized by increased markers of the innate immune response and proteins that attract innate cells to tissue was associated with mortality, and this profile was also associated with more disseminated tuberculosis.

**What do these findings mean?:**

- Our findings provide novel insights into the immune response associated with death in patients with HIV who are severely ill with tuberculosis.
- This knowledge can be used to design, evaluate, and monitor new treatment strategies, including immune-based therapies.
- Our study evaluated immune responses at a single time point prior to treatment, and future studies should evaluate these responses longitudinally on antituberculosis treatment.

## Introduction

HIV-associated tuberculosis comprises 10% of global tuberculosis cases but contributes a disproportionate 22% of tuberculosis deaths [1]. Despite advances in diagnostics and widespread availability of treatment, tuberculosis remains the leading cause of death (40%), hospitalization (18%) and in-hospital death (25%) in patients with HIV worldwide [1–3]. In high-burden settings, hospitalized patients with HIV-associated tuberculosis have case fatality rates between 11% and 32% [4–10]. The underlying causes of mortality remain poorly characterized. In outpatient cohorts with HIV-associated tuberculosis, early mortality has been associated with high baseline immune activation [11,12], persistent or increased immune activation on antiretroviral therapy (ART) [12], and failure to recover cellular immune responses to *Mycobacterium tuberculosis* [13]. In Africa, disseminated tuberculosis is found in 88% of autopsies of patients dying with HIV-associated tuberculosis [14]. *M*. *tuberculosis* bloodstream infection (MTB BSI or mycobacteremia) is the most common microbiologic diagnosis in patients with HIV admitted with a clinical sepsis syndrome in Africa [15,16] and is associated with higher mortality [16–19]. Patients with MTB BSI may present with sepsis syndrome [19,20] and with septic shock, which has high mortality, especially if antituberculosis treatment is delayed [21].

Patients admitted with HIV-associated tuberculosis are frequently acutely ill, with inpatient deaths occurring at a median of 4–5 days after admission in autopsy series [22–24], despite many receiving appropriate antituberculosis therapy [22]. Disseminated tuberculosis is challenging to diagnose, and while improved diagnostic tests like urine lipoarabinomannan (LAM) and urine Xpert MTB/RIF assays facilitate more rapid detection of disseminated tuberculosis [18], mortality in inpatients with suspected HIV-associated tuberculosis remains high despite implementation of these rapid tests [25,26].

Autopsy series also report frequent additional opportunistic infections, which may contribute to death [23,24,27]. However, autopsy studies do not characterize the dynamic pathophysiological processes underlying mortality.

There is an urgent need for improved, evidence-based, acute management strategies to increase survival in hospitalized patients with HIV-associated tuberculosis. An improved understanding of pathophysiological processes and contributors to mortality could facilitate the discovery of novel therapeutic targets and strategies, enable appropriate risk stratification to target new interventions appropriately, and aid monitoring of treatment responses.

In this prospective observational study, we followed a large cohort of participants with HIV who were diagnosed with active tuberculosis while admitted to hospital. We measured markers of tuberculosis dissemination and host soluble inflammatory mediators. Our first objective was to determine the 12-week case fatality rate and the contribution of antituberculosis drug resistance, dissemination of tuberculosis, and coinfections to these deaths. Our second objective was to characterize the pathophysiological processes underlying these deaths by specifically defining the relationship between biomarkers of tuberculosis dissemination and death, the relationship between host inflammatory mediators and death, and the relationship between biomarkers of tuberculosis dissemination and host inflammatory mediators.

## Methods

### Study design and setting

Patients were enrolled into a prospective observational cohort study at Khayelitsha Hospital, Cape Town, from January 2013 until October 2016. This 240-bed hospital has 60 medical beds and an emergency room. The antenatal HIV seroprevalence in Khayelitsha was 34% in 2015 [28] and the tuberculosis notification rate was 917/100,000 in 2015, with 60% of cases being HIV-coinfected (Judy Caldwell, City of Cape Town Department of Health, 16 May 2016). ART and antituberculosis therapy (including treatment for drug-resistant tuberculosis) are accessible at government clinics free of charge. Patients are referred from surrounding primary health clinics when they require inpatient care. Primary health clinics have the capacity to initiate intravenous fluids and intravenous antibiotic treatment while awaiting patient transfer to hospital. This study was conducted using a prospective protocol that included an analysis plan (S1 Document) and is reported according to the STROBE statement (S1 STROBE checklist).

### Participants

All patients in the emergency room and medical wards were screened on weekdays. Adults with HIV with a cluster of differentiation 4 (CD4) count <350 cells/μL and a high clinical suspicion of new tuberculosis (i.e., tuberculosis was considered the number one differential diagnosis on referral or after review by hospital or study staff) were eligible for enrolment. Pregnant patients, patients who received antituberculosis therapy within the past month, or patients who were recently initiated and received three or more doses of antituberculosis therapy were not eligible for enrolment. A list of all potentially eligible patients was compiled daily, and a random selection procedure was followed to enroll 2–4 patients daily. Clinical details, chest X-ray, sputum (spontaneous or induced if unable to produce sputum spontaneously), urine, and blood samples were obtained at enrolment. Participants remained in routine clinical care, and study results were made available to clinical teams. Treatment decisions were made by clinical teams, not study staff. Participants were assessed daily in the ward and after discharge were managed in primary care according to local guidelines. They had a telephonic follow-up at week 4 and returned for a clinical assessment at week 12.

### Laboratory assays

Tuberculosis tests were performed at the National Health Laboratory Services (NHLS). Sputum (if obtained) was sent for tuberculosis culture and Xpert MTB/RIF assay (Cepheid). Urine Xpert MTB/RIF assay was performed on sediment from centrifuged urine as previously described [29]. Mycobacterial blood culture was performed by culturing 5 mL of whole blood in Myco/F Lytic (Becton Dickinson Biosciences) bottles for 42 days. The GenoType MTBDR*plus* assay (Hain Lifesciences) was used to identify *M*. *tuberculosis* complex from positive sputum and blood cultures and provided rifampicin and isoniazid resistance testing. Rifampicin-resistant isolates had susceptibility testing to second-line drugs performed at a local referral laboratory. Urine LAM testing was performed retrospectively on frozen urine samples using the Alere Determine TB LAM antigen test. Strips were read by two independent readers both blind to clinical details. CD4 count, HIV viral load, full blood count, differential count, renal function, liver function, C-reactive protein, procalcitonin, venous lactate, and cytomegalovirus (CMV) viral load tests were performed on all participants by the NHLS. CMV viral load >49 IU/mL on the Argene CMV R-gene platform was regarded as detectable. Serum cryptococcal antigen (CRAG) test was performed by study staff.

Bacterial blood cultures were performed by the study team if the patient had not received intravenous antibiotics at the time of enrolment. Results from all bacterial cultures (blood and other specimens) that were performed by the hospital staff were captured. Hospital staff sent additional samples for tuberculosis testing when required, and this included extrapulmonary samples such as pleural fluid, cerebrospinal fluid, and lymph node aspirates.

Plasma was stored at −80°C for immunology assays. Soluble inflammatory mediators were tested in a randomly selected subset of participants (*n* = 507) on stored plasma at a 1:2 dilution using the Bio-Plex Pro Human Cytokine Standard 27-Plex kit (Group I) on the Biorad Bioplex 200 Luminex platform. The following analytes were measured: interleukin (IL)-1β, IL-1 receptor antagonist (IL-1Ra), IL-2, IL-4, IL-5, IL-6, IL-7, IL-8, IL-9, IL-10, IL-12p70, IL-13, IL-15, IL-17A, eotaxin, basic fibroblast growth factor (FGF), granulocyte colony stimulating factor (G-CSF)/colony stimulating factor 3 (CSF3), granulocyte-macrophage colony stimulating factor (GM-CSF/CSF2), interferon gamma (IFN-γ), interferon gamma-induced protein (IP-10)/ C-X-C motif chemokine ligand 10 (CXCL10), monocyte chemoattractant protein-1 (MCP-1)/C-C motif chemokine ligand 2 (CCL2), macrophage inflammatory protein-1 alpha (MIP-1α/CCL3), MIP-1 beta (MIP-1β/CCL4), platelet-derived growth factor-BB (PDGF), regulated on activation, normal T cell expressed and secreted (RANTES/CCL5), tumor necrosis factor-alpha (TNF-α), and vascular endothelial growth factor (VEGF). ELISA testing (R&D Systems Quantikine) of undiluted stored plasma was used to determine transforming growth factor beta 1 (TGF-β1) concentrations.

### Data collection and definitions

Clinical data were obtained from the patient, hospital folder, and clinical review at enrolment and captured on standard case record forms. Results of all study investigations and additional tuberculosis tests (if performed in-service) were captured. The primary outcome was vital status at week 12. If participants could not be contacted at week 12, extensive searches of regional electronic clinical, pharmacy, and laboratory records were conducted to ascertain vital status. Participants with an entry indicating a clinic visit, collection of medication, or a laboratory test performed beyond week 12 were assumed to be alive at week 12. Based on results of all tuberculosis tests, all participants were classified into mutually exclusive diagnostic groups. Urine LAM more than or equal to grade 1 by two independent readers was regarded as positive. “Microbiologically confirmed tuberculosis” was defined as participants with *M*. *tuberculosis* on at least 1 culture or Xpert MTB/RIF test from any clinical sample. “Probable tuberculosis” was defined as participants without microbiologically confirmed tuberculosis who had positive urine LAM or had a compatible clinical and radiological picture and were treated for tuberculosis and without alternative primary diagnosis made during enrolment admission (S1 Table). “Possible tuberculosis” was defined as participants treated for tuberculosis and another opportunistic infection simultaneously, with indistinguishable clinical and radiological picture with neither infection proven (S2 Table). “No tuberculosis” was defined as participants who had proven alternative diagnosis during admission and not treated for tuberculosis. Possible tuberculosis and no tuberculosis were excluded from analyses.

MTB BSI was defined as having ≥one positive mycobacterial blood culture identified as *M*. *tuberculosis* during enrolment admission. Rifampicin-resistant tuberculosis was defined as rifampicin resistance on any clinical sample using either of the genotypic tests performed at the NHLS (Xpert MTB/RIF or GenoType MTBDRplus).

We quantified the degree of mycobacterial dissemination with a three-point dissemination score, previously described [18]. Participants were allocated one point for each of the following: urine Xpert MTB/RIF assay positive for *M*. *tuberculosis*, urine LAM test positive, mycobacterial blood culture positive and identified as *M*. *tuberculosis*, yielding a score ranging from 0 to 3. Participants who had all three tests performed, valid results for the tests, and known outcome at week 12 were allocated a score (*n* = 457).

Presentation to hospital was defined as the time the patient was triaged in the emergency room. Early deaths were deaths that occurred within 7 days of enrolment, and late deaths are all deaths that occurred after 7 days and within 12 weeks of enrolment.

### Statistical analysis

Sample size calculations were performed for the major analyses in the original protocol (S1 Document). The planned sample size was 660. Our overarching immunology hypothesis was that patients who died would have evidence of a compensatory anti-inflammatory immune response. We changed the original analysis plan in two ways. Firstly, we expanded the repertoire of inflammatory mediators tested (from 12 to 27). Secondly, we added analyses to characterize immune profiles associated with markers of tuberculosis dissemination and mortality with hierarchical cluster analysis, principal components analysis, Cox proportional hazards analysis, and correlation analysis. The median values with IQRs were used as measures of central tendency. Categorical variables are presented using counts with percentage and compared using the Fisher exact or Pearson chi-squared test. Continuous variables were compared between the study groups using the Mann-Whitney *U* test (two-group comparisons) or the Kruskal-Wallis test with the Dunn multiple comparisons ad hoc test or nonparametric linear trend analysis (between three or more groups). Cox proportional hazards models were censored at 28 days to meet the proportional hazards assumption because there was a higher risk of death during the first 28 days after enrolment. Variables were first evaluated separately in unadjusted models and then adjusted for a priori selected variables. Patients lost to follow-up were censored on their last day of contact with health services.

Fluorescence intensity values of soluble inflammatory mediators were used in all the multidimensional analyses, including principal components analysis and hierarchical clustering analyses. Using fluorescence values allows for the analysis of analytes of low abundance and does not require censoring or correction for background, and calculated concentrations are dependent on the distribution of the fluorescence values [30,31].

Inflammatory mediator values were log transformed, z-score normalized, and corrected for multiple comparisons (Holm-Bonferroni method), when appropriate. Correlations were examined using the Spearman rank test. Non-supervised hierarchical cluster analyses were performed using the Ward method and 100× bootstrap. Dendograms represent Euclidean distance. Principal components analysis (with maximum rotation) was performed using log transformed, scaled values of inflammatory mediators.

At the request of a reviewer, we performed a sensitivity analysis, excluding deaths occurring within 7 days of enrolment to exclude deaths that could potentially be attributable to missed bacterial infections at presentation. For this, we repeated the principal components analysis and the Cox proportional hazards analysis, which included the principal components values as variables.

Data were analyzed using R Statistical software, GraphPad Prism 7.0, JMP 14 (SAS), and Gephi 0.9.1.

### Ethical approval

The study was approved by the University of Cape Town Human Research Ethics Committee (UCT HREC), reference number 057/2013. Participants provided written informed consent when possible. Eligible patients with a decreased level of consciousness were enrolled and followed up daily until they regained capacity to participate in the informed consent process, and if not agreeable to participate, were withdrawn from the study. The UCT HREC approved the use of information from participants who died prior to providing informed consent or could not provide consent by the end of study follow-up. This was consistent with the approved protocol. By the end of study follow-up, 47 patients did not provide written informed consent. Of these, 24/47 (51%) died prior to providing written informed consent. Survivors who did not provide written informed consent were participants who did not regain capacity to consent by the time of discharge and either did not return for a study follow-up in person or did not regain capacity to consent by the end of study follow-up.

## Results

### Participant characteristics and mortality

**Fig 1:**
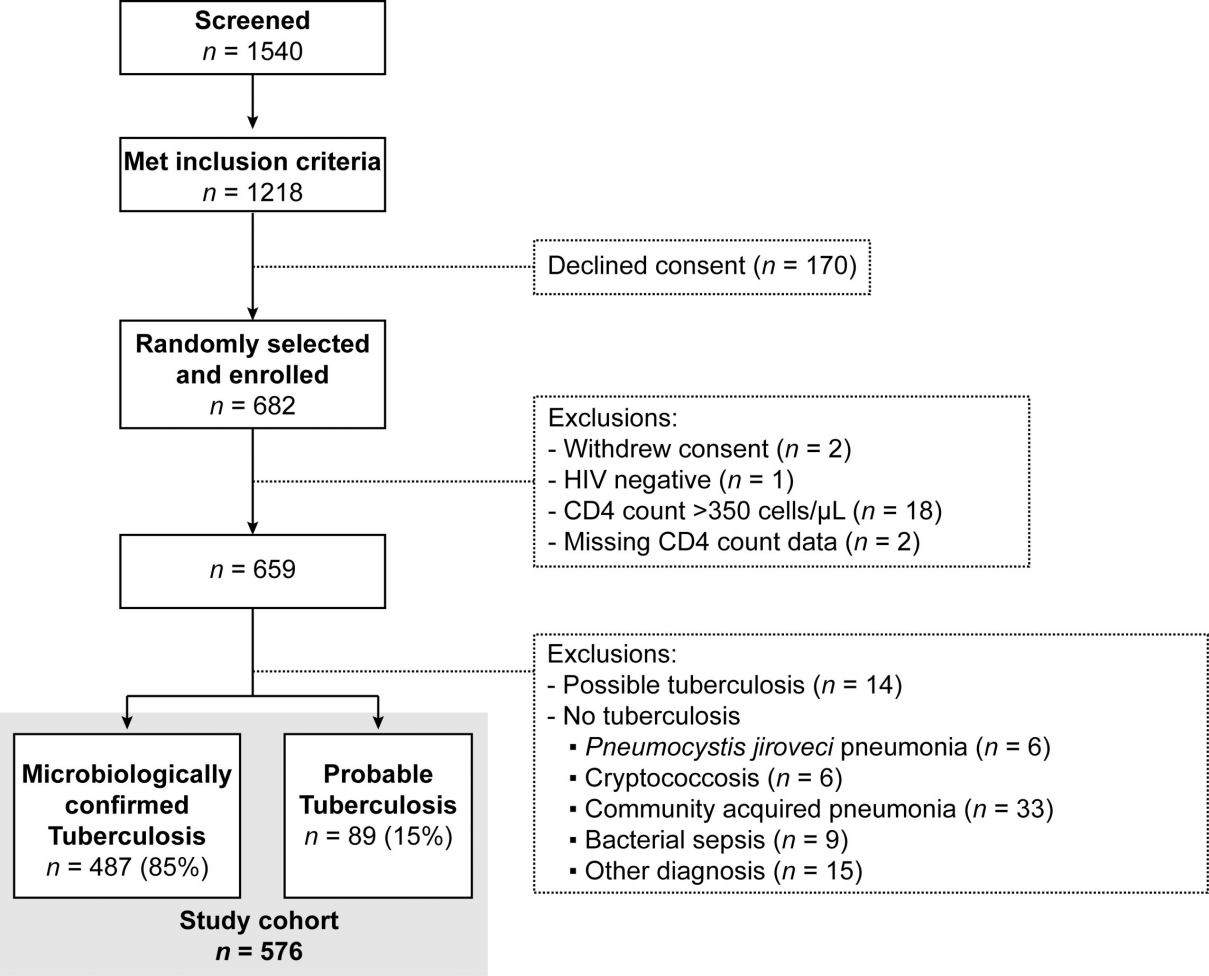
Study flowchart. Showing disposition of screened patients. Patients in the emergency room and medical wards were screened daily on weekdays, and potentially eligible patients were identified. Potentially eligible patients were randomly selected and approached for enrolment in the study. A total of 682 patients were enrolled, and 659 had HIV infection with a CD4 count <350 cells/μL. Tuberculosis was diagnosed in 576 participants (microbiologically confirmed and probable tuberculosis), and these participants are included in the analysis. CD4, cluster of differentiation 4.

A total of 682 hospitalized patients with HIV and with suspected tuberculosis were enrolled a median of 2 days (IQR = 1–3 days, range = 0–10 days) after presentation to hospital. In this analysis, we include tuberculosis cases (*n* = 576), 487/576 (84.5%) of whom were microbiologically confirmed and 89/576 (15.5%) with probable tuberculosis (Fig 1), and we excluded possible and no tuberculosis cases. Twelve-week mortality was 124/576 (21.5%). Death occurred after a median of 12.5 days (IQR = 4–35 days) after enrolment, and 46/124 (37.1%) of deaths occurred within 7 days of enrolment. Nine participants (1.6%) were lost to follow-up.

### Causes of death

Causes of death were clinician attributed, and in many participants it was not possible to attribute the cause of death to a single disease process. Tuberculosis was implicated as the sole underlying cause of death in 55/124 (44.4%) of cases but was implicated as a major cause of death (in combination with other factors, mostly suspected sepsis) in an additional 56/124 (45.2%) of deaths (Table 1). There were 12/124 (9.7%) deaths that were not attributed to tuberculosis and one with unknown cause of death. Rifampicin-resistant tuberculosis was present in 21/124 (16.9%) of deaths (Table 2) and tuberculosis was the sole or major cause of death in 19/21 (90.5%) of rifampicin-resistant participant deaths, or 19/124 (15.3%) of all deaths.

**Table 1:** Clinician-attributed causes of death in participants with HIV-associated tuberculosis.

Clinician-attributed cause of death	*n*
Direct consequence of tuberculosis, including • Central nervous system tuberculosis (*n* = 11) • TB-IRIS (*n* = 2)	55
Tuberculosis and/or bacterial sepsis	32
Tuberculosis and/or bacterial sepsis plus another condition, including • Venous thromboembolism (*n* = 2) • Drug toxicity (*n* = 4) • Renal failure (*n* = 4) • TB-IRIS (*n* = 2)	12
Tuberculosis plus condition other than bacterial sepsis, including • Drug toxicity (*n* = 2) • Cardiomyopathy (*n* = 1) • Venous thromboembolism (*n* = 1) • Cryptococcosis (*n* = 1) • *Pneumocystis jirovecii* pneumonia (*n* = 2) • Renal failure (*n* = 3)	12
Bacterial sepsis	3
Other causes, including • Venous thromboembolism (*n* = 4) • Cardiomyopathy (*n* = 1) • Chronic obstructive pulmonary disease (*n* = 1) • Drug toxicity (*n* = 1) • Carcinoma (*n* = 1) • HIV (*n* = 1)	9
Unknown	1

Abbreviation: TB-IRIS, tuberculosis immune reconstitution inflammatory syndrome.

**Table 2:** Baseline characteristics of hospitalized participants with HIV-associated tuberculosis.

Characteristics		Died	Survived	*p*
		*n* = 124	*n* = 443	
Sex	Female	69 (55.6)	223 (50.3)	0.311
Age, years		39 [33–47]	35 [31–42]	**<0.001**
ART	Previously on ART	35 (28.9)	100 (22.6)	0.308
	Naive	42 (34.7)	179 (40.5)	
	Current ART	44 (36.4)	163 (36.9)	
Tuberculosis history	First episode	64 (53.8)	240 (56.3)	0.676
	Previous TB	55 (46.2)	186 (43.7)	
Ceftriaxone received during admission		108 (87.1)	391 (88.3)	0.755
^1^Antituberculosis therapy category at treatment initiation	Standard first-line regimen	97 (85.1)	421 (95.5)	**<0.001**
	Drug-resistant regimen	8 (7.0)	11 (2.5)	
Weight, kilograms		54 [44–61]	54 [48–63]	0.290
Glasgow Coma Scale <15		27 (21.8)	49 (11.1)	**<0.001**
CD4 count, cells/μL		40 [15–99]	63 [23–134]	**0.002**
HIV viral load, log_10_ copies/mL		5.0 [3.3–5.7]	5.1 [3.3–5.7]	0.655
Hemoglobin, g/dL		8.0 [6.7–10.0]	8.8 [7.4–10.5]	**0.005**
White cell count, ×10^9^/L		7.14 [4.18–10.87]	6.99 [4.51–10.29]	0.826
Absolute lymphocyte count, ×10^9^/L		0.41 [0.26–0.64]	0.69 [0.36–1.06]	**<0.001**
Absolute monocyte count, ×10^9^/L		0.23 [0.12–0.41]	0.36 [0.17–0.61]	**<0.001**
Absolute neutrophil count, ×10^9^/L		5.75 [3.29–8.96]	5.53 [3.20–8.59]	0.683
Platelet count, ×10^9^/L		232 [132–307]	273 [188–362]	**<0.001**
Random glucose, mmol/L		5.5 [4.9–6.6]	5.2 [4.7–6.0]	**0.009**
Venous lactate, mmol/L		2.3 [1.6–3.3]	1.7 [1.3–2.4]	**<0.001**
C-reactive protein, mg/L		179 [115–266]	148 [88–224]	**0.001**
Procalcitonin, μg/L		6.3 [1.3–31.2]	1.7 [0.3–6.6]	**<0.001**
^2^D-dimer, mg/L		2.4 [1.1–4.3]	1.2 [0.9–2.9]	**<0.001**
Alanine aminotransferase, U/L		24.5 [14.3–40.0]	27.0 [16.0–49.0]	0.229
Alkaline phosphatase, U/L		140.0 [96.0–218.5]	108.5 [77.0–170.3]	**<0.001**
Total bilirubin, μmol/L		9.0 [6.0–15.8]	7.0 [5.0–11.0]	**0.016**
Conjugated bilirubin, μmol/L		5.0 [3.0–9.0]	4.0 [3.0–7.0]	**0.018**
Total protein, g/L		73.0 [64.8–80.0]	76.0 [68.0–85.0]	**<0.001**
Albumin, g/L		22.0 [18.0–26.0]	25.0 [22.0–29.0]	**<0.001**
Creatinine, μmol/L		101.0 [63.8–183.5]	77.0 [59.0–106.5]	**<0.001**
CMV detectable		58 (49.6)	159 (36.4)	**0.011**
Serum CRAG lateral flow test	Positive	3 (2.4)	16 (3.6)	0.602
^3^*Mycobacterium tuberculosis* cultured from blood		64 (53.3)	153 (36.0)	**0.002**
^4^Urine lateral flow LAM test	Positive (≥1 by two readers)	46 (37.1)	150 (33.9)	0.287
^5^Urine Xpert MTB/RIF assay positive		58 (59.2)	164 (42.1)	**0.003**
Microbiologically confirmed tuberculosis		108 (87.1)	371 (83.7)	0.292
Rifampicin resistance on any clinical sample		21 (16.9)	32 (7.2)	**0.003**

Nine participants were lost to follow-up and not included in this table.

Categorical variables presented as count and percentage, *n* (%).

Continuous variables presented as median with IQR: median [IQR].

*p*-value comparing deaths to survivors using the Fisher exact test for categorical data or Wilcoxon rank sum test for continuous variables.

^1^Participants also started on other antituberculosis therapy regimens: *n* = 11 started on renally adjusted treatment regimen (ethambutol given on alternative days), *n* = 3 started a regimen adjusted due to liver dysfunction, and *n* = 4 started on rifabutin-containing regimens.

^2^D-dimer values missing for 100 participants.

^3^Percentages calculated for *n* = 554. No TB blood culture performed in 22 participants.

^4^No urine sample taken, or urine lateral flow LAM test not performed in 72 participants.

^5^ Percentages calculated for *n* = 497. No urine sample taken, or urine Xpert MTB/RIF assay not performed in 79 participants.

Abbreviations: ART, antiretroviral treatment; CD4, cluster of differentiation 4; CMV, cytomegalovirus; CRAG, cryptococcal antigen; LAM, lipoarabinomannan; TB, tuberculosis.

### Co-morbidities, treatment access, and drug-resistant tuberculosis

In univariable comparisons, participants who died were older and had lower CD4, monocyte, and lymphocyte counts, but there was no difference in HIV viral load. Only 210/576 (36.5%) were taking ART and 70/576 (12.2%) were virologically suppressed, with no difference between outcome groups (15/124, 12.1% in those who died versus 53/443, 12.0%, *p* = 1.000). At the week 12 follow-up, 84% of the 443 survivors were on ART.

Antituberculosis therapy was started in 566/576 (98.3%) of participants, and 487/576 (84.5%) of participants started therapy within 48 hours of enrolment. Antituberculosis therapy was initiated after discharge from hospital in 19/576 (3.3%) participants. Ten participants 10/576 (1.7%) died before antituberculosis therapy was initiated, and these participants died early at a median of 2.5 days (IQR, 1–5 days) after enrolment.

There were 55 participants with rifampicin resistance: a significantly higher proportion who died had rifampicin resistance, 21/124 (16.9%), compared with 32/443 (7.2%) survivors, *p* = 0.002. For these participants, the initial antituberculosis therapy regimen was drug-resistant antituberculosis therapy in 19/55 (34.5%) (Table 2). We did not capture the switch to drug-resistant therapy after the initial treatment regimen but documented all medication received after enrolment. By the end of study follow-up, loss to follow-up, or death, therapy for drug-resistant tuberculosis was initiated in 74.6%–83.6% of participants as indicated by receipt of fluoroquinolone (46/55, 83.6%) or terizidone (41/55, 74.6%). Mortality in the rifampicin-resistant participants was 21/55 (38.2%).

To assess the contribution of bacterial infections to mortality, we reviewed results of all bacterial blood cultures taken during the enrolment admission. Bacterial blood cultures were performed in 296/576 (51.4%) participants, and 7/296 (2.4%) cultured a pathogen other than *M*. *tuberculosis* during the enrolment admission (1 *Cryptococcus neoformans*, 1 *Candida albicans*, 1 *Staphylococcus aureus*, 4 gram-negative bacteria); all seven had microbiologically confirmed tuberculosis. Four died: 1 with *S*. *aureus* and 3 with gram-negative bacteremia. Urine, pleural, and cerebrospinal fluid bacterial cultures performed in routine service yielded few positive results, with no difference between outcome groups (S5 Table). Sputum bacterial cultures yielded 70/312 (22%) positive results, with 45/70 (64%) showing oral mixed flora, and the remaining positive results were not regarded as clinically significant in most cases (S5 Table).

Ceftriaxone was administered to 505/576 (87.7%) participants and 83/576 (14.4%) received the initial dose at a primary health clinic prior to transfer to hospital and thus before blood culture.

Serum lateral flow CRAG test was positive in 19 participants, and there was no difference in mortality in those who were CRAG test positive or negative (Table 2). CMV viral load was detectable in 223/576 (38.7%) participants, and a higher proportion was detectable in those who died (58/124, 49.6%) compared with survivors (159/443, 36.4%), *p* = 0.011. However, having a detectable CMV viral load was not independently associated with death in a Cox proportional hazards analysis after adjusting for potential confounders, including CD4 count (S3 Table).

Eight participants had clinical Kaposi sarcoma at the time of enrolment; of these, five had microbiologically confirmed tuberculosis, three had probable tuberculosis, and all eight survived.

Participants with a clinical diagnosis of *Pneumocystis jirovecii* pneumonia (PJP) were classified as no tuberculosis and excluded. Participants who were treated for PJP and tuberculosis simultaneously were classified as having possible tuberculosis and excluded from analyses (S2 Table).

### Dissemination of tuberculosis

**Fig 2:**
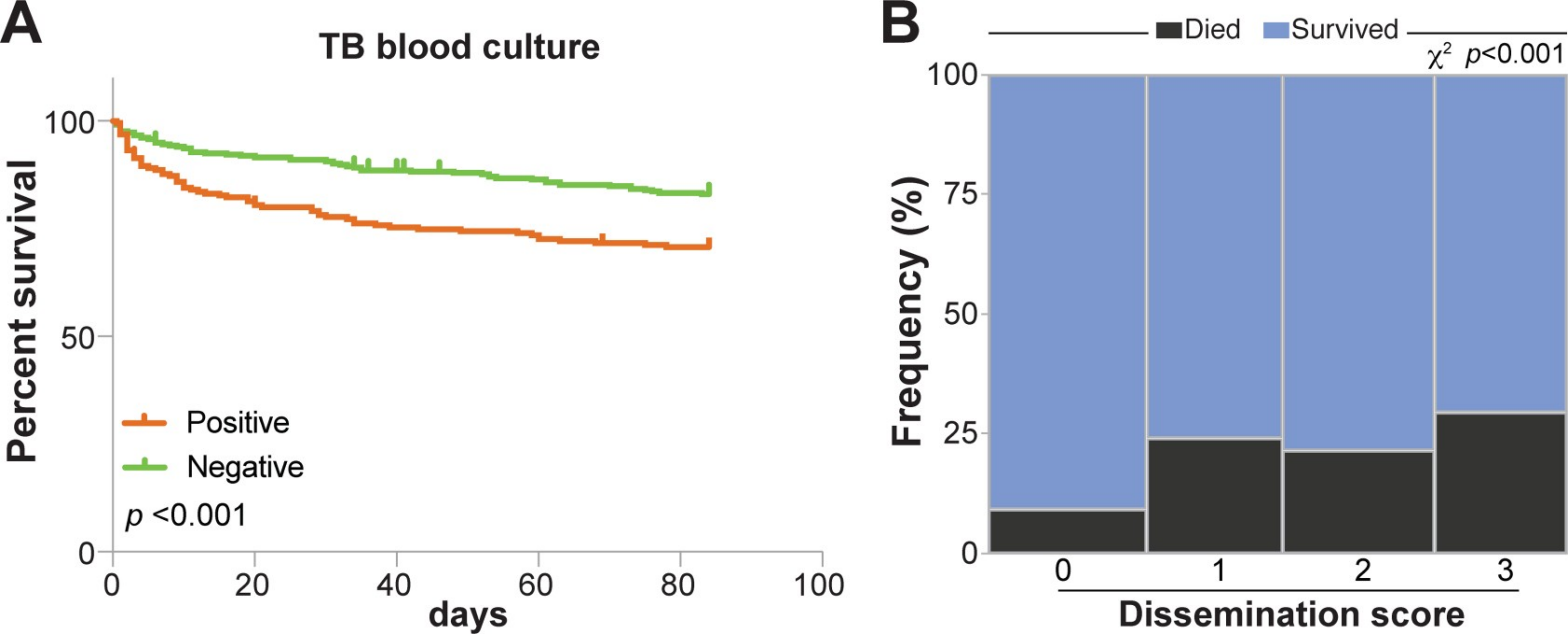
Association between tuberculosis dissemination and 12-week mortality. **(A)** Kaplan Meier curve showing percentage survival over 84 days for participants who tested positive for *Mycobacterium tuberculosis* blood culture versus those who tested negative. Curves were compared using log-rank (Mantel-Cox) test. **(B)** Frequencies of individuals who died, stratified by tuberculosis dissemination score values, were compared using the Pearson chi-squared test with linear trend. TB, tuberculosis.

MTB BSI was diagnosed in 38.2% (220/576) of participants and in a significantly higher proportion of participants who died (51.6%, 64/124) versus survived (34.5%, 153/443) (*p* = 0.002) (Table 2 and Fig 2A).

A three-point dissemination score was used to explore tuberculosis dissemination further. A score was allocated to *n* = 457 (*n* = 93 deaths and *n* = 364 survivors) participants who had valid results for all three tests and known outcomes at 12 weeks. A high proportion of participants 295/457 (64.6%) had a tuberculosis dissemination score of ≥1. The distribution of the scores was as follows: a tuberculosis dissemination score of 0 in 162/457 (35.4%), a score of 1 in 106/457 (23.2%), a score of 2 in 102/457 (22.3%), and a score of 3 in 87/457 (19.0%). Of participants who died, 74/93 (79.6%) had a tuberculosis dissemination score of ≥1 versus 221/364 (60.7%) of survivors, *p* = 0.001. We explored risk of mortality according to dissemination score; a higher dissemination score was associated with an increased risk of mortality (Figs 2B and S1, panel 3).

### Features of sepsis syndrome

Many participants were acutely ill with features compatible with sepsis [32]. One third (195/576, 33.9%) had elevated lactate, which indicates strained cellular metabolism in sepsis [6,33]. A higher proportion of participants who died (63/124, 50.8%) compared with survivors (128/443, 28.9%, *p* < 0.001) had elevated lactate (Table 2). Similarly, in survival analysis, those with high lactate had significantly higher mortality (S1 Fig). Also compatible with sepsis syndrome was renal impairment (creatinine > 104 μmol/L), seen in 34.7% (177/576) of participants. Additional features that are used in the sequential organ failure assessment score (SOFA) to identify sepsis [32], such as elevated total bilirubin (>20 μmol/L), seen in 9.2% (53/576); decreased platelets (<150 × 10^9^/L), seen in 18.1% (104/576); and decreased level of consciousness (Glasgow Coma Scale <15), which was observed in 13.2% (76/576), are nonspecific in this context and may also be attributable to tuberculosis organ involvement. All these abnormalities were more common in participants who died (Table 2). Markers of inflammation used in clinical practice to monitor sepsis syndrome, such as C-reactive protein, procalcitonin, and D-dimer, were elevated in 557/576 (96.7%), 413/576 (71.7%), and 469/476 (98.5%) of participants, respectively, and were significantly higher in those who died (Table 2).

### Host soluble mediators of inflammation and mortality

**Fig 3:**
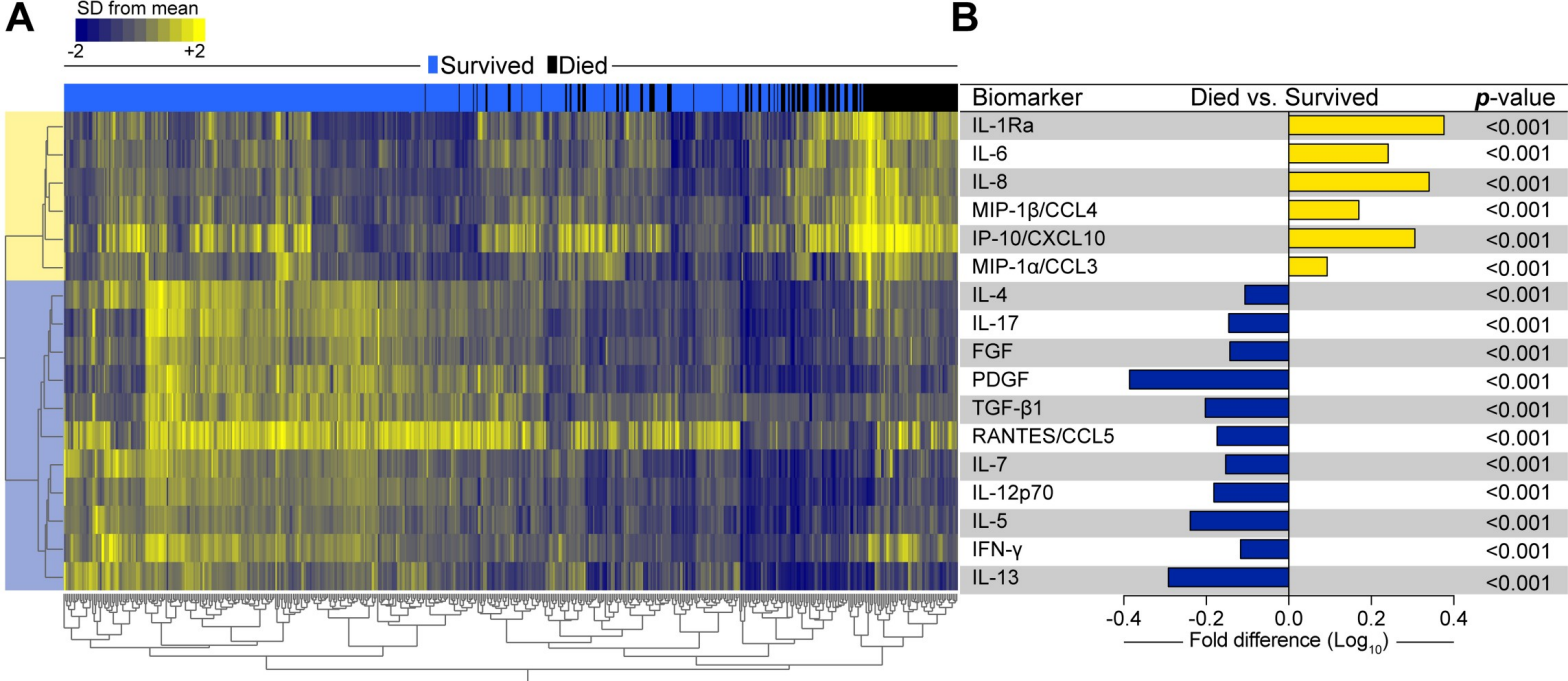
Host soluble inflammatory mediators associated with mortality in participants with hospitalized HIV-associated tuberculosis. **(A)** Values of inflammatory mediators were log transformed and z-score normalized. A non-supervised two-way hierarchical cluster analysis (Ward method with 100× bootstrap) was employed to test if simultaneous assessment of indicated mediators could group separately individuals that died from those who survived. Only mediators that were statistically different between the study groups after adjustment for multiple measurements (Holm-Bonferroni method) are shown. Data on other mediators are shown in S4 Table and S2 and S3 Figs. **(B)** Bars represent fold-difference values between participants that died versus those who survived, with Holm-Bonferroni *p*-values. Yellow bars indicate mediators that were significantly higher, whereas blue bars highlight mediators that were lower in participants who died compared with those who survived. FGF, basic fibroblast growth factor; IFNγ, interferon gamma; IL, interleukin; IL-1Ra, IL-1 receptor antagonist; IP-10, interferon gamma-induced protein; CXCL10, C-X-C motif chemokine ligand 10; MIP-1α, macrophage inflammatory protein-1 alpha; CCL3, C-C motif chemokine ligand 3; MIP-1β, macrophage inflammatory protein-1 beta; CCL4, C-C motif chemokine ligand 4; PDGF, platelet-derived growth factor; RANTES, regulated on activation, normal T-cell expressed and secreted; CCL5, C-C motif chemokine ligand 5; TGF-β1, transforming growth factor beta 1.

Many soluble mediators of inflammation were significantly elevated or reduced in univariable comparison (corrected for multiple comparisons) of participants who died to those who survived (Table 3 and S2 and S3 Figs). We compared fold differences in analyte values between participants who died and those who survived. In non-supervised two-way hierarchical cluster analysis, the mediators segregated most participants who died from survivors (Fig 3A). Biological pathways of mediators, which were significantly increased in participants who died (Figs 3B and S2), were related to the innate immune system and chemotactic signaling (IL-8, MIP-1β/CCL4, IP-10/CXCL10, and MIP-1α/CCL3), anti-inflammatory (IL-1Ra), and proinflammatory (IL-6). IL-1Ra was 8-fold higher in early deaths compared with survivors (median fluorescence intensity was 1,417 in early deaths versus 169.5 in survivors) (S4 Table). Functions of mediators that were significantly lower in participants who died (Figs 3B and S3) can be broadly classified as T-cell associated (IL-4, IL-17, RANTES/CCL5, IL-7, IL-12p70, IL-5, IFN-γ, IL-13) and growth factors (FGF, PDGF, TGF-β1).

**Table 3:** Host soluble mediators of inflammation fluorescence intensity values in hospitalized participants with HIV-associated tuberculosis: Comparison between participants who died within 12 weeks and survivors.

Host soluble mediators of inflammation	Deaths	Survivors	*p*	Holms-Bonferroni *p*
	*n* = 108	*n* = 391		
**Higher in participants who died**				
IL-8	211.5 (110.4–410.8)	110.0 (78.5–165.5)	<0.001	<0.001
MIP-1β/CCL4	1,076.0 (570.5–2,501.0)	624.5 (397.5–1,087.5)	<0.001	<0.001
IL-1Ra	449.8 (145.1–1,425.3)	169.5 (93.0–397.5)	<0.001	<0.001
IL-6	361.3 (194.4–656.8)	208.0 (119.3–359.8)	<0.001	<0.001
IP-10/CXCL10	10,818.0 (6,326.9–16,913.8)	6,495.0 (3,301.5–11,846.3)	<0.001	<0.001
MIP-1α/CCL3	129.0 (73.0–295.0)	93.0 (65.8–156.3)	0.001	**0.027**
**Lower in participants who died**				
IL-5	22.00 (15.0–30.2)	31.0 (22.0–43.5)	<0.001	<0.001
RANTES/CCL5	12,688.0 (7,340.8–15,191.9)	15,369.5 (12,732.5–16,552.3)	<0.001	<0.001
IL-13	27.0 (18.0–39.8)	39.0 (29.0–59.5)	<0.001	<0.001
PDGF	93.5 (56.4–199.1)	201.0 (84.0–418.5)	<0.001	<0.001
FGF	45.3 (37.0–54.0)	54.0 (43.8–69.0)	<0.001	<0.001
IL-7	28.5 (22.0–37.0)	35.0 (28.0–45.3)	<0.001	<0.001
IL-12p70	44.5 (35.4–58.1)	56.0 (42.0–76.8)	<0.001	<0.001
IL-4	38.8 (26.8–55.1)	48.0 (36.8–63.3)	<0.001	<0.001
*TGF-β1	16.5 (12.0–36.2)	26.4 (15.7–55.4)	<0.001	**0.006**
IL-17	56.0 (41.8–78.3)	64.5 (48.8–90.3)	<0.001	**0.019**
IFNγ	45.0 (29.8–66.0)	54.0 (39.0–74.5)	0.001	**0.031**
**No statistically significant difference between participants who died and those who survived**				
TNFα	38.5 (30.0–52.5)	43.5 (36.0–54.3)	0.007	0.210
IL-2	62.3 (49.8–77.4)	68.0 (55.3–81.0)	0.019	0.522
MCP-1/CCL2	108.0 (76.5–159.5)	95.5 (75.0–138.0)	0.036	0.999
GM-CSF/CSF2	84.00 (64.5–109.3)	89.5 (72.0–113.0)	0.062	1.000
Eotaxin	61.3 (43.8–86.1)	66.0 (53.0–88.3)	0.065	1.000
IL-9	175.3 (113.9–243.0)	153.0 (121.0–205.0)	0.113	1.000
VEGF	107.0 (72.0–143.0)	107.0 (78.8–158.8)	0.237	1.000
G-CSF/CSF3	75.5 (47.8–117.1)	67.0 (54.0–90.5)	0.314	1.000
IL-15	90.0 (73.0–115.0)	89.5 (74.0–114.3)	0.923	1.000
IL-1β	64.0 (47.5–85.8)	64.0 (50.0–84.5)	0.950	1.000
IL-10	68.5 (51.5–91.5)	69.0 (55.0–85.0)	0.961	1.000

Host soluble mediators of inflammation were measured on stored plasma in a random selection of participants with HIV-associated tuberculosis (*n* = 507: *n* = 108 deaths, *n* = 391 survivors, *n* = 8 lost to follow-up) using the Biorad Bioplex 200 Luminex platform and fluorescence intensity values are presented.

*TGF-β1 concentrations were measured with ELISA and are presented in picograms per milliliter. Inflammatory mediators are arranged into three groups: mediators that were higher in participants who died, mediators that were lower in participants who died, and mediators that showed no difference between survival groups. Each group is ranked from lowest to highest *p*-values. Comparisons of participants who died with those who survived were made using the Wilcoxon rank sum test. *p*-values were corrected for multiple comparisons with Holms-Bonferroni correction. Bold *p*-values indicate mediators that remained significantly different after correction for multiple comparisons.

Abbreviations: CCL, C-C motif chemokine ligand; CSF2, colony stimulating factor 2; CSF3, colony stimulating factor 3; CXCL, C-X-C motif chemokine ligand; FGF, basic fibroblast growth factor; G-CSF, granulocyte-colony stimulating factor; GM-CSF, granulocyte-macrophage colony-stimulating factor; IFNγ, interferon gamma; IL, interleukin; IL-1Ra, IL-1 receptor antagonist; IP-10, interferon gamma-induced protein; MCP, monocyte chemoattractant protein; MIP, macrophage inflammatory protein; PDGF, platelet-derived growth factor; RANTES, regulated on activation, normal T-cell expressed and secreted; TGF-β1, transforming growth factor beta 1; TNFα, tumor necrosis factor alpha; VEGF, vascular endothelial growth factor.

### Variance of host soluble inflammatory mediators

**Fig 4:**
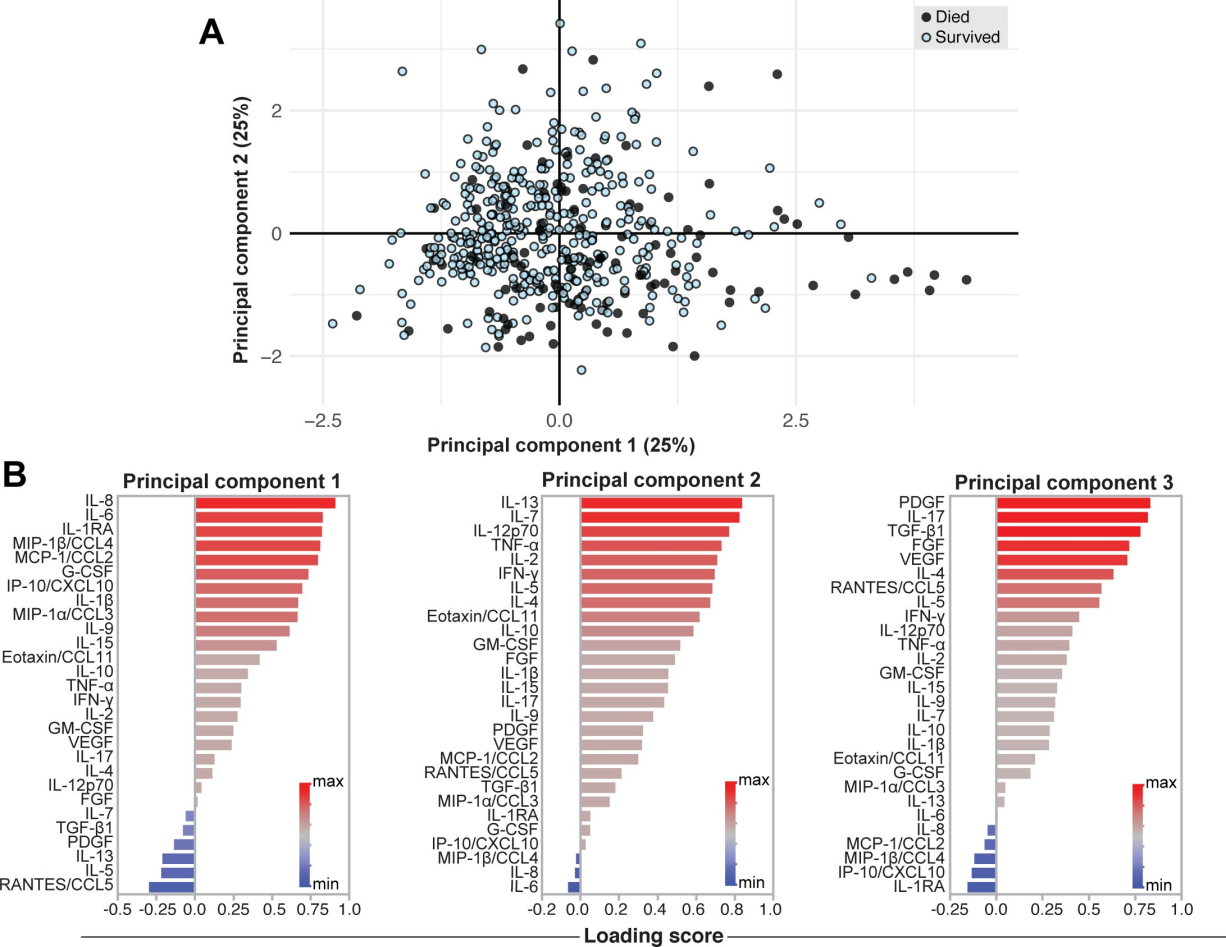
Principal components analysis of host soluble inflammatory mediators. **(A)** Principal components analysis with rotation was used to analyze the inflammatory mediators and explain the variance of the data distribution in the cohort. Participants are represented by dots and colored by outcome. The two axes represent principal components 1 (PC1 on the x-axis) and 2 (PC2 on the y-axis), and their contribution to the total data variance is shown as a percentage. PC3 contributed 19% of total variance and is not shown. **(B)** Variables contributing to PC1, PC2, and PC3 are shown with red bars indicating positive weighting and blue bars indicating negative weighting. CCL, C-C motif chemokine ligand; CSF2, colony stimulating factor 2; CSF3, colony stimulating factor 3; CXCL, C-X-C motif chemokine ligand; FGF, basic fibroblast growth factor; G-CSF, granulocyte-colony stimulating factor; GM-CSF, granulocyte-macrophage colony-stimulating factor; IFNγ, interferon gamma; IL, interleukin; IL-1RA, IL-1 receptor antagonist; IP-10, interferon gamma-induced protein; MCP, monocyte chemoattractant protein; MIP, macrophage inflammatory protein; PC, principal component; PDGF, platelet-derived growth factor; RANTES, regulated on activation, normal T-cell expressed and secreted; TGF-β1, transforming growth factor beta 1; TNFα, tumor necrosis factor alpha; VEGF, vascular endothelial growth factor.

To explore covariance in host inflammatory mediators, we used principal components analysis with maximal rotation. Two major rotated principal components, principal component 1 and principal component 2 (PC1 and PC2) were identified, which accounted for 25% of total variability each (Fig 4A). Principal component 3 (PC3) accounted for 19% of variability. Functions of inflammatory mediators responsible for positive weighting in PC1 can be classified as mediators of the innate immune system and chemotaxis (IL-8, MIP-1β/CCL4, and MCP-1/CCL2), proinflammatory (IL-6), and anti-inflammatory (IL-1Ra). Inflammatory mediators responsible for positive weighting of PC2 were mainly T-cell associated (IL-13, IL-7, IL-12p70, TNF-α, IL-2, and IFN-γ). Mediators that contributed most to PC3 weighting were growth factors (PDGF, TGF-β1, FGF, VEGF) and T-cell associated (IL-17, IL-4) (Fig 4B).

**Fig 5:**
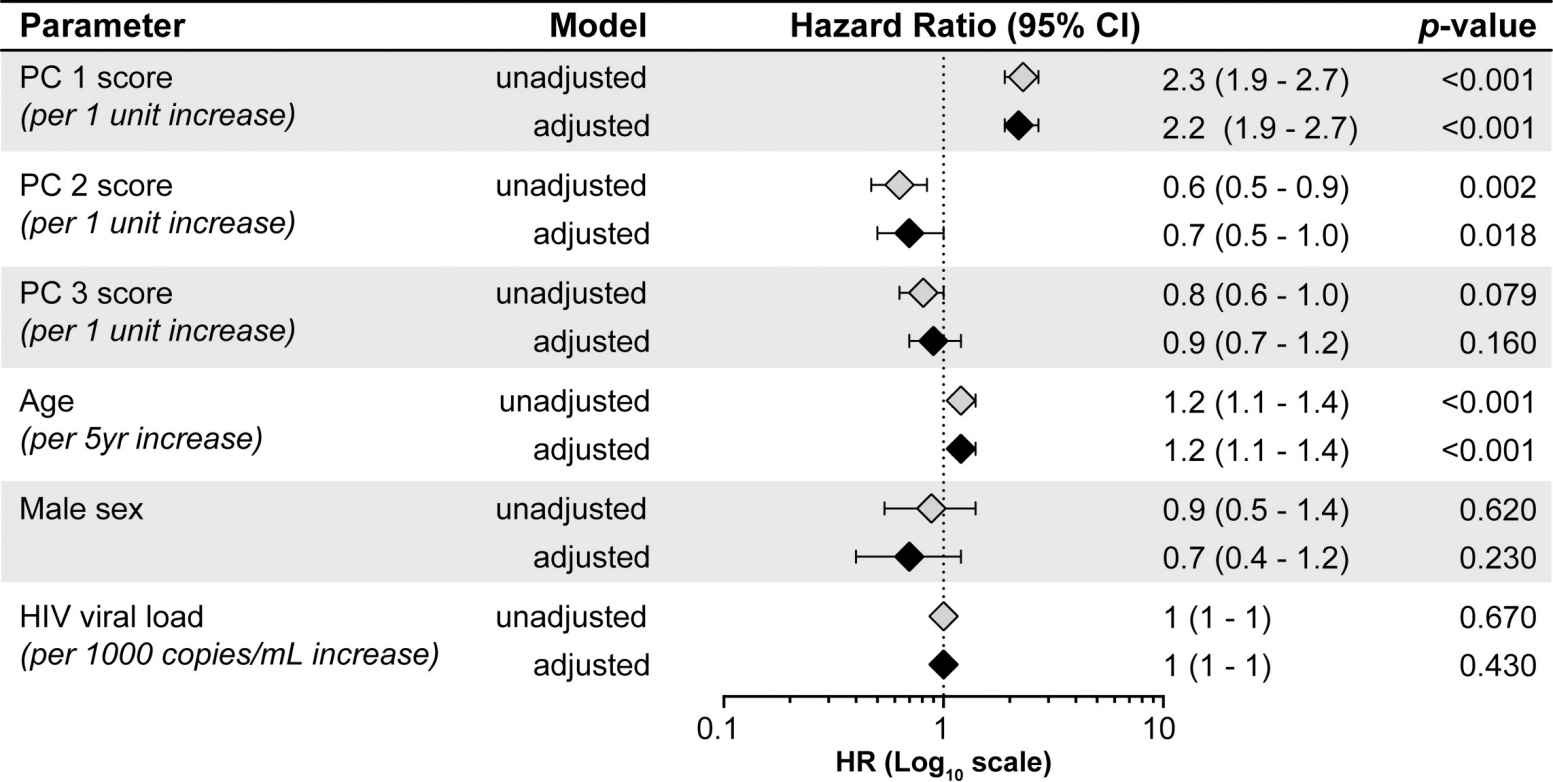
Cox-regression analysis to evaluate association between principal components score and 28-day mortality. Cox regression analysis was conducted with each variable individually (unadjusted) and then all variables were included in a multivariable model (adjusted). Age, sex, and HIV viral load were incorporated a priori to adjust for patient specific variance and HIV-related factors. The model was censored at 28 days to meet the proportional hazards assumption, and the global proportional hazards test for the multivariable model result was *p* = 0.43. PC, principal component.

To further explore the association of these immune profiles with mortality, the principal components (PC1, PC2, and PC3) were incorporated as variables in a Cox proportional hazards model (Fig 5). PC1 was independently associated with mortality, aHR = 2.2 (95% CI = 1.9–2.7, *p* < 0.001), and PC2 was protective (aHR = 0.7 (95% CI = 0.5–1.0, *p* = 0.018). PC3 was not associated with mortality.

A sensitivity analysis excluding patients who died within 7 days yielded similar results. Amongst *n* = 472 patients, three principal components explained the majority of the variation. PC1 explained 26% of variance and was dominated by IL-13, IL-7, IL-12p70, TNF-α, IL-2, and IFN-γ. PC1 was protective, with HR for death = 0.63 (95%CI = 0.41–0.98, *p* = 0.038) in univariate analysis and aHR = 0.69 (95%CI = 0.45–1.05, *p* = 0.080). PC2 explained 22% of variance and was dominated by IL-8, IL-6, IL-1Ra, MIP-1β/CCL4, MCP-1/CCL2, and IP10/CXCL10. PC2 was associated with mortality with HR = 1.6 (95%CI = 1.2–2.2), *p* = 0.002) in univariate analysis and aHR = 1.57 (95%CI = 1.17–2.11, *p* = 0.003). PC3 explained 20% of variance and was dominated by PDGF, IL-17, TGFB-1, RANTES/CCL5, FGF, and VEGF. PC3 was not associated with mortality, with HR = 0.93 (95%CI = 0.65–1.3, *p* = 0.680) in univariate analysis and aHR = 1.03 (95%CI = 0.69–1.53, *p* = 0.880).

### Association between host inflammatory mediators and leucocyte counts

**Fig 6:**
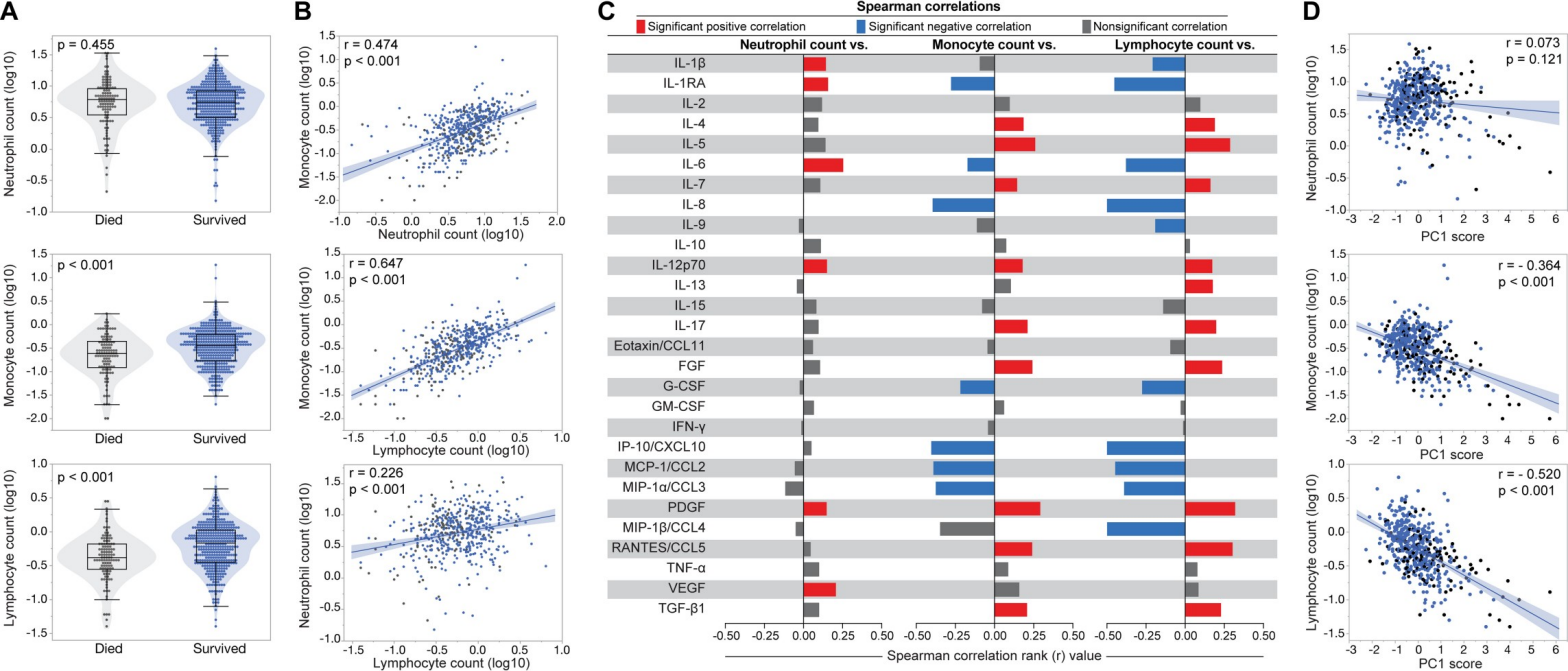
Associations between leukocyte counts in peripheral blood and systemic inflammation. **(A)** Absolute counts of indicated leukocytes were compared between the participants who died versus those who survived. Data are represented with violin plots, with scatter dots and box and whiskers indicating median values, IQRs, and maximum and minimum values excluding outliers, respectively. Groups were compared using the Mann-Whitney *U* test. **(B)** Spearman correlations between indicated cell counts are shown. Linear curve fit (with 95% CI) was used to illustrate trends of data distribution. **(C)** Spearman correlation analysis between cell counts and inflammatory mediator values in plasma. **(D)** Spearman correlations between indicated cell counts and PC1 score values are shown. Linear curve fit (with 95% CI) was used to illustrate trends of data distribution. CCL, C-C motif chemokine ligand; CSF, colony stimulating factor; CXCL, C-X-C motif chemokine ligand; FGF, basic fibroblast growth factor; G-CSF, granulocyte-colony stimulating factor; GM-CSF, granulocyte-macrophage colony-stimulating factor; IFNγ, interferon gamma; IL, interleukin; IL-1RA, IL-1 receptor antagonist; IP-10, interferon gamma-induced protein; MCP, monocyte chemoattractant protein; MIP, macrophage inflammatory protein; PC1, principal component 1; PDGF, platelet-derived growth factor; RANTES, regulated on activation, normal T-cell expressed and secreted; TGF-β1, transforming growth factor beta 1; TNFα, tumor necrosis factor alpha; VEGF, vascular endothelial growth factor.

Many of the inflammatory mediators are secreted by leucocytes. Participants who died had significantly lower lymphocyte and monocyte counts, but neutrophil counts were similar to those who survived (Figs 6A and S4). We explored the association of cell counts with each other and with soluble inflammatory mediators. Cell counts had significant positive correlations with each other (Fig 6B). We plotted the Spearman correlation coefficient of each inflammatory mediator value with the neutrophil, monocyte, and lymphocyte counts (Fig 6C). Monocyte and lymphocyte counts showed significant negative correlations with IL-1Ra, IL-6, and mediators of chemotactic signaling IL-8, MIP-1β/CCL4, IP-10/CXCL10, and MIP-1α/CCL3. We explored the association of PC1 score with cell counts (Fig 6D). Lymphocytes and monocytes had significant negative correlation with PC1 score. PC2 was dominated by T-cell associated mediators, and we explored the association of PC2 with lymphocyte count and CD4 cell count. PC2 and lymphocytes had a weak positive correlation (r = 0.1, *p* = 0.021) and PC2 and CD4 cell count were not correlated (r = 0.06, *p* = 0.196) (S5 Fig).

### Dissemination of tuberculosis and soluble mediators of inflammation

**Fig 7:**
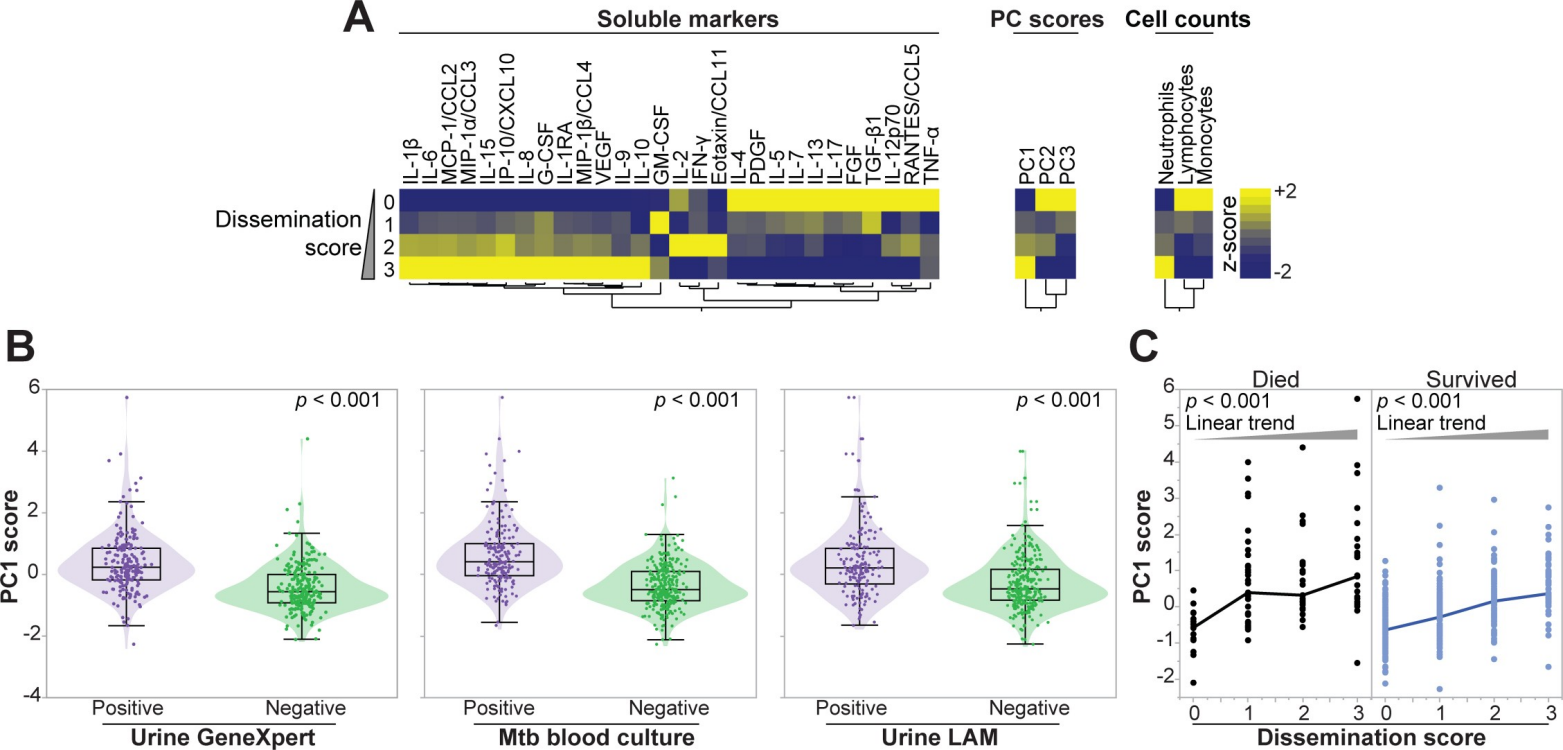
Associations between tuberculosis dissemination score, components thereof, and host soluble mediators of inflammation. **(A)** Mean values of log-transformed value of each plasma mediator per TB dissemination score values were calculated for all participants who had all three tests performed (urine LAM test, urine Xpert MTB/RIF test, mycobacterial blood culture), *n* = 457. Inflammatory mediator values were z-score normalized and illustrated in a heatmap in which inflammatory mediators were grouped using hierarchical clustering (Ward method with 100× bootstrap). Dendrograms represent Euclidean distance. **(B)** PC1 score values were compared between those who tested positive and negative for each of the three tests used to calculate the tuberculosis dissemination score using the Mann-Whitney *U* test. **(C)** PC1 score values were compared between participants presenting with increasing tuberculosis dissemination scores from 0 to 3 in both outcome groups, and values were compared using the Kruskal-Wallis test with the nonparametric linear trend ad hoc test. Lines connect median values. CCL, C-C motif chemokine ligand; CSF, colony stimulating factor; CXCL, C-X-C motif chemokine ligand; FGF, basic fibroblast growth factor; G-CSF, granulocyte-colony stimulating factor; GM-CSF, granulocyte-macrophage colony-stimulating factor; IFNγ, interferon gamma; IL, interleukin; IP-10, interferon gamma-induced protein; LAM, lipoarabinomannan; MCP, monocyte chemoattractant protein; MIP, macrophage inflammatory protein; MTB, *Mycobacterium tuberculosis*; PC, principal component; PDGF, platelet-derived growth factor; RANTES, regulated on activation, normal T-cell expressed and secreted; TB, tuberculosis; TGF-β1, transforming growth factor beta 1; TNFα, tumor necrosis factor alpha; VEGF, vascular endothelial growth factor.

Soluble inflammatory mediator values were calculated per tuberculosis dissemination score (*n* = 457 participants who had a tuberculosis dissemination score calculated) and illustrated in a heatmap using hierarchical clustering to determine which mediators grouped together (Fig 7A). Higher values of proinflammatory mediators associated with the innate immune response (IL-1β and IL-6), mediators of chemotactic signaling (IL-8, MIP-1β/CCL4, IP-10/CXCL10, MIP-1α/CCL3), and anti-inflammatory mediators (IL-1Ra, IL-10) grouped together and were higher in participants with a dissemination score of three. Higher PC1 score and neutrophil count, and lower lymphocyte and monocyte counts were associated with a high dissemination score.

Higher values of T-cell associated mediators (IL-4, -5, -7, -13, -17, -12p70; RANTES/CCL5, TNF-α) and growth factors (PDGF, FGF, and TGF-β1) grouped together in participants with a tuberculosis dissemination score of zero.

Next, we evaluated PC1 score values stratified by those who tested positive and negative for each of the three biomarkers that were used to calculate the tuberculosis dissemination score, and PC1 score was significantly higher in those who tested positive for each test (Fig 7B). Irrespective of outcome, PC1 score increased significantly with increased dissemination score (Fig 7C).

### Association of inflammatory mediators with time to death

**Fig 8:**
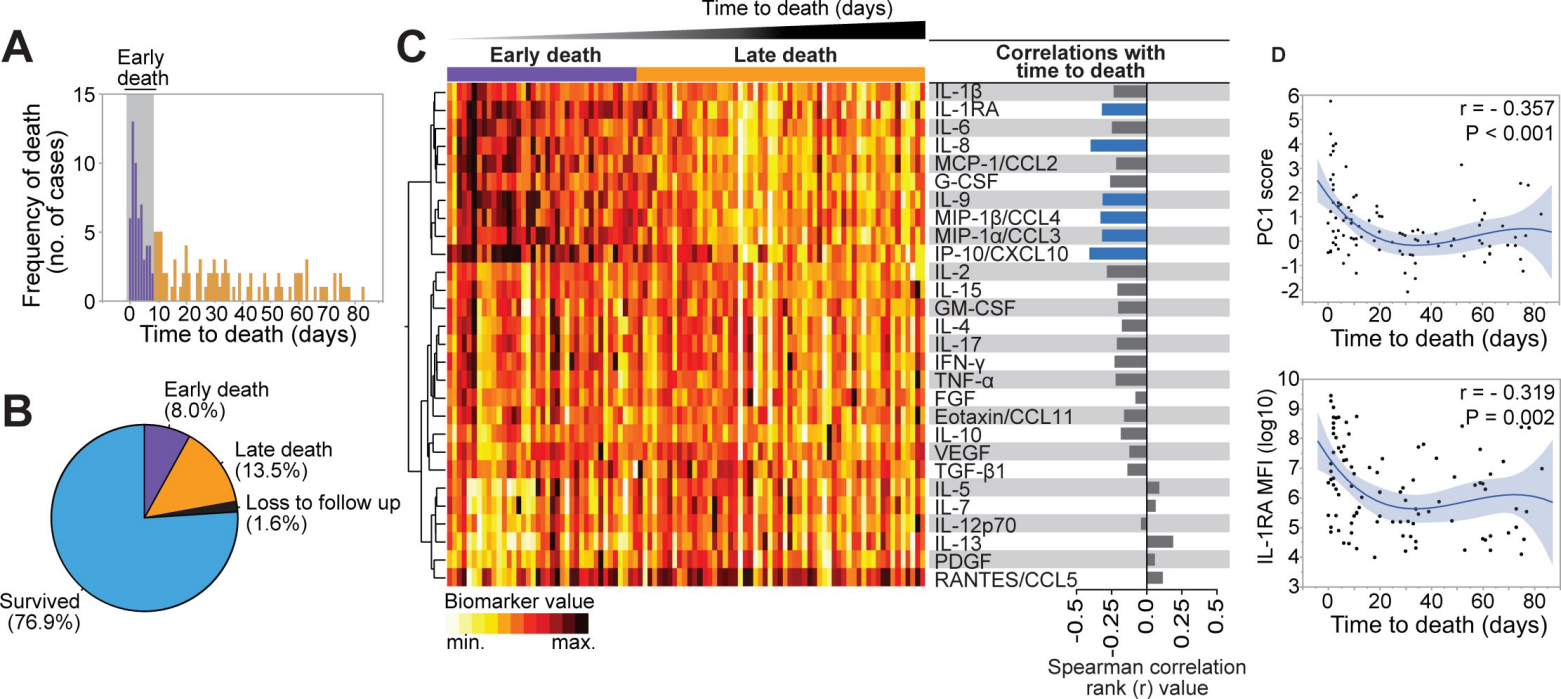
Inverse correlations between host soluble mediators of inflammation and time to death. **(A)** Histogram shows the frequency of participants who died over time. **(B)** Pie chart shows the frequency of participants who survived, those who died within 7 days of admission (early death), and those who died after 7 days (late death). **(C)** Left panel: data were log transformed and ranked and colored in a heatmap from minimum to maximum values detected for each inflammatory mediator. Participants were ordered based on time to death (in days), and plasma inflammatory mediators were clustered (Ward method with 100× bootstrap) according to the distribution profile in the study population. Dendrograms represent Euclidean distance. Right panel: Spearman correlations for each mediator and time to death. Blue bars indicate statistically significant correlations (which were all negative) after corrections for multiple measurements (Holm-Bonferroni method). **(D)** Spearman correlations between PC1 score values and IL1-Ra mean fluorescence intensity values and time to death are shown. Nonlinear curve fit (quadratic, with 95% CI) was used to illustrate trends of data distribution. CCL, C-C motif chemokine ligand; CXCL, C-X-C motif chemokine ligand; FGF, basic fibroblast growth factor; G-CSF, granulocyte-colony stimulating factor; GM-CSF, granulocyte-macrophage colony-stimulating factor; IFNγ, interferon gamma; IL, interleukin; IL1-Ra, IL-1 receptor antagonist; IP-10, interferon gamma-induced protein; max., maximum; MCP, monocyte chemoattractant protein; MFI, mean fluorescence intensity; min., minimum; MIP, macrophage inflammatory protein; no., number; PC1, principal component 1; PDGF, platelet-derived growth factor; RANTES, regulated on activation, normal T-cell expressed and secreted; TGF-β1, transforming growth factor beta 1; TNFα, tumor necrosis factor alpha; VEGF, vascular endothelial growth factor.

The distribution of time to death is shown in Fig 8A and 8B. Host soluble inflammatory mediators were explored in relation to time to death. Participants were ordered based on time to death, and inflammatory mediators were ranked and colored from minimum to maximum values and illustrated in a heatmap. Higher values of certain inflammatory mediators grouped together (Fig 8C) in early deaths, and these mediators had significant negative correlations with time to death. These mediators were the anti-inflammatory IL-1Ra, mediators associated with the innate immune system, and chemotactic signaling (IL-8, MIP-1α/CCL3, MIP-1β/CCL4, IP-10/CXCL10) and one T-cell associated mediator (IL-9). We also explored the relationship between IL-1Ra and PC1 score with time to death, and both had significant nonlinear negative correlations with time to death (Fig 8D).

### Inflammatory profile of early deaths and late deaths

**Fig 9:**
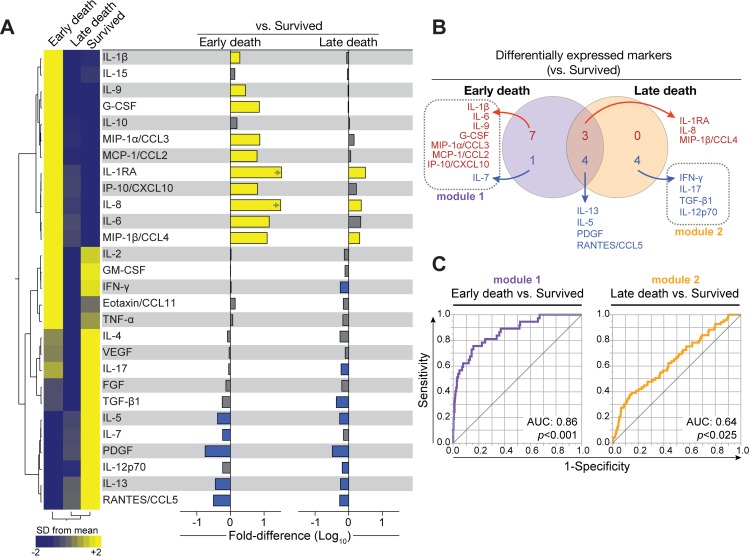
Participants who died early after admission exhibited a distinct inflammatory profile in plasma. **(A)** Left panel: mean values of log-transformed values of each soluble inflammatory mediator were calculated for early deaths (within 7 days of enrolment) and late deaths (after 7 days, within 12 weeks). Values were z-score normalized and illustrated in a heatmap in which inflammatory mediators were grouped using hierarchical clustering (Ward method with 100× bootstrap). Dendrograms represent Euclidean distance. Right panel: bars represent fold-difference values between participants who died early or late versus those who survived. Yellow bars indicate mediators that were significantly higher, whereas blue bars highlight mediators that were significantly lower in the groups of participants who died compared with those who survived, after adjustments for multiple measurements (Holm-Bonferroni method). Arrows indicate values higher than the upper limit of the axis. **(B)** Venn diagrams illustrate the inflammatory mediators that were significantly different between participants who died and survived. Mediators indicated in red were higher, whereas those in blue were lower in the groups of participants who died versus participants who survived. **(C)** ROC curve analyses of the combination of uniquely expressed mediators (module 1 or module 2) were used to test the power to predict early or late mortality versus survival. AUC, area under the curve; CCL, C-C motif chemokine ligand; CXCL, C-X-C motif chemokine ligand; FGF, basic fibroblast growth factor; G-CSF, granulocyte-colony stimulating factor; GM-CSF, granulocyte-macrophage colony-stimulating factor; IFNγ, interferon gamma; IL, interleukin; IL-1RA, IL-1 receptor antagonist; IP-10, interferon gamma-induced protein; MCP, monocyte chemoattractant protein; MIP, macrophage inflammatory protein; PDGF, platelet-derived growth factor; RANTES, regulated on activation, normal T-cell expressed and secreted; ROC, receiver operating characteristic; TGF-β1, transforming growth factor beta 1; TNFα, tumor necrosis factor alpha; VEGF, vascular endothelial growth factor.

We further explored the inflammatory profile of participants who died early. There was substantial overlap between inflammatory mediators that were significantly different in early and late deaths versus survivors. However, the magnitude of differences between early deaths and survivors was much larger (Fig 9A). A Venn diagram including the inflammatory mediators that had statistically significant higher or lower values between each death group compared with survivors revealed two modules of uniquely different mediators, one in early deaths and one in late deaths (Fig 9B). The ability of these modules to predict early or late death was explored with a receiver operating characteristic (ROC) curve. Of note, modules significantly associated with early deaths had an area under the curve of 0.86 (*p* < 0.001) to distinguish early deaths from survivors, whereas the discrimination accuracy for modules significantly associated with late deaths was weak to distinguish late deaths from survivors (Fig 9C). We also conducted exploratory networks analysis of the soluble inflammatory mediators and detected three main nodes that had high numbers of strong correlations in early and late deaths. Participants who died early showed higher numbers of strong correlations between mediators. TNF-α and IL-4 had the highest numbers of strong correlations in each outcome group (S6 Fig).

## Discussion

We enrolled 576 hospitalized patients with HIV and newly diagnosed tuberculosis at presentation to hospital, collected samples at baseline, and followed patients for 12 weeks to ascertain vital status. We performed comprehensive tuberculosis investigations, measured host soluble inflammatory mediators, and compared patients who died with those who survived. We found high mortality (21.5%) despite timely initiation of antituberculous therapy. Clinician-attributed causes of death identified tuberculosis as the major contributor or one of the major contributors to death in 89.5% of cases. We observed disseminated tuberculosis in 64.6%, which was associated with mortality. One third of participants (33.9%) presented with features of sepsis syndrome, as indicated by elevated lactate, and amongst the patients in whom the clinician-attributed cause of death included tuberculosis as a major cause, 15.3% had rifampicin-resistant tuberculosis. We describe an immune profile identified by non-supervised hierarchical cluster analysis and principal components analysis that was associated with mortality in Cox proportional hazards analysis and was also associated with a higher tuberculosis dissemination score. The immune profile was dominated by soluble inflammatory mediators associated with the innate immune system and chemotactic signaling.

Tuberculosis is the leading cause of hospital admissions and in-hospital deaths in individuals with HIV in sub-Saharan Africa [2]. In our study, 576 hospitalized adults with HIV and a new diagnosis of tuberculosis were enrolled, and, like other studies [4–7], we observed a high case fatality rate of 21.5%. While 37.1% of deaths occurred within 7 days of enrolment, deaths continued to occur throughout the 12-week follow-up period.

We systematically assessed potential determinants of mortality, including the presence of rifampicin-resistant tuberculosis, time to initiation of antituberculosis therapy, concomitant infections, and disseminated tuberculosis. Tuberculosis was considered to be the major (or one of the major) contributors to death in the majority of cases, and 15.3% with tuberculosis-related deaths had rifampicin-resistant tuberculosis. A higher proportion of participants who died had CMV viremia, but this was not significant after adjustment for potential confounders. Ceftriaxone is the antibiotic recommended locally for patients with HIV with community-acquired pneumonia or suspected gram-negative infections. The majority of the cohort received ceftriaxone during the index admission. This was usually initiated in the emergency room, and 14% received ceftriaxone prior to arrival at hospital. There was, therefore, minimal delay in antibiotic initiation in patients with suspected sepsis. Infections with other opportunistic pathogens such as cryptococcosis and bacterial coinfections were not identified frequently.

Considering the low prevalence of bacterial coinfections identified and high numbers of very early deaths, it is possible that undiagnosed bacterial coinfections could have contributed to early deaths as well as the immune profiles that were associated with mortality. To address this, we performed a sensitivity analysis excluding patients who died within 7 days; in this sensitivity analysis, the association between the immune profiles and death were very similar to the main findings, suggesting that undiagnosed bacterial infections were not likely to be a major contributor to the immune profiles. This will need to be confirmed in future studies.

Similar to autopsy studies [14] and previous cohort studies [19, 20], we observed a high frequency (62.6%) of participants with disseminated tuberculosis, which was associated with mortality. Many participants presented with sepsis syndrome: 33.9% had elevated lactate and 34.7% had renal impairment, and these abnormalities were associated with death. In Africa, MTB BSI is found frequently amongst patients with HIV admitted with febrile illness (7.6%) [17], HIV-associated tuberculosis (31%) [18], and hypotensive sepsis syndrome (23%) [16,19]. Closely associated with MTB BSI is the detection of a mycobacterial cell wall component LAM in urine. Urine LAM positivity is associated with the presence of renal tuberculosis on autopsy [34], the presence of mycobacteria in the urine [35], and with positive tuberculosis blood culture [18,36,37]. A positive urine LAM test can therefore be regarded as a marker of disseminated tuberculosis even though the mechanism has not been clearly elucidated [38]. Xpert MTB/RIF detects *M*. *tuberculosis* DNA and thus indicates disseminated disease when positive in urine. A combination of urine Xpert and LAM tests can rapidly detect patients with MTB BSI [18], but high mortality persists despite implementation of these diagnostics [25,26]. In our study, the degree of dissemination of tuberculosis was quantified with a simple dissemination score (previously described [18]) that relies only on non-sputum samples, and a higher dissemination score was associated with mortality.

Principal components analysis described two principal components that were each weighted by functionally distinct groups of soluble inflammatory mediators. Principal component 1 was dominated by mediators associated with the innate immune system and chemotactic signaling, and this was associated with 2-fold higher mortality in a Cox proportional hazards model. A higher dissemination score was also associated with elevation of the innate immune mediators comprising principal component 1. In contrast, a second principal component, which contained mostly T-cell associated mediators, was associated with lower mortality in a Cox proportional hazards model, and higher values of T-cell associated mediators and growth factors were associated with a tuberculosis dissemination score of zero in hierarchical cluster analysis.

Possible mechanistic interpretations of our findings are that the higher levels of innate and chemotactic mediators reflect increased activation and recruitment of innate cells into the tissue in response to multi-organ infection. This may be an appropriate, but ultimately ineffective, response, with significant immunologic and metabolic costs to the host. Recruitment of innate cells to tissue may result in tissue damage and organ dysfunction. Monocyte and lymphocyte depletion could render patients more susceptible to secondary bacterial infections [39,40]. Hyperlactatemia may represent metabolic switching to aerobic glycolysis in immune cells to meet increased energy demands of the inflammatory response, at the cost of increased acidosis [41]. Hepcidin production and iron sequestration may limit extracellular bacillary growth at the cost of severe anemia [42]. Thus, the innate response may be initially protective, but it fails and potentially becomes harmful to the host due to overwhelming infection. From our data, it appears the major driver of mortality in these patients is disseminated tuberculosis itself, which triggers a pathophysiological process that results in death despite rapid initiation of standard antituberculosis therapy.

Our group and others have described suppressed or dysfunctional innate immune responses to bacterial stimuli, which were associated with mortality or clinical deterioration in critically ill participants with HIV-associated tuberculosis [43,44]. Both studies evaluated whole blood samples from HIV-associated tuberculosis participants, which were stimulated with bacterial antigens and heat-killed *M*. *tuberculosis*, and observed lower TNF-α production upon stimulation in participants with a poor outcome. This may indicate that innate immune cells in peripheral blood are already maximally stimulated in vivo.

We observed lower monocyte and lymphocyte counts in participants who died and significant negative correlations between these cell counts and IL-1Ra, IL-6, and IL-8.

Monocytes and lymphocytes are important sources of these mediators. These mediators are also secreted by several other cell types, which may account for the higher levels of mediators despite lower numbers of cells. Alternatively, despite lower cell numbers, the remaining monocytes and lymphocytes in participants who died may be producing increased amounts of these inflammatory mediators due to an enhanced inflammatory state. The higher levels of chemokines (IL-8, MIP-1β/CCL4, IP-10/CXCL10, and MIP-1α/CCL3) we observed in participants who died suggest that immune cells may be recruited to tissue and could be producing cytokines there, resulting in higher levels of innate mediators in blood.

We observed 3-fold higher IL-1Ra values in those who died compared with survivors but a striking 8-fold higher value in early deaths compared with survivors, with no difference in IL-1β values. IL-1Ra is secreted primarily by monocytes and macrophages and antagonizes the proinflammatory cytokines IL-1α and IL-Iβ. IL-1Ra binds to the IL-1R1 receptor and inhibits IL-1 signaling, thereby regulating inflammatory responses [45]. IL-1β is one of the key cytokines involved in the initial inflammatory response to *M*. *tuberculosis* infection, and IL-1Ra down-regulates and limits this immune response. IL-1Ra levels are elevated during *M*. *tuberculosis* infection, and gene polymorphisms of IL-1Ra have been associated with disease expression in tuberculosis [46] and mortality in meningococcal sepsis [47]. IL-1Ra is induced by HIV infection [48]. This finding may reflect an important anti-inflammatory signaling pathway, which debilitates the immune response to tuberculosis and predisposes patients to disseminated tuberculosis and death. This is a hypothesis that should be explored in future mechanistic studies.

Immune activation at baseline [11,49] and failure to resolve or increased immune activation after ART initiation have been described as risk factors for mortality in HIV-associated tuberculosis cohorts [12, 13, 50]. One of these studies was an outpatient cohort study that followed participants with HIV-associated tuberculosis and showed that higher pre-ART levels of MCP-1/CCL2, eotaxin, IL-10, TNF-α, and IL-6 were associated with 6-month mortality. It was also shown that a significant increase in IL-1Ra, IFNγ, and G-CSF concentrations at 4 weeks after ART initiation was associated with mortality [12]. The same group showed the presence of a single nucleotide polymorphism (SNP) involved in the inflammasome pathway, NOD-like receptor pyrin containing-3 (NLRP3) rs10754558-G, was independently associated with 6-month mortality, and variations in the genotype at NLRP3 rs10754558 influenced participants’ systemic inflammatory state pre-ART and at 4 weeks after ART initiation. The presence of this SNP appears to modulate inflammasome activation and contribute to increased inflammation [51], which may indicate a genetic predisposition to exaggerated inflammatory responses in some participants and portend a worse outcome.

Our study has several limitations. First, samples were obtained only at a single time point, at time of enrolment, and there was no longitudinal sampling. Second, we may have underestimated the role of bacterial infections on mortality. Bacterial blood cultures had a low yield, as many participants were given intravenous antibiotics before enrolment. The contribution of bacterial infections to deaths that occurred after discharge could not be ascertained. Third, there were no objective measures of adherence to antituberculosis therapy after discharge from hospital. We did not approach the analysis with training and validation subsets but used the entire data set to characterize biomarker profiles that can be tested and validated in future studies. Finally, we do not have autopsy information on causes of death.

Strengths of the study are that it is a large cohort, with extensive tuberculosis diagnostic testing and prospective follow-up and vital status known at 12 weeks for over 98% of participants. There was systematic ascertainment of other infections and contributors to mortality. Detailed clinical, laboratory, and immunologic assays and analyses provide insight into functional responses, unlike autopsy studies. We enrolled acutely ill patients with a decreased level of consciousness, which ensures that our results are generalizable to this vulnerable group.

### Future research

Our findings provide a rationale to consider novel strategies such as host-directed therapies and higher-dose rifampicin in this patient population. Rifampicin efficacy is exposure dependent [52], and current treatment doses (10 mg/kg/day) are at the lower end of the dose-response curve. This dose may be insufficient, particularly in acutely ill patients with disseminated tuberculosis and high mortality risk. Rifampicin doses up to 35 mg/kg/day [53] have been evaluated in patients with pulmonary tuberculosis, but high-dose rifampicin should also be evaluated in patients with disseminated HIV-associated tuberculosis, in whom safety considerations may be different. Additional strategies to rapidly lower the mycobacterial load, such as the use of fluoroquinolones with excellent early bactericidal activity, should be investigated in this patient group.

We postulate that the innate immune profile is driven by a high disseminated mycobacterial load and contributes to mortality. This provides a rationale to test host-directed therapy that could modulate this innate immune response in addition to more intensive antimicrobial therapies strategies. Adjunctive corticosteroids reduce mortality in adults with severe pneumonia and sepsis [54,55], and adjunctive recombinant IL-7 is well tolerated in sepsis and results in more rapid and sustained recovery of sepsis-induced lymphopenia [56]. Our findings of elevated inflammatory and innate immune mediators together with lower lymphocytes and lymphocyte-associated mediators in participants who died provide a rationale to evaluate one or both these strategies. Additionally, our finding that an immune profile of lower T-cell associated markers is associated with mortality provides a rationale to consider immediate ART, started on the same day as antituberculosis therapy, together with corticosteroids. The corticosteroids could reduce the risk of paradoxical tuberculosis immune reconstitution inflammatory syndrome [57] and modulate the innate immune response. However, neither ART status nor HIV viral load were associated with mortality in this cohort, and this suggests that immediate treatment of HIV may not alter short-term outcomes. Pharmacokinetic (PK) studies of antituberculosis drugs should include hospitalized acutely ill patients with HIV-associated tuberculosis at early therapeutic time points. We performed intensive PK sampling to measure concentrations of rifampicin, isoniazid, and pyrazinamide in a subset of this cohort, and these findings will be reported in a subsequent manuscript.

The nesting of pathogenesis studies within such intervention trials would afford the opportunity to better define causal pathophysiological relationships.

### Conclusions

In conclusion, high mortality in hospitalized patients with HIV associated tuberculosis is a critical public health problem requiring improved acute management strategies. In our study, disseminated tuberculosis (quantified by a 3-point dissemination score), features of sepsis syndrome, and rifampicin-resistant tuberculosis were associated with mortality. An immune profile dominated by elevated mediators of the innate immune system and chemotactic signaling was associated with mortality and a higher dissemination score. Even though causal relationships cannot be established from this study, the findings that an innate immune profile associates with both mortality and tuberculosis dissemination provide important insights into pathophysiological processes. These findings provide a rationale to evaluate immunomodulatory therapies and more rapidly bactericidal antituberculosis treatment strategies in future studies.

## Supporting information

S1 Table Criteria used to classify participants with probable tuberculosis. Participants who did not have microbiologically confirmed tuberculosis were assessed for features compatible with tuberculosis and classified as probable tuberculosis, possible tuberculosis, or no tuberculosis (see also S2 Table). Participants with probable tuberculosis were included in the analysis along with participants with microbiologically confirmed tuberculosis [58].
(DOCX)

S2 Table Exclusions: Details of participants with no tuberculosis and possible tuberculosis. Participants without microbiologically confirmed tuberculosis were assessed for features compatible with tuberculosis and classified as probable tuberculosis, possible tuberculosis, or no tuberculosis (see also S1 Table). Participants with possible and no tuberculosis were excluded from analysis.
(DOCX)

S3 Table Cox proportional hazards analysis evaluating the association of CMV viremia with mortality. The relationship between CMV infection and outcome may be confounded by HIV-related factors and immunosuppression. A Cox proportional hazards analysis was performed, and each variable was evaluated individually (unadjusted) and then in a multivariate model including age, sex, HIV viral load, and CD4 count to adjust for patient-specific variance and HIV-related factors. The model was censored at 28 days to meet the proportional hazards assumption, and the global proportional hazards test for the multivariable model result was *p* = 0.75. CD4, cluster of differentiation 4; CMV, cytomegalovirus.
(DOCX)

S4 Table Host soluble mediators of inflammation values in hospitalized HIV-TB coinfected participants: Comparison between early deaths (within 7 days after enrolment) and survivors. Host soluble inflammatory mediators were measured in a random selection of participants with HIV-associated tuberculosis (*n* = 46 early deaths and *n* = 391 survivors) using the Biorad Bioplex 200 Luminex platform. Fluorescence index values are presented.*TGF-β1 concentrations were measured with ELISA and is presented in picograms per milliliter. This table shows differences between early deaths and survivors. Inflammatory mediators are arranged into three groups: mediators that were higher in early deaths, mediators that were lower in early deaths, and mediators that showed no difference between survival groups. Each group is ranked from lowest to highest *p*-values. Comparisons between early deaths and survivors were made using the Wilcoxon rank sum test. The *p*-values were corrected for multiple comparisons with Holms-Bonferroni correction. Bold *p*-values indicate mediators that remained significantly different after correction for multiple comparisons. CCL, C-C motif chemokine ligand; CSF2, colony stimulating factor 2; CSF3, colony stimulating factor 3; CXCL, C-X-C motif chemokine ligand; FGF, basic fibroblast growth factor; G-CSF, granulocyte-colony stimulating factor; GM-CSF, granulocyte-macrophage colony-stimulating factor; IFNγ, interferon gamma; IL, interleukin; IP-10, interferon gamma-induced protein; MCP, monocyte chemoattractant protein; MIP, macrophage inflammatory protein; PDGF, platelet-derived growth factor; Ra, receptor antagonist; RANTES, regulated on activation, normal T-cell expressed and secreted; TGF-β1, transforming growth factor beta 1; TNFα, tumor necrosis factor alpha; VEGF, vascular endothelial growth factor.
(DOCX)

S5 Table Hospitalized patients with HIV-associated tuberculosis: Bacterial culture results from urine, sputum, stool, and other anatomical sites. Results for all bacterial cultures that were performed in hospital were captured. The study team performed sputum bacterial cultures on patients when sufficient sputum was obtained to perform tuberculosis tests and bacterial culture. All other tests were performed in routine service by the medical teams, as clinically indicated. Results presented as *n* (%). The Fisher exact test was used to compare proportions. *n* = 105 patients had urine bacterial culture performed. *n* = 312 patients had sputum bacterial culture performed. *n* = 27 patients had pleural fluid bacterial culture performed; no sample had a positive bacterial culture. *n* = 154 patients had CSF bacterial culture performed; three patients had a positive culture. One patient with clinical tuberculosis cultured *Neisseria meningitidis* in CSF and survived. One patient with microbiologically proven TB cultured *Bacillus* species in CSF and survived. One patient with microbiologically proven TB cultured *Pseudomonas putida* in CSF and died. CSF, cerebrospinal fluid; TB, tuberculosis.
(DOCX)

S1 Fig Kaplan Meier survival curves stratified by lactate level and tuberculosis dissemination score and parameters used to calculate dissemination score. Kaplan Meier survival curves representing percentage survival in participants with lactate above the upper limit of normal (in red) to those with normal lactate (in blue). Kaplan Meier survival curves representing percentage survival in participants with a tuberculosis dissemination score of 0–3. A score was allocated to participants who had valid results for all three tests used to calculate the tuberculosis dissemination score (urine LAM assay, urine Xpert MTB/RIF assay, mycobacterial blood culture) and known outcome at week 12 (*n* = 457, *n* = 93 deaths and *n* = 364 survivors). Kaplan Meier survival curves representing percentage survival in patients who tested positive for only TB blood culture, those who tested positive for both urine Xpert and urine LAM, those who tested positive for only one of the two urine tests, and those who did not test positive for any of the markers used to calculate the dissemination score. Curves were compared using log-rank (Mantel-Cox) test. LAM, lipoarabinomannan; TB, tuberculosis.
(TIF)

S2 Fig Kaplan Meier survival curves for host soluble inflammatory mediators that were significantly increased in participants who died. Kaplan Meier survival curves showing percentage survival over time. All soluble inflammatory mediators that were significantly increased in participants who died are shown in this figure. Participants were stratified by those with values above or below the median fluorescence index values for each mediator. Curves were compared using log-rank (Mantel-Cox) test.
(TIF)

S3 Fig Kaplan Meier survival curves for host soluble inflammatory mediators that were significantly lower in participants who died. Kaplan Meier survival curves showing percentage survival over time. All soluble inflammatory mediators that were significantly lower in participants who died are shown in this figure. Participants were stratified by those with values above or below the median fluorescence index values for each mediator. Curves were compared using log-rank (Mantel-Cox) test.
(TIF)

S4 Fig Kaplan Meier survival curves: Neutrophil count, monocyte count, and lymphocyte count. Kaplan Meier survival curves showing percentage survival over time. Participants were stratified by those with cell counts above or below the median values for each type of cell. Curves were compared using log-rank (Mantel-Cox) test.
(TIF)

S5 Fig Association of PC2 score with absolute lymphocyte count and CD4 T-cell count. We explored the association of PC2 with lymphocyte count and CD4 cell count. PC2 and lymphocytes had a very weak positive correlation and PC2 and CD4 cell count were not correlated. CD4, cluster of differentiation 4; PC2, principal component 2.
(TIF)

S6 Fig Network analysis of host soluble inflammatory mediators. **(A)** Profiles of correlations between inflammatory mediators in different clinical groups were examined using network analysis of the Spearman correlation matrices. Networks represent strong Spearman correlations (*p* < 0.001; Spearman rank value >0.7 or <−0.7). Mediators were clustered based on a similarity index of the correlation profiles using a modularity algorithm and depicted with Fruchterman Reingold (force-directed graph drawing). Using this approach, three main nodes were detected. Both cytokines and cells counts were included. Only mediators that had strong correlations were plotted, to reduce visual pollution. **(B)** Node analysis was used to illustrate the number of strong correlations per mediator. Mediators were grouped according to the number of connections using hierarchical clustering (Ward method).
(TIF)

S1 Document The original prospective protocol, including the analysis plan for this study. Ethical approval was obtained in 2013. Recruitment took place from 2014 to 2016, and laboratory work was conducted in 2017. Analysis was conducted in 2018.
(PDF)

S1 STROBE checklist STROBE checklist completed, with section and paragraph details of where relevant information on the STROBE checklist can be found in the manuscript. (DOCX)

## Abbreviations

aHR: adjusted hazard ratio
ART: antiretroviral therapy
CCL: C-C motif chemokine ligand
CD4: cluster of differentiation 4
CMV: cytomegalovirus
CRAG: cryptococcal antigen
CSF2: colony stimulating factor 2
CSF3: colony stimulating factor 3
CXCL: C-X-C motif chemokine ligand
ELISA: enzyme-linked immunosorbent assay
FGF: basic fibroblast growth factor
G-CSF: granulocyte colony stimulating factor
GM-CSF: granulocyte-macrophage colony stimulating factor
IFN-γ: interferon gamma
IL: interleukin
IL-1Ra: IL-1 receptor antagonist
IP-10: interferon gamma-induced protein
LAM: lipoarabinomannan
MCP: monocyte chemoattractant protein
MIP: macrophage inflammatory protein
MTB BSI: *Mycobacterium tuberculosis* bloodstream infection
NLRP3: NOD-like receptor pyrin containing-3
NHLS: National Health Laboratory Services
PC: principal component
PDGF: platelet-derived growth factor
PJP: *Pneumocystis jirovecii* pneumonia
PK: pharmacokinetic
RANTES: regulated on activation, normal T-cell expressed and secreted
ROC: receiver operating characteristic
SNP: single nucleotide polymorphism
SOFA: sequential organ failure assessment score
TGF-β1: transforming growth factor beta 1
TNF-α: tumor necrosis factor-alpha
VEGF: vascular endothelial growth factor
