# Supplementary material for: Clinical, microbiologic, and immunologic determinants of mortality in hospitalized patients with HIV-associated tuberculosis: A prospective cohort study
Source: PLoS Med. 2019 Jul 5;16(7):e1002840. doi: 10.1371/journal.pmed.1002840 (PMC6611568; doi:10.1371/journal.pmed.1002840)
Supplement: S1 Table — Participants who did not have microbiologically confirmed tuberculosis were assessed for features compatible with tuberculosis and classified as probable tuberculosis, possible tuberculosis, or no tuberculosis (see also S2 Table). Participants with probable tuberculosis were included in the analysis along with participants with microbiologically confirmed tuberculosis [58]. (DOCX) [file pmed.1002840.s001.docx]

**S1 Table: Criteria used to classify participants with probable tuberculosis**

| **Probable tuberculosis** | **Number** |
| --- | --- |
| Tuberculosis not microbiologically confirmed and urine LAM positive | 18 |
| Tuberculosis not microbiologically confirmed; urine LAM negative; pleural effusion which was treated for tuberculosis or exudative pleural effusion with adenosine deaminase >30g/dL and no alternative diagnosis made | 21 |
| Tuberculosis not microbiologically confirmed; urine LAM negative; pericardial effusion treated for tuberculosis and no alternative diagnosis made | 3 |
| Tuberculosis not microbiologically confirmed; urine LAM negative; miliary tuberculosis on chest X-ray treated for tuberculosis and no alternative diagnosis made | 6 |
| Tuberculosis not microbiologically confirmed; urine LAM negative; features of tuberculosis on abdominal ultrasound (multiregion nodes ≥ 1 cm diameter or splenic microabcesses) treated for tuberculosis and no alternative diagnosis made | 3 |
| Tuberculosis not microbiologically confirmed; urine LAM negative; cerebrospinal fluid (CSF) picture compatible with probable tuberculous meningitis (TBM) with CSF score ≥2 out of 4 [1]), treated for TBM and no alternative diagnosis made. | 9 |
| Tuberculosis not microbiologically confirmed; urine LAM negative; computed tomography (CT) scan features of central nervous system tuberculosis, treated for tuberculosis with no alternative diagnosis made | 1 |
| Tuberculosis not microbiologically confirmed; urine LAM negative; compatible chest X-ray, treated for tuberculosis and remained on treatment | 28 |
| Total | 89 |

**S1 Table:** Patients who did not have microbiologically confirmed tuberculosis were assessed for features compatible with tuberculosis and classified as probable tuberculosis, possible tuberculosis or no tuberculosis (see also Supplementary Table 2). Participants with probable tuberculosis were included in the analysis along with participants with microbiologically confirmed tuberculosis.

1. Marais S, Thwaites G, Schoeman JF, et al. Tuberculous meningitis: a uniform case definition for use in clinical research. Lancet Infect Dis **2010**; 10(11): 803-12.
