# Supplementary material for: Clinical, microbiologic, and immunologic determinants of mortality in hospitalized patients with HIV-associated tuberculosis: A prospective cohort study
Source: PLoS Med. 2019 Jul 5;16(7):e1002840. doi: 10.1371/journal.pmed.1002840 (PMC6611568; doi:10.1371/journal.pmed.1002840)
Supplement: S2 Table — Participants without microbiologically confirmed tuberculosis were assessed for features compatible with tuberculosis and classified as probable tuberculosis, possible tuberculosis, or no tuberculosis (see also S1 Table). Participants with possible and no tuberculosis were excluded from analysis. (DOCX) [file pmed.1002840.s002.docx]

**S2 Table: Exclusions: Details of participants with no tuberculosis and possible tuberculosis:**

| **No tuberculosis** | **Number** |
| --- | --- |
| Tuberculosis not microbiologically confirmed; urine LAM negative and disseminated *cryptococcosis* | 6 |
| Tuberculosis not microbiologically confirmed; urine LAM negative and culture proven bacterial bloodstream infection | 9 |
| Tuberculosis not microbiologically confirmed; urine LAM negative and clinical diagnosis of community acquired pneumonia with no criteria for clinical tuberculosis and improved without antituberculosis treatment | 33 |
| Tuberculosis not microbiologically confirmed; urine LAM negative and treated for *Pneumocystis jiroveci* pneumonia, not treated for tuberculosis and improved or *P. jiroveci* diagnosed on post mortem | 6 |
| Tuberculosis not microbiologically confirmed; urine LAM negative and no criteria for clinical tuberculosis with alternative diagnosis confirmed (examples, malignancy or venous thromboembolism) | 15 |
| **Possible tuberculosis** |  |
| Tuberculosis not microbiologically confirmed; urine LAM negative and clinical suspicion of tuberculosis together with or indistinguishable from a second infection; neither infection proven; treated for both. | 14 |

**S2 Table:** Patients without microbiologically confirmed tuberculosis were assessed for features compatible with tuberculosis and classified as probable tuberculosis, possible tuberculosis or no tuberculosis (see also S1 Table). Participants with possible and no tuberculosis were excluded from analysis.
