# Supplementary material for: Clinical, microbiologic, and immunologic determinants of mortality in hospitalized patients with HIV-associated tuberculosis: A prospective cohort study
Source: PLoS Med. 2019 Jul 5;16(7):e1002840. doi: 10.1371/journal.pmed.1002840 (PMC6611568; doi:10.1371/journal.pmed.1002840)
Supplement: S3 Table — The relationship between CMV infection and outcome may be confounded by HIV-related factors and immunosuppression. A Cox proportional hazards analysis was performed, and each variable was evaluated individually (unadjusted) and then in a multivariate model including age, sex, HIV viral load, and CD4 count to adjust for patient-specific variance and HIV-related factors. The model was censored at 28 days to meet the proportional hazards assumption, and the global proportional hazards test for the multivariable model result was p = 0.75. CD4, cluster of differentiation 4; CMV, cytomegalovirus. (DOCX) [file pmed.1002840.s003.docx]

**S3 Table: Cox proportional hazards analysis evaluating association of cytomegalovirus viraemia with mortality:**

| **Variable** | **Hazard Ratio (95% CI)** | **p** | **Adjusted Hazard Ratio (95% CI)** | **p** |
| --- | --- | --- | --- | --- |
| CMV viral load detectable | 1.50 (0.95-2.30) | 0.081 | 1.37 (0.87-2.17) | 0.171 |
| Age  (per 5-year increase) | 1.30 (1.10-1.40) | <0.001 | 1.30 (1.17-1.44) | <0.001 |
| Male sex | 1.00 (0.65-1.60) | 0.980 | 0.94 (0.60-1.48) | 0.790 |
| HIV Viral Load  (per 1000 copies/ml increase) | 1.00 (1.00-1.00) | 0.370 | 1.00 (1.00-1.00) | 0.310 |
| CD4 count  (per 10 cells increase) | 0.96 (0.93-0.99) | 0.014 | 0.96 (0.93-0.99) | 0.014 |

**S3 Table:** The relationship between cytomegalovirus infection and outcome may be confounded by HIV related factors and immunosuppression. A Cox proportional hazards analysis was performed and each variable was evaluated individually (unadjusted) and then in a multivariate model including for age, sex, HIV viral load and CD4 count to adjust for patient specific variance and HIV-related factors. The model was censored at 28 days to meet the proportional hazards assumption and the global proportional hazards test for the multivariable model result was p=0.75.
