# Supplementary material for: Clinical, microbiologic, and immunologic determinants of mortality in hospitalized patients with HIV-associated tuberculosis: A prospective cohort study
Source: PLoS Med. 2019 Jul 5;16(7):e1002840. doi: 10.1371/journal.pmed.1002840 (PMC6611568; doi:10.1371/journal.pmed.1002840)
Supplement: S4 Table — Host soluble inflammatory mediators were measured in a random selection of participants with HIV-associated tuberculosis (n = 46 early deaths and n = 391 survivors) using the Biorad Bioplex 200 Luminex platform. Fluorescence index values are presented.*TGF-β1 concentrations were measured with ELISA and is presented in picograms per milliliter. This table shows differences between early deaths and survivors. Inflammatory mediators are arranged into three groups: mediators that were higher in early deaths, mediators that were lower in early deaths, and mediators that showed no difference between survival groups. Each group is ranked from lowest to highest p-values. Comparisons between early deaths and survivors were made using the Wilcoxon rank sum test. The p-values were corrected for multiple comparisons with Holms-Bonferroni correction. Bold p-values indicate mediators that remained significantly different after correction for multiple comparisons. CCL, C-C motif chemokine ligand; CSF2, colony stimulating factor 2; CSF3, colony stimulating factor 3; CXCL, C-X-C motif chemokine ligand; FGF, basic fibroblast growth factor; G-CSF, granulocyte-colony stimulating factor; GM-CSF, granulocyte-macrophage colony-stimulating factor; IFNγ, interferon gamma; IL, interleukin; IP-10, interferon gamma-induced protein; MCP, monocyte chemoattractant protein; MIP, macrophage inflammatory protein; PDGF, platelet-derived growth factor; Ra, receptor antagonist; RANTES, regulated on activation, normal T-cell expressed and secreted; TGF-β1, transforming growth factor beta 1; TNFα, tumor necrosis factor alpha; VEGF, vascular endothelial growth factor. (DOCX) [file pmed.1002840.s004.docx]

**S4 Table: Host soluble mediators of inflammation values in hospitalized HIV-TB co-infected participants: Comparison between early deaths (within 7 days after enrolment) and survivors**

|  | **Early Deaths** | **Survivors** | **p** | **Holms-Bonferroni p** |
| --- | --- | --- | --- | --- |
|  | **n=35** | **n=391** |  |  |
| **Higher in participants who died early** | | | | |
| IL-8 | 428.5 [195.5, 1197.8] | 110.0 [78.5, 165.5] | <0.001 | **<0.001** |
| IL-1Ra | 1417.0 [449.8, 3866.0] | 169.5 [93.0, 397.5] | <0.001 | **<0.001** |
| MIP-1β/CCL4 | 1809.0 [917.0, 4051.3] | 624.5 [397.5, 1087.5] | <0.001 | **<0.001** |
| IP-10/CXCL10 | 16911.0 [8412.5, 22836.3] | 6495.0 [3301.5, 11846.3] | <0.001 | **<0.001** |
| IL-6 | 590.0 [244.0, 1092.3] | 208.0 [119.3, 359.8] | <0.001 | **<0.001** |
| RANTES/CCL5 | 10728.5 [4928.8, 13834.0] | 15369.5 [12732.5, 16552.3] | <0.001 | **<0.001** |
| MIP-1α/CCL3 | 248.0 [92.3, 462.0] | 93.0 [65.8, 156.3] | <0.001 | **<0.001** |
| MCP-1/CCL2 | 153.0 [87.0, 453.0] | 95.5 [75.0, 138.0] | <0.001 | **0.012** |
| IL-9 | 189.0 [146.0, 435.5] | 153.0 [121.0, 205.0] | 0.001 | **0.029** |
| **Lower in participants who died early** | | | | |
| IL-13 | 24.0 [17.5, 32.5] | 39.0 [29.0, 59.5] | <0.001 | **<0.001** |
| PDGF | 72.0 [55.5, 133.3] | 201.0 [84.0, 418.5] | <0.001 | **<0.001** |
| IL-5 | 18.0 [13.5, 25.0] | 31.0 [22.0, 43.5] | <0.001 | **<0.001** |
| IL-7 | 26.0 [22.0, 31.8] | 35.0 [28.0, 45.3] | <0.001 | **0.004** |
| IL-12p70 | 43.5 [34.5, 56.8] | 56.0 [42.0, 76.8] | 0.001 | **0.037** |
| **No difference between early deaths and survivors** | | | | |
| FGF | 44.0 [38.0, 57.0] | 54.0 [43.8, 69.0] | 0.004 | 0.118 |
| G-CSF/CSF3 | 91.0 [51.5, 218.3] | 67.0 [54.0, 90.5] | 0.015 | 0.407 |
| *TGF-β1 | 18.1 [14.2, 32.4] | 26.4 [15.7, 53.9] | 0.020 | 0.571 |
| IL-4 | 41.0 [28.5, 57.8] | 48.0 [36.8, 63.3] | 0.059 | 1.000 |
| IL-15 | 104.0 [75.0, 133.5] | 89.5 [74.0, 114.3] | 0.078 | 1.000 |
| IL-1β | 69.5 [57.3, 122.0] | 64.0 [50.0, 84.5] | 0.104 | 1.000 |
| IL-17 | 56.0 [42.5, 85.8] | 64.5 [48.8, 90.3] | 0.181 | 1.000 |
| IL-10 | 80.5 [53.5, 115.5] | 69.0 [55.0, 85.0] | 0.201 | 1.000 |
| VEGF | 114.5 [64.5, 150.5] | 107.0 [78.8, 158.8] | 0.446 | 1.000 |
| Eotaxin | 69.5 [45.0, 136.3] | 66.0 [53.0, 88.3] | 0.592 | 1.000 |
| IFNγ | 52.0 [33.5, 79.5] | 54.0 [39.0, 74.5] | 0.663 | 1.000 |
| GM-CSF/CSF2 | 88.0 [68.3, 116.3] | 89.5 [72.0, 113.0] | 0.895 | 1.000 |
| TNFα | 43.0 [31.5, 60.0] | 43.5 [36.0, 54.3] | 0.914 | 1.000 |
| IL-2 | 66.0 [50.8, 87.0] | 68.0 [55.3, 81.0] | 0.988 | 1.000 |

**S4 Table:** Host soluble inflammatory mediators were measured in a random selection of participants with HIV-associated tuberculosis (n= 35 early deaths and n=391 survivors) using the Biorad Bioplex 200 Luminex platform, except for transforming growth factor beta 1 (TGF-β1) concentrations which were measured with enzyme-linked immunosorbent assay (ELISA). This table shows differences between early deaths and survivors. Inflammatory mediators are arranged into three groups: Mediators which were higher in early deaths, mediators which were lower in early deaths and mediators which showed no difference between survival groups. Each group is ranked from lowest to highest p-values. Fluorescence index values are presented for all mediators except *TGF-β1 which is presented in picogram per millilitre. Comparisons between early deaths and survivors were made using the Wilcoxon rank sum test. P-values were corrected for multiple comparisons with Holms-Bonferroni correction. Bold p-values indicate mediators which remained significantly different after correction for multiple comparisons.

IL: interleukin, MIP: monocyte inflammatory protein, CCL: chemokine (C-C motif) ligand, Ra: receptor antagonist, IP-10: interferon gamma induced protein, CXCL: C-X-C motif chemokine, MIP: macrophage inflammatory protein, MCP: monocyte chemoattractant protein, RANTES: regulated on activation, normal T cell expressed and secreted, PDGF: platelet-derived growth factor , FGF: fibroblast growth factor, IFNγ: interferon gamma, TNFα: tumour necrosis factor alpha, GM-CSF: granulocyte-macrophage colony-stimulating factor, CSF2: colony stimulating factor 2, VEGF: vascular endothelial growth factor, G-CSF: granulocyte-colony stimulating factor, CSF3: colony stimulating factor 3
