# Supplementary material for: Clinical, microbiologic, and immunologic determinants of mortality in hospitalized patients with HIV-associated tuberculosis: A prospective cohort study
Source: PLoS Med. 2019 Jul 5;16(7):e1002840. doi: 10.1371/journal.pmed.1002840 (PMC6611568; doi:10.1371/journal.pmed.1002840)
Supplement: S5 Table — Results for all bacterial cultures that were performed in hospital were captured. The study team performed sputum bacterial cultures on patients when sufficient sputum was obtained to perform tuberculosis tests and bacterial culture. All other tests were performed in routine service by the medical teams, as clinically indicated. Results presented as n (%). The Fisher exact test was used to compare proportions. n = 105 patients had urine bacterial culture performed. n = 312 patients had sputum bacterial culture performed. n = 27 patients had pleural fluid bacterial culture performed; no sample had a positive bacterial culture. n = 154 patients had CSF bacterial culture performed; three patients had a positive culture. One patient with clinical tuberculosis cultured Neisseria meningitidis in CSF and survived. One patient with microbiologically proven TB cultured Bacillus species in CSF and survived. One patient with microbiologically proven TB cultured Pseudomonas putida in CSF and died. CSF, cerebrospinal fluid; TB, tuberculosis. (DOCX) [file pmed.1002840.s005.docx]

**S5 Table: Hospitalized patients with HIV-associated tuberculosis: Bacterial culture results from urine, sputum, stool and other anatomical sites**

|  |  | **Died** | **Survived** | **p** |
| --- | --- | --- | --- | --- |
|  |  | **n=124** | **n=443** |  |
| Urine bacterial culture | *Enterococcus* species | 2 (1.6) | 3 (0.7) | 0.924 |
|  | Gram negative organism | 2 (1.6) | 2 (0.5) |  |
|  | Yeast | 5 (4) | 9 (2) |  |
| Sputum bacterial culture | *Candida albicans* | 2 (1.6) | 6 (1.4) | 0.202 |
|  | Gram negative organism | 3 (2.4) | 3 (0.7) |  |
|  | *Moraxella catarrhalis* | 0 | 1 (0.2) |  |
|  | *Rhizopus* species | 0 | 1 (0.2) |  |
|  | *Staphylococcus aureus* | 0 | 9 (2.0) |  |
|  | Mixed oral flora | 7 (5.6) | 38 (8.6) |  |
| *Clostridium difficile* test in stool | Positive | 2 (1.6) | 6 (1.4) | 1.000 |

**S5 Table:** Results for all bacterial cultures which were performed in hospital were captured. The study team performed sputum bacterial cultures on patients when sufficient sputum was obtained to perform tuberculosis tests and bacterial culture. All other tests were performed in routine service by the medical teams as clinically indicated.

Results presented as n (%).

Fisher’s exact test used to compare proportions.

n= 105 patients had urine bacterial culture performed

n= 312 patients had sputum bacterial culture performed

n= 27 patients had pleural fluid bacterial culture performed: no sample had a positive bacterial culture

n= 154 patients had cerebrospinal fluid (CSF) bacterial culture performed: 3 patients had a positive culture:

One patient with clinical tuberculosis cultured *Neisseria meningitidis* in CSF and survived.

One patient with microbiologically proven TB cultured *Bacillus* species in CSF and survived.

One patient with microbiologically proven TB cultured *Pseudomonas putida* in CSF and died.
