# Supplementary figures and images for: Clinical, microbiologic, and immunologic determinants of mortality in hospitalized patients with HIV-associated tuberculosis: A prospective cohort study
Source: PLoS Med. 2019 Jul 5;16(7):e1002840. doi: 10.1371/journal.pmed.1002840 (PMC6611568; doi:10.1371/journal.pmed.1002840)

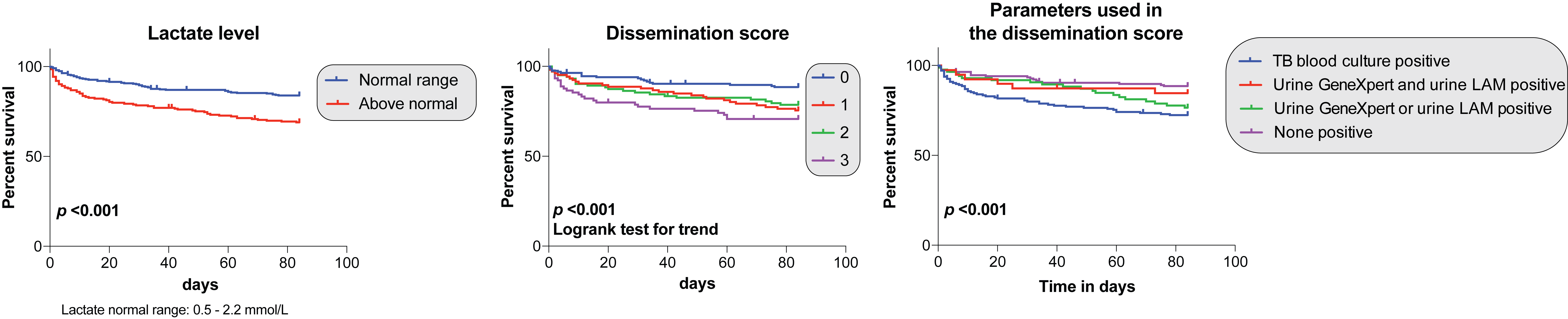

Supplement: S1 Fig — Kaplan Meier survival curves representing percentage survival in participants with lactate above the upper limit of normal (in red) to those with normal lactate (in blue). Kaplan Meier survival curves representing percentage survival in participants with a tuberculosis dissemination score of 0–3. A score was allocated to participants who had valid results for all three tests used to calculate the tuberculosis dissemination score (urine LAM assay, urine Xpert MTB/RIF assay, mycobacterial blood culture) and known outcome at week 12 (n = 457, n = 93 deaths and n = 364 survivors). Kaplan Meier survival curves representing percentage survival in patients who tested positive for only TB blood culture, those who tested positive for both urine Xpert and urine LAM, those who tested positive for only one of the two urine tests, and those who did not test positive for any of the markers used to calculate the dissemination score. Curves were compared using log-rank (Mantel-Cox) test. LAM, lipoarabinomannan; TB, tuberculosis. (TIF) [file pmed.1002840.s006.tif]

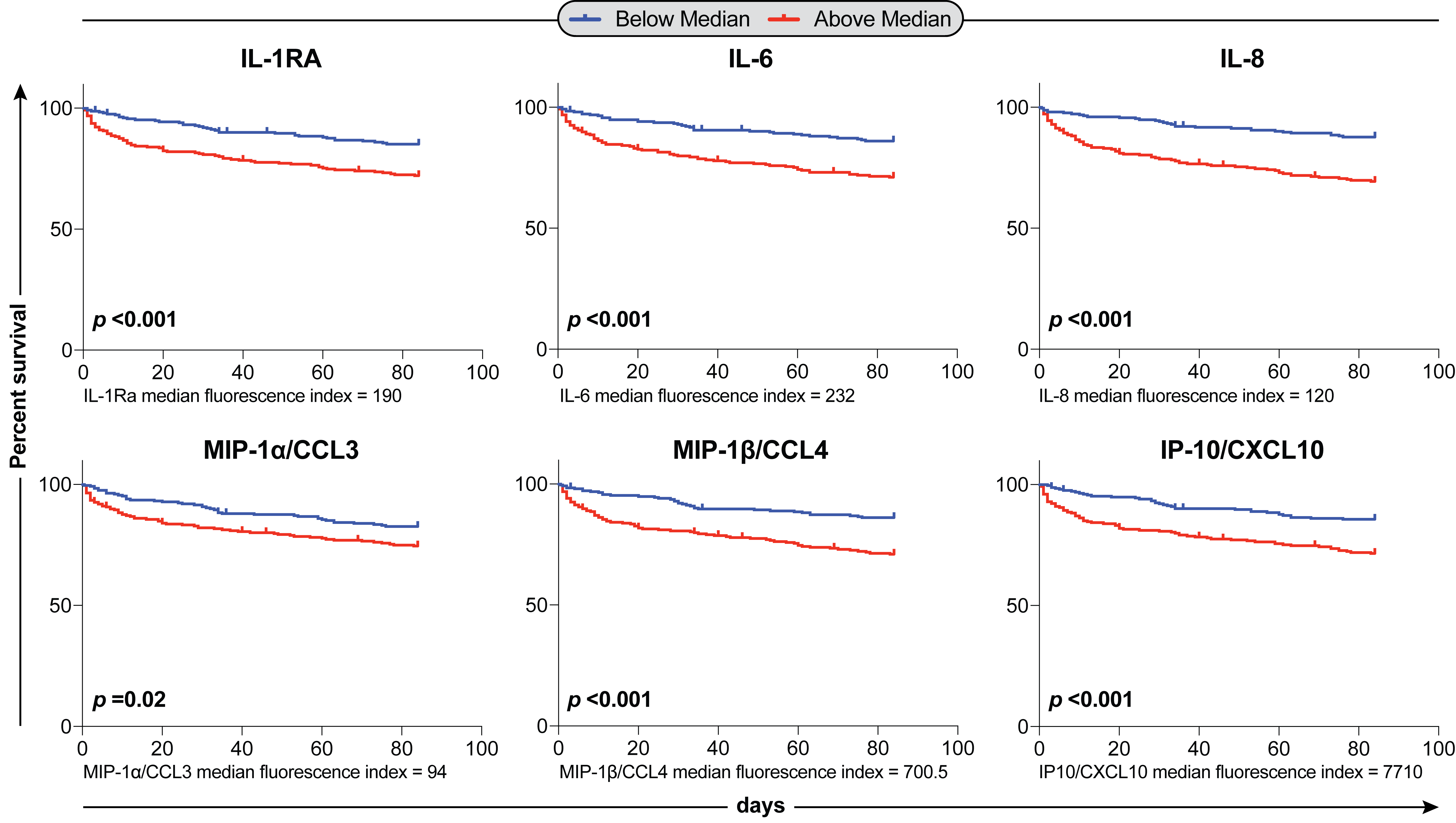

Supplement: S2 Fig — Kaplan Meier survival curves showing percentage survival over time. All soluble inflammatory mediators that were significantly increased in participants who died are shown in this figure. Participants were stratified by those with values above or below the median fluorescence index values for each mediator. Curves were compared using log-rank (Mantel-Cox) test. (TIF) [file pmed.1002840.s007.tif]

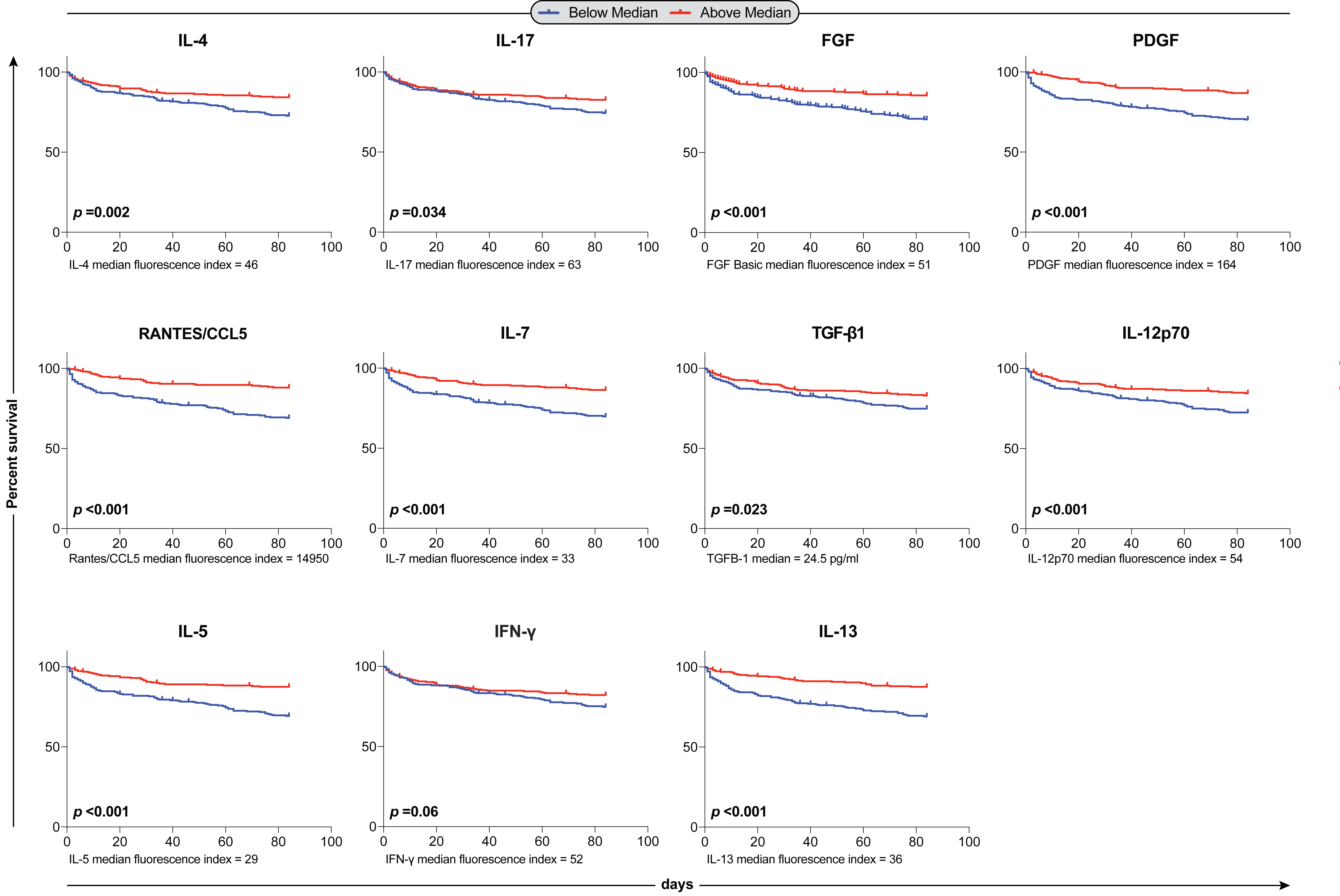

Supplement: S3 Fig — Kaplan Meier survival curves showing percentage survival over time. All soluble inflammatory mediators that were significantly lower in participants who died are shown in this figure. Participants were stratified by those with values above or below the median fluorescence index values for each mediator. Curves were compared using log-rank (Mantel-Cox) test. (TIF) [file pmed.1002840.s008.tif]

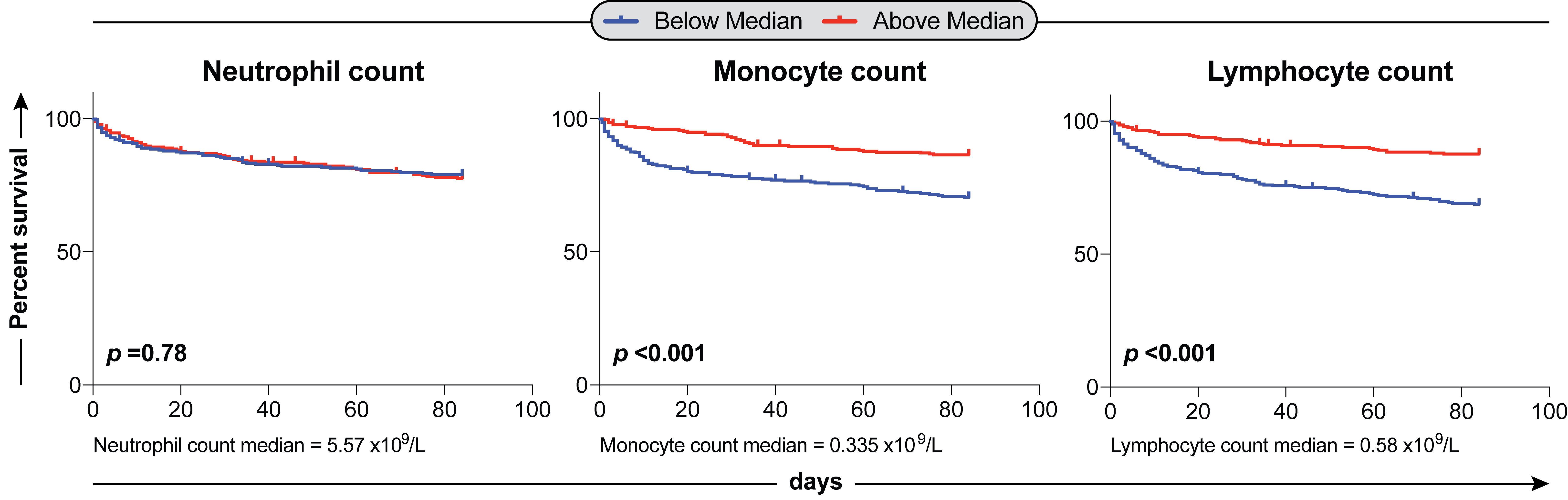

Supplement: S4 Fig — Kaplan Meier survival curves showing percentage survival over time. Participants were stratified by those with cell counts above or below the median values for each type of cell. Curves were compared using log-rank (Mantel-Cox) test. (TIF) [file pmed.1002840.s009.tif]

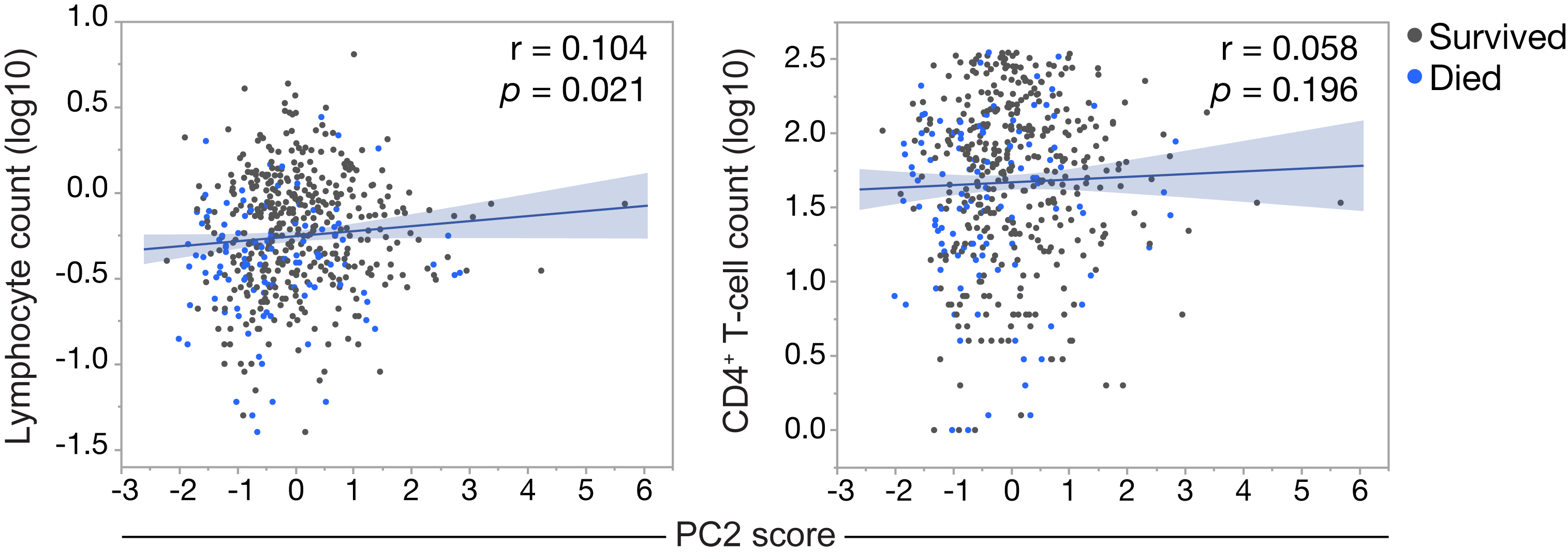

Supplement: S5 Fig — We explored the association of PC2 with lymphocyte count and CD4 cell count. PC2 and lymphocytes had a very weak positive correlation and PC2 and CD4 cell count were not correlated. CD4, cluster of differentiation 4; PC2, principal component 2. (TIF) [file pmed.1002840.s010.tif]

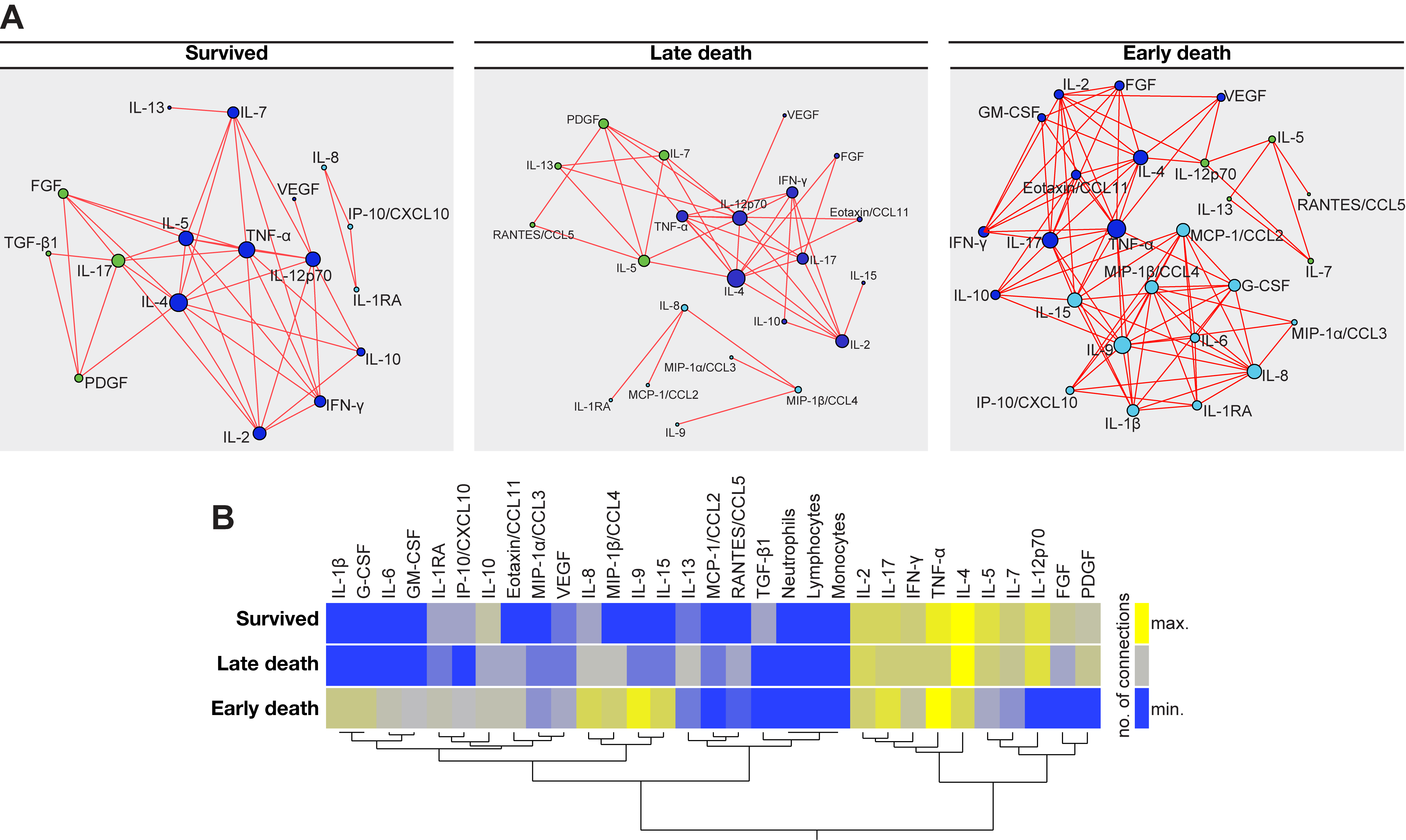

Supplement: S6 Fig — (A) Profiles of correlations between inflammatory mediators in different clinical groups were examined using network analysis of the Spearman correlation matrices. Networks represent strong Spearman correlations (p < 0.001; Spearman rank value >0.7 or <−0.7). Mediators were clustered based on a similarity index of the correlation profiles using a modularity algorithm and depicted with Fruchterman Reingold (force-directed graph drawing). Using this approach, three main nodes were detected. Both cytokines and cells counts were included. Only mediators that had strong correlations were plotted, to reduce visual pollution. (B) Node analysis was used to illustrate the number of strong correlations per mediator. Mediators were grouped according to the number of connections using hierarchical clustering (Ward method). (TIF) [file pmed.1002840.s011.tif]
