# Supplementary material for: Clinical, microbiologic, and immunologic determinants of mortality in hospitalized patients with HIV-associated tuberculosis: A prospective cohort study
Source: PLoS Med. 2019 Jul 5;16(7):e1002840. doi: 10.1371/journal.pmed.1002840 (PMC6611568; doi:10.1371/journal.pmed.1002840)
Supplement: S1 Document — Ethical approval was obtained in 2013. Recruitment took place from 2014 to 2016, and laboratory work was conducted in 2017. Analysis was conducted in 2018. (PDF) [file pmed.1002840.s012.pdf]

DEFINING INTERVENTIONS TO REDUCE MORTALITY IN SEVERE HIV-ASSOCIATED TUBERCULOSIS

PROTOCOL

Prospective observational cohort study
Short title: KDH-TB Study

Principal investigator:	Associate Professor Graeme Meintjes
Affiliation:	Institute of Infectious Disease and Molecular Medicine, Department of Medicine, University of Cape Town
Contact details:	
Co-investigators:	Associate Professor Andrew Boulle<br>Dr Rosie Burton<br>Dr Craig Corcoran<br>Professor Gary Maartens<br>Associate Professor Helen McIlleron<br>Professor Mark Nicol<br>Dr Charlotte Schutz<br>Associate Professor Helen Wainwright<br>Associate Professor Katalin Wilkinson<br>Professor Robert J. Wilkinson

Funding:	Wellcome Trust Intermediate Fellowship Number: WT098316
Ethics Committee:	University of Cape Town Human Research Ethics Committee (protocol still to be submitted for approval)

Date: 1 December 2014
Version: 4.0

Aims of the Study:

1) To determine which of the following factors (potentially amenable to therapeutic intervention) are associated with mortality among hospitalized patients with HIV-associated tuberculosis:
    a. Sepsis syndrome caused by *M. tuberculosis*
    b. Intestinal translocation of bacterial products and viable bacteria
    c. Cytomegalovirus viraemia
    d. Immune activation-induced apoptosis and anti-inflammatory signaling
    e. Paradoxical TB-associated immune reconstitution inflammatory syndrome

2) To determine the proportion of hospitalized HIV-TB patients having subtherapeutic concentrations of antitubercular drugs during the critical early period of TB treatment.

Background:

Tuberculosis is the commonest cause of death amongst HIV-infected people in Africa [1-3]. In 2007, there were an estimated 378,000 deaths due to HIV-TB in Africa and 94,000 in South Africa [4]. Young adults are worst affected [5]. This occurs despite antiretroviral therapy (ART) scale-up and many die after having started TB treatment. In recent RCTs [6-8], starting ART ~2 weeks into TB treatment in patients with low CD4 counts reduced mortality and AIDS progression. Yet 7-18% of patients who started ART at 2 weeks in these trials died. In routine care, particularly among hospitalized patients, mortality is higher. There are few detailed studies that define pathophysiology and mechanisms of death. Our proposed study will address why patients die despite TB treatment, early ART and co-trimoxazole prophylaxis. Our intention is to define interventions to reduce mortality. Based on study findings, novel strategies such as targeted gram-negative bacterial prophylaxis, treatment of CMV viraemia, immunomodulatory therapy, improved TB drug treatment strategies or corticosteroid prophylaxis to prevent TB-IRIS could be tested in RCTs.

In-hospital mortality of HIV-TB patients is particularly high. Among 1006 South African patients diagnosed with TB (80% HIV-infected), 19% died in hospital and 6% died after discharge [9]. At our own hospital, TB was the cause of 56% of HIV-related deaths [10]. Amongst hospitalised HIV-infected TB suspects in Uganda, 2-month mortality was 32% [11]. The final cause of death is poorly defined. A number of mechanisms are possible: major organ dysfunction and septic shock due to disseminated TB [12]; bacterial sepsis; other opportunistic infections; pulmonary embolism; neurological complications of TB; and drug-resistant TB. African HIV post-mortem studies have identified bacterial infections and CMV organ disease as common co-morbidities [13, 14]. The role of intestinal translocation as portal of entry for bacterial infections, similar to the process that occurs in cirrhosis [15, 16] has not been explored in patients with advanced HIV-TB. Severe depletion of intestinal CD4 T cells results in

translocation of lipopolysaccharide (LPS) and other bacterial products contributing to immune activation and disease progression during chronic HIV infection [17, 18]. We hypothesise that in patients with advanced HIV and disseminated TB, intestinal immunity is more profoundly impaired (as both diseases affect the intestine and result in nutritional deficiency and intestinal hypoperfusion may complicate disseminated TB). High levels of LPS and bacterial DNA translocation resulting in immune activation may contribute to a sepsis syndrome associated with disseminated TB. Translocation of viable intestinal bacteria may result in fatal sepsis.

Work which has led up to this project:
We have published 2 studies that investigated the causes of clinical deterioration during TB treatment in hospitalized patients and outpatients respectively. In both, the most frequent causes for deterioration were AIDS-related opportunistic infections, TB-IRIS and bacterial infections (Appendix 1) [19, 20]. Nine patients in the hospital study were found to have extended-spectrum beta-lactamase producing Enterobacteriaceae infections (7 had a fatal outcome).

We also conducted a cohort study of inpatients with HIV-TB (n=113) during the first 3 months of ART to assess incidence and features of TB-IRIS, drug toxicities and opportunistic/bacterial infections at Brooklyn Chest TB Hospital. Nosocomial infections occurred in 16%: Enterobacteriaceae were isolated in 12/23.

A 2-year review of blood cultures at GF Jooste Hospital demonstrated 217 gram-negative (89 *E. coli*; 49 *Klebsiella* species) and 15 *Enterococcus* species (Appendix 2). These findings support the hypothesis of translocation of intestinal organisms. This is likely an underestimate of blood stream infections as insufficient blood cultures are routinely performed.

We supervised Sathyavani Subbarao at Madwaleni Hospital (Eastern Cape) who assessed predictors of 2-month mortality among admitted HIV-TB patients (Appendix 3). When data for the first 79 patients were analysed it was found that 35% died a median of 8 days (IQR= 6-20) after admission. Elevated lactate was independently associated with reduced survival (adjusted OR = 0.41 (95% CI = 0.24-0.71)).

For paradoxical TB-IRIS, we performed a randomized placebo-controlled trial of prednisone treatment. A 4-week course of prednisone reduced morbidity and symptoms [21]. We have also published studies on the immunopathogenesis and diagnosis of TB-IRIS [22-25]. In the diagnostic study, among 100 patients presenting with suspected TB-IRIS, 7 were diagnosed with alternative opportunistic diseases and 9 with rifampicin-resistance [23].

__Rationale and methods for each research question__:

1a) Is fatal HIV-TB associated with a sepsis syndrome caused by *M. tuberculosis* itself resulting in major organ dysfunction and septic shock?
The hypothesis is that disseminated TB results in an inflammatory cascade in the blood causing sepsis syndrome, severe sepsis and septic shock [26-29]. Patients with mycobacteraemia, particularly with higher levels, would be more at risk for these complications and mortality. This hypothesis will be tested by correlating clinical and laboratory markers of sepsis with quantitative markers of *M.tuberculosis* load. All patients with positive bacterial cultures from blood or other sterile site or clinically diagnosed bacterial infection (e.g. pneumonia) will be excluded from this analysis.

__Investigations__: Patients will be assessed for clinical features of sepsis syndrome, severe sepsis and septic shock, defined using standard criteria [28]. Echocardiography will assess left ventricular function (dysfunction may occur with sepsis syndrome and contribute to shock). Laboratory investigations to assess sepsis syndrome: C-reactive protein (CRP) [30], procalcitonin [31, 32], full blood count, D-dimer [33], venous blood gas, lactate (Appendix 3), liver/renal function and glucose.

Investigations to assess *M.tuberculosis* load quantitatively:

i) Mycobacterial blood culture (Days to positivity);
ii) Quantitative urinary LAM Clearview TB ELISA;
iii) Semi-quantitative urinary LAM Alere Lateral Flow Assay (Intensity measurement of bands serves as semi-quantitation);
iv) Sputum Xpert MTB/RIF (Cycle threshold as relative quantitation) and culture (Days to positivity).

The individual quantitative measures of *M.tuberculosis* load will be correlated with markers of sepsis syndrome to assess association (univariate analysis and multiple linear regression), and comparisons will be made for all variables between fatal cases and survivors.

1b) Is fatal HIV-TB associated with translocation of bacteria and their products from the intestine into blood?
Under this aim, the first hypothesis is that in patients with severe HIV-TB, intestinal translocation of bacterial products (LPS and bacterial DNA) will be higher than in control HIV-infected patients with similar CD4 counts who do not have TB. The second hypothesis is that mortality will be associated with higher levels of bacterial product translocation. Thirdly we hypothesise that translocation of viable intestinal bacteria in some patients results in life-threatening deterioration and death.

To test the first 2 hypotheses the following will be performed:

i) Plasma LPS concentration using Chromogenic Limulus Amebocyte Lysate test [17];
ii) Plasma soluble CD14 (secreted by macrophages/monocytes in response to LPS) by ELISA [17, 34];

iii) Plasma intestinal fatty acid binding protein (I-FABP; a marker of small intestine mucosal damage) [35-38] by ELISA;

iv) Whole blood bacterial DNA concentrations will be measured using Abbott PLEX-ID platform [39-41]. Specimens will be shipped to Abbott Laboratory (Carlsbad, CA, US). Steps are: DNA extraction (using reagents prepared to minimise bacterial contamination), PCR amplification of bacterial DNA using broad-range, unbiased primers targeted at conserved regions of bacterial genomes, then electrospray ionization/mass spectroscopy. Amplicons are identified by ESI/MS based on recognisable patterns of base composition for different bacterial species. Quantification is performed by comparison with an internal calibrant. Similar approaches were used to study translocation in liver disease [15, 16] and inflammatory bowel disease [42].

We will perform LPS, sCD14 and I-FABP in all 660 participants and compare with the non-TB control group (n=90). For the PLEX-ID we will perform 270 PLEX-ID assays in a nested case-control study (90 fatal cases, 90 survivors and 90 non-TB controls). For all experiments comparisons will be made between fatal cases and survivors using multiple linear regression to adjust for covariates (eg. CD4 count, antibiotic treatment).

To test the third hypothesis bacterial blood cultures will be performed on all patients on admission and at any clinical deterioration with fever or features of sepsis syndrome [28]. Sputum bacterial cultures will be performed on all with radiographic pulmonary infiltrates. Bacterial cultures will also be performed on other appropriate specimens (eg. urine, pus). The contribution of bacterial infection in general to clinical deterioration and deaths will thereby be ascertained. We will make assessment of the likely source of bacteraemia (eg. respiratory or urinary tract, intestinal) based on the organism and cultures from other clinical sites.

1c) Is fatal HIV-TB associated with cytomegalovirus (CMV) viraemia?

CMV viraemia has been associated with mortality in HIV-infected patients [43-47]. Amongst HIV-infected South African miners (mainly WHO stage 1 and 2) 10.9% had detectable CMV viraemia and this was associated with 3-fold higher mortality [48]. Undiagnosed organ disease (eg. pneumonitis) may contribute to mortality as suggested by post-mortem studies [13, 14]. Viraemia may also contribute to HIV disease progression [48]. It is also possible that CMV viraemia interferes with immune responses to other pathogens: CMV viral replication may directly damage infected immune cells through lysis and expansion of CMV-specific CD8 T-lymphocytes in peripheral blood may reduce the pool of naïve T-cells [49]. To address this hypothesis we will perform CMV viral load determination using quantitative PCR (Roche Cobas AmpliPrep/Cobas Taqman platform at Groote Schuur Hospital Virology Laboratory). We will also examine for features of CMV disease by:

i) pan-optic fundoscopy;

ii) gastro-intestinal tract biopsies in patients who have endoscopy and at post-mortem.

If a strong independent association between CMV viraemia and mortality is found we will propose and seek funding for an RCT of ganciclovir pre-emptive therapy or aciclovir prophylaxis, similar to practice in transplant recipients [50].

1d) Is fatal HIV-TB associated with immune activation-induced apoptosis and worsening immunosuppression?

Bacterial sepsis is characterized by an early phase of pro-inflammatory "cytokine storm". It is now recognized that this is followed by a phase characterized by monocyte deactivation, impaired neutrophil function, lymphocyte dysfunction and apoptosis [51, 52], increased production of IL-10 and TGF-β [53] and increased proportion of circulating T-regulatory cells [54]. This "immunosuppressive phase" results in a heightened risk for secondary bacterial infections [51, 55]. We will explore whether severe HIV-TB, through similar mechanisms, results in an immunosuppressive state (prior to ART) that exacerbates HIV-related immunosuppression and may predispose to bacterial infections. Specifically, we will explore whether severe HIV-TB and mortality are associated with:

i) Immune activation-induced apoptosis;
ii) Increased programmed death-1 (PD-1) signaling;
iii) Anti-inflammatory cytokine profile.

Experiments will be performed on samples from:

- 290 patients in the cohort sampled consecutively provided FACS can be performed the same day (estimated mortality of 15% will result in 44 fatal cases and 246 survivors);
- Non-TB control group (n=30).

i) Immune-activation induced apoptosis

We hypothesise that severe HIV-TB induces apoptosis of immune cells and that this is associated with mortality. PBMC will be separated and same day FACS analysis for surface markers of cell activation (CD38, HLA-DR) and a marker of apoptosis (Annexin-V) on CD4 and CD8 T-lymphocytes and monocytes will be performed on a BD LSR Fortessa instrument at the IDM. Data will be analysed using FlowJo software (TreeStar Inc, FlowJo Africa scheme).

ii) PD-1 signaling pathway

Ligation of PD-1 (found on T and B cells and innate immune cells) by its ligands PD-L1 and PD-L2, results in cell cycle arrest, impaired proliferation and function, apoptosis and up-regulation of anti-inflammatory signals [56, 57]. In septic shock patients, PD-1, PD-L1 and PD-L2 expression on monocytes and CD4 T-lymphocytes was significantly elevated. Furthermore, PD-L1 expression on monocytes was higher in those patients with septic shock that died, and higher PD-1 expression on monocytes was associated with secondary nosocomial infections [56]. Other studies demonstrated upregulation of PD-1 on T cells and PD-L1 on monocytes in septic shock patients [58] and that PD-1 upregulation leads to macrophage dysfunction in sepsis [59]. Murine models have demonstrated improved survival from sepsis when the PD-1/PD-L1 pathway is blocked [58, 59]. Using FACS on fresh PBMC (as above), we will assess

expression of PD-1 and its ligands on T cells (CD4 and CD8), monocytes and neutrophils.

iii) Anti-inflammatory cytokines and ratio to pro-inflammatory cytokines

Lower TNF-α responses to LPS and heat-killed *M.tuberculosis* were associated with poor outcome in Malawi [60]. In Zambia fatal TB was associated with reduced LPS-induced TNF-α, IL-6 and IL-8 [61]. In sepsis higher IL-10 concentrations have been linked to mortality [62, 63].

We hypothesise that fatal HIV-TB is associated with higher concentrations of anti-inflammatory cytokines and higher ratio of anti-inflammatory to pro-inflammatory cytokines. Cytokine concentrations will be measured using Luminex (Bio-Plex platform) and ELISA in stored plasma. A 12-plex Luminex assay will be run: IL1-beta, IL-1 receptor antagonist, IL-4, IL-5, IL-6, IL-8, IL-10, IL-12, IL-13, IFN-γ, TNF-α, GM-CSF. TGF-β will be assayed using ELISA. Ratios of anti-inflammatory to pro-inflammatory cytokines will be derived (eg. IL-10/IFN-γ).

The findings from these 3 experiments will be analysed comparing survivors vs fatal cases vs non-TB controls. Data will also be correlated with measures of *M.tuberculosis* load, LPS, HIV viral load and CMV viral load in univariate analysis and multiple linear regression models.

1e) Paradoxical TB-associated immune reconstitution inflammatory syndrome (TB-IRIS)

Paradoxical TB-IRIS occurs in 8-43% of HIV-TB patients starting ART and manifests with recurrence of inflammatory TB features [22]. Reported case fatality rate is 3.2% [64]. Shorter interval between TB treatment and ART is an important risk factor with recent clinical trials showing 2-5-fold increased risk of TB-IRIS in those who start ART around 2 weeks [6-8]. With earlier ART in operational settings, TB-IRIS incidence and attributable mortality will likely increase. We will assess for TB-IRIS using our consensus case definition (Appendix 4) [22] that has been independently validated [65-68]. In TB-IRIS suspects, we will investigate to exclude differential diagnoses [23]. We will thus ascertain TB-IRIS cases and determine mortality attributable to the condition, augmented by post-mortem findings.

2) Is severe HIV-TB associated with subtherapeutic concentrations of antitubercular drugs during the critical early period of treatment?

PK studies done by Prof Helen McIlleron have demonstrated lower rifampicin and ethambutol concentrations in HIV-infected patients [69]. In Botswana HIV-infected patients with CD4 < 200 cells/μl had significantly lower pyrazinamide and rifampicin Cmax than those with CD4 > 200 cells/μl, and lower pyrazinamide Cmax was independently associated with treatment failure or death [70]. Among 16 HIV-TB patients with CD4 < 100 cells/μl rifampicin exposure was invariably low, especially during the first 2 weeks [71]. TB PK studies have almost exclusively involved healthy volunteers or patients with pulmonary TB in outpatient settings. Our concern is that among acutely ill hospitalized patients with advanced HIV and disseminated TB reduced intestinal

motility and malabsorption is significant. Some patients require TB drug administration through naso-gastric tubes; crushing of tablets or adsorption to plastic might compromise drug delivery [72]. The adequacy of drug concentrations has not been adequately assessed in these patients. Given the high mycobacterial load in disseminated TB, early bactericidal drug activity may be critical for favorable outcomes. We will take samples from 2 groups enrolled in the study: a randomly selected group (n=40) and a group receiving TB treatment via naso-gastric route (n=40). We will recruit 2 groups of controls: HIV-infected (n=40) and HIV-uninfected (n=40) patients starting TB treatment in an ambulatory setting at Ubuntu HIV-TB clinic in Khayelitsha.

We will perform sparse sampling pre-dose and at 1, 2.5, 4, 6 and 8 hours after TB drug dosing at day 3 of TB treatment and store plasma at -80°C. Isoniazid, rifampicin and pyrazinamide concentrations will be measured using LC-MS. The PK measurements will be performed on stored, batched samples and thus results of these assays will not be available in real-time and thus will not influence patients management. The UCT Clinical Pharmacology Laboratory has performed many TB drug PK studies [69, 73-75]. Population PK modelling will be used to predict important PK parameters (Cmax and AUC). Findings would inform further research to optimize initial dosing or route of administration (eg. intravenous) of TB medication in severe HIV-TB.

Methodology:

Study design:
Prospective cohort study of 660 HIV-TB inpatients.

Sample size calculations:
The primary outcome was dichotomised (survivors vs fatal outcome). The calculations have been performed for major experiments relating to urinary LAM concentration, plasma LPS concentration and proportion with detectable CMV viraemia. Cumulative mortality of 15% has been factored: lower than reported (9). We assume in a prospective study with ART initiation at 2-8 weeks mortality will be lower than reported from routine settings.

Urinary LAM concentration (ng/ml). Mean level of urinary LAM of 1.62 ng/ml in survivors has been estimated based on HIV-TB outpatient data obtained from author of [76], Stephen Lawn:

Survivors Urinary LAM (Mean; SD)	Fatal outcome Urinary LAM (Mean; SD)	Fold change	Alpha	Beta	Sample size
1.62; 3.2	2.43; 3.2	1.5	0.05	0.2	962
1.62; 3.2	3.24; 3.2	2	0.05	0.2	242
1.62; 3.2	4.05; 3.2	2.5	0.05	0.2	108

Plasma LPS concentration (pg/ml). Mean level of LPS in survivors of 128 pg/ml estimated, based on [17]:

Survivors Plasma LPS (Mean; SD)	Fatal outcome Plasma LPS (Mean; SD)	Difference %	Alpha	Beta	Sample size
128, 56	160, 56	25	0.05	0.2	160
128, 56	154, 56	20	0.05	0.2	286
128, 56	147, 56	15	0.05	0.2	536

Detectable CMV viral load by PCR (> 150 copies/ml). Based on [48] it was estimated that CMV viraemia would be detectable in 10-20% survivors (higher than in [48] because participants will have more advanced HIV):

Survivors % CMV viral load detectable	Fatal outcome % CMV viral load detectable	Alpha	Beta	Sample size
20%	40%	0.05	0.2	334
20%	30%	0.05	0.2	1178
10%	30%	0.05	0.2	246
10%	20%	0.05	0.2	786

660 patients will be enrolled. Assuming 5% loss to follow up, 630 patients will complete the study. We will exclude from major analyses patients diagnosed with rifampicin resistance (estimated 5% [77], n=30), leaving 600 for analysis. This will allow us to detect statistical significance for a two-fold difference in urinary LAM, a 15% difference in plasma LPS and a difference in the percentage of patients with detectable CMV viraemia of 20% in survivors and 40% in those with fatal outcome (or 10% and 30% respectively).

Sample size for FACS experiments (44 fatal cases vs 246 survivors) was based on Annexin-V experiments, allowing us to detect a significant difference in the proportion of CD4 T-lymphocytes that are Annexin-V+ of 5% in survivors and 20% in those who die (alpha=0.05, beta=0.2).

Characteristics of the study population:

Number of participants:
660 participants with HIV-TB co-infection.

Inclusion criteria:
1. Microbiological diagnosis of TB, or clinical diagnosis that fulfills WHO criteria for smear-negative or extrapulmonary TB [78] or suspected to have active TB based on clinical presentation
2. 18 years or older
3. Informed consent from patient (unless drowsy/confused – see below)
4. HIV seropositive
5. CD4 count ≤ 350cells/µl

Exclusion criteria:
1. HIV seronegative or testing declined
2. Pregnant
3. 3 or more doses of TB treatment received during the admission or has been on TB treatment within one month of admission

TB case definitions:
TB will be investigated with 2 sputa for Xpert MTB/RIF and TB culture, Myco/F Lytic blood culture, urinary lipoarabinomannan (LAM) and samples from appropriate extrapulmonary sites for TB culture. Chest radiographs will be performed in all and abdominal ultrasound at clinician discretion. We will stratify analyses according to:
1) Confirmed TB: Microbiologically proven TB (by culture or Xpert MTB/RIF, or post-mortem findings of AFB+ or culture+) (predicted to be > 70% based on previous study at our hospital [79])
2) Probable TB: TB not confirmed, but urinary LAM+ or fulfill WHO criteria [78] unless an alternate diagnosis is made during follow-up or post-mortem.

Vulnerability:
Patients who are admitted with HIV-TB co-infection (or suspected co-infection) may be confused or drowsy at time of hospital admission due to neurological TB or a general medical condition. Effort will be made to include patients who are confused or drowsy, yet eligible for the study, in order to make results generalizable to these patients.
In previous discussion with UCT HREC (in relation to a similar observational cohort study that was performed at Madwaleni Hospital, HREC REF 136/2011) regarding consent for drowsy or confused patients we were made aware that relatives or next of kin may not legally provide consent for participation in research. However, in situations where the exclusion of such patients would compromise the generalizability of the results and the risks involved in participation are minimal, such patients may be enrolled with the permission of the Ethics Committee in each case. In this study it is critically important that we obtain data on the most severely ill patients to avoid our results being biased. We thus seek Ethics Committee permission to enroll such patients (confused or

drowsy patients who fulfill inclusion criteria but are unable to provide consent to the study). If family members are present we will explain our actions to the closest adult relatives. We will not ask them to sign informed consent, but if they request us for any reason to not enroll the patient, we will respect their wishes. When such patients regain the capacity to provide informed consent, the study will be discussed with them. If they choose to participate then they will sign informed consent. If they decline to participate then their study data and sample will be destroyed. If the patient does not regain the cognitive ability to consent, specific permission will be sought from the UCT HREC to use that patient's data and sample for the study in each case. We seek permission to use this approach for enrolment for the main study and for the PK sub study (specifically for the group who are receiving medication via a nasogastric tube in the PK sub study). We will not enroll any patients who are unable to consent for genetic testing or as control patients.

HIV testing:
Patients who are known to be HIV infected will not be re-tested prior to enrolment. HIV viral load will be performed as part of study procedures and will serve as a confirmatory test. Patients who do not know their HIV status will be offered an HCT test prior to enrolment. This HIV test will be performed according to standard hospital procedures and done prior to any study procedures, usually by hospital counselors (it is standard practice to offer HCT to all patients diagnosed with TB at KDH). Patients who decline HIV testing will not be enrolled in the study. Patients who are drowsy or confused and have unknown HIV status will not be enrolled.

No children, adolescents or pregnant women will be enrolled in this study.

Study site:
Khayelitsha District Hospital is a public sector district level hospital. Antenatal HIV seroprevalence is 30% and TB notification rate is 1,600 / 100,000 in the catchment area. Patients with HIV-TB too ill to be initially managed as outpatients are admitted. Approximately 50 eligible patients are admitted each month. Once stabilised, patients are referred for inpatient treatment at a TB hospital or outpatient TB treatment at local clinic. Formal provincial permission will be obtained from the PGWC research committee once ethics permission has been obtained.

Recruitment and enrolment:
Participants will be enrolled as soon as possible after presentation to hospital and followed for 12 weeks. The team will aim to enroll patients prior to the first dose of TB treatment, but up to two doses of TB treatment will be allowed. Patients who have had three doses or more of TB treatment will not be enrolled. Eligible patients will be identified by members of the study team (study nurse, counselor and medical officer) on the post-intake ward round in the emergency room every morning during the week (Monday to Friday) and also in the medical wards. Posters with contact details of the study team members will be placed in the hospital (submitted to HREC for approval).

20 patients will be enrolled monthly. When potential enrollees exceed capacity on a day then recruitment will be randomised (using simple random allocation calculator) to avoid bias. This method of selecting participants to enroll will be described in an SOP. The study counselor or study nurse will administer the informed consent document with support from the study medical officer. The informed consent document makes it clear that patients will receive the same standard of care should they decline to take part in the study and that collection and testing of genetic material is optional. In addition, they will be specifically requested to consent to storage of samples for future research and informed that this is optional. After informed consent is obtained, the study team will follow up patients for 12 weeks initially as an inpatient then as an outpatient.

Research procedures and follow up (see table below):
Cumulative mortality at 12 weeks will be ascertained. Paradoxical TB-IRIS and other causes for clinical deterioration will be ascertained during the 12 weeks. Participants not on ART will initiate ART 2-8 weeks into TB treatment (2 weeks if CD4 ≤ 50/μl).

Study assessments will be at 3 days (while in hospital) and subsequently every 3 days while patient remains hospitalized. The study team will review patients at any re-hospitalisation. The team will make telephonic contact at 4 weeks after enrolment and will do a clinical review of all participants at 12 weeks after enrolment. Additional study visits will be arranged in order to determine the cause of and assist with management of clinical deterioration if necessary. We will investigate for TB drug resistance and additional diagnoses (including serum cryptococcal antigen testing [80]). Patients with drug resistance or other diagnoses will be included in the study, with these findings recorded.

At discharge from hospital patients will be referred to their local TB clinic and for continuation of their TB treatment and their local ART clinic for initiation or continuation of ART. A referral letter will be written by the KDH staff or the research staff. If at any time point there is concern that patients are not on appropriate medication, the team will contact their local clinic. They will be asked to bring their medications and TB card to the clinical review visit.

Post-mortem examinations:
Relatives will be approached for permission for post-mortem examinations. If unwilling to have a full post-mortem performed, permission will be sought to undertake post-mortem needle biopsies of lung, liver and spleen and bone marrow – an approach that yields important diagnostic information in HIV-infected patients [14, 81]. Tests performed on tissue samples will include: histological stains; Gram, fungal and ZN stains; immunohistochemical staining (CMV); and bacterial, TB and fungal cultures. This study will be performed in collaboration with Prof Helen Wainwright (Anatomical Pathology, UCT).

Study Outline of Activities:

	D0	D3	Wk2	Wk4	Wk8	Wk12	Deterioration
Informed consent	x						
Clinical Assessment	x	x				x	x
Samples Collected	x	* PK					[#]x
[Δ]ART initiation CD4 ≤ 50			x				
[Δ]ART initiation CD4 >50			x				

D: Day
Wk: Week
ART: Antiretroviral Therapy
PK: PK substudy in a subset of participants
* PK samples will be stored and run in batches retrospectively.
[#] Samples taken at time of deterioration will not be research samples, but standard of care samples appropriate to investigate the patients' condition at that time point.
[Δ] If patient is not on ART at time of recruitment

Detailed Study Procedures:

Study procedure	Time Point	Detail	Volume	Standard of care or research
Clinical Assessment	All time points	Examine for features of sepsis syndrome and TB IRIS	NA	Standard of care
	D0	* Pan ophthalmic fundoscopy for features of CMV retinitis	NA	Research
Echocardiography	D0 & Deterioration	* Assess left ventricular function	NA	Research
Blood tests	D0 & Deterioration	**Chemistry:** * Glucose	2 ml	Standard of care
		* Lactate		Research
		* D-dimer	5 ml	Research
		*Pro-calcitonin	1 ml	Research
		* Venous blood gas	1 ml	Research
		* Liver function tests	5 ml	Standard of care
		* Renal function tests		Standard of care
		* CRP		Standard of care
		**Haematology:** * Full blood count and differential	4 ml	Standard of care
		**Plasma:** LPS concentration	2 ml	Research
		Soluble CD14	1 ml	Research
		Intestinal fatty acid binding protein	1 ml	Research
		**Microbiology:** * Bacterial blood culture	8 ml	Standard of care
		* Mycobacterial blood culture	5 ml	Research
		* Serum cryptococcal antigen lateral flow assay	0.5 ml	Research
		**Virology:** CMV viral load	2 ml	Research
		* HIV viral load	2 ml	Research
		**Immunology:** PBMC	10 ml	Research
		* CD 4 count	2 ml	Standard of care
		**Genetic:** DNA	2 ml	Research
		Luminex and ELISA for cytokines	Plasma from PBMC sample	Research
		PLEX-ID assay for bacterial DNA	3ml	Research
Urine	D0 & Deterioration	Urinary LAM assays	20 ml	Research
Sputum	D0 & Deterioration	* Gene Xpert MTB/RIF and TB culture	NA	Standard of Care
Chest Xray	D0 & Deterioration	* Evaluate for any abnormality	NA	Standard of care
Sputum bacterial cultures	D0 & Deterioration	* All patients with infiltrate on CXR	NA	Research
Bacterial cultures on other samples	D0 & Deterioration	* Pus, urine or other samples as appropriate	NA	Standard of care
Post mortem examination	Death	If family consents to post mortem	NA	Research

D: Day
Wk: Week
NA: Not applicable
* These tests will be performed in real time and results will be made available to managing doctors as soon as the lab result is available.
Note: If any of these samples have been taken by hospital staff within 2 days of enrollment they will not be repeated but the results from the hospital sample will be used.

Consent will specifically be taken from patients for storage of remaining plasma, serum, urine and PBMC for potential future substudies (such substudies would require specific HREC permission). In addition, we will store a sample of DNA for potential future studies of genetic polymorphisms associated with outcome in HIV-TB. Again consent will be specifically obtained for this with a separate Genetic ICF. It will be made clear to participants that sample storage is optional.

In addition, PK samples will be performed on a subset of participants as described above on day 3 of TB treatment with 4ml of blood drawn at each of the timepoints (pre-dose, 1, 2.5, 4, 6 and 8 hours after taking TB medication). Samples for PK studies will be stored and run in batches and will thus not be performed in real time. Results will not be available to attending medical teams.

Control cohort of non-TB HIV-infected patients:
We will enroll a cohort of 90 hospitalised ART-naïve HIV-infected patients with CD4 counts ≤ 350/μl. We will exclude TB (2 sputum cultures and urinary LAM all negative). These will be patients admitted with other HIV-related conditions (eg. cryptococcal meningitis, pneumocystis pneumonia, Kaposi's sarcoma).

Data collection methods:
Clinical data will be abstracted onto hardcopy data capture forms for each specified study visit and unscheduled visits at clinical deterioration. A death form will be used to capture information regarding the circumstances of the death from hospital and laboratory records or relatives' verbal report, in order that a cause of death can be assigned.

Data safety and monitoring:
Each participant will have a unique identifying study number and this will be recorded together with the patient's name and contact details in a study log. The study log (and all the patients' data capture forms in a study folder for each participant) will be kept in a locked cabinet in a limited-access office at Khayelitsha District Hospital. All our clinical and laboratory data entry forms and samples will only be labelled with the patient's study ID number. The anonymised patient study folders will be transported to the IDM for the purposes of computer database data entry and then returned to the hospital. Laboratory results will be obtained from the NHLS, Abbott Laboratory and our own laboratory at the IDM. There is an electronic data management system at the IDM for entering laboratory results that will be utilised.

It is important to note that the patients name and details will be entered on the NHLS lab forms and data system as this facilitates linking results to patients for the medical teams looking after them at the hospital. NHLS result sheets with the patient's name on them will however not be transported off-site to IDM for data entry but will be transcribed onto anonymised data forms before being transported to IDM for data entry. All samples sent to IDM labs or Abbott will be anonymised.

All clinical data and laboratory results will be entered into an Access database on a dedicated hard drive at the UCT Institute of Infectious Diseases and Molecular Medicine (IDM). The computer is password protected. There are 3 trained data capturers working within the Clinical Infectious Diseases Research Initiative (established through a Wellcome Trust Strategic Award) at the IDM who will perform this. Access allows the construction of multiple databases (eg. for clinical data and for different laboratory assays) to link to one another through the study ID number of the participant. Back-up will occur daily. All data on the database is anonymised.

Data quality control and assurance measures will be put in place. These will include having all clinical data capture forms being checked and signed off by one other member of the study team. Data entered on the study database will be entered by one data capturer, but will be checked by a member of the study team or by another data capturer. Any errors will be recorded and discussed at the monthly study meeting. Upon completion of the study we will ensure that 3 copies of the raw data are backed-up for long term storage: one on a UCT server, one on a remote server and one on an external hard-drive that the applicant will keep secure. These data capture, entry, management and access systems have been developed in the course of many TB-HIV and other HIV studies that we have undertaken to date.

Data analysis:
TB suspects who do not have active TB (after full clinical investigation has been completed and all results obtained) will not be included in the analysis of primary endpoints. Their data may be used in secondary analyses or as control subjects. Univariate analysis will compare those who survive 12 weeks to those with fatal outcome. Bonferroni correction for multiple comparisons will be performed where appropriate. Multivariable logistic regression modeling will be approached based on pre-specified hypotheses, articulated where possible through directed acyclic graphs. Demographic variables, CD4 count, viral load, antibiotic treatment will be explored as potential confounders in addition to hypothesis specific variables. Comparison with non-TB control patients will be performed for several of the experiments as described. Data will be analyzed using de-identified study numbers.

Description of risks and benefits:
Potential risks and discomforts: This is an observational trial and there are no study specific interventions. Collection of study samples by means of phlebotomy carries minimal risk of bruising, infection at the collection site and bleeding after phlebotomy. Phlebotomy may be uncomfortable or painful during the procedure, but this resolves as soon as the procedure has been completed. The total volume of blood drawn is ~ 55ml. This amount of blood drawn would not cause complications unless patients are severely anaemic.
Risk classification: The study as a whole carries minimal risk for participants and harm due to study participation is not anticipated.
Minimizing risk: Only trained personnel will perform phlebotomy. Patients who have symptomatic anaemia (Hb<8g/dl with symptoms attributable to anaemia) will not have more than 30ml of blood drawn. An SOP will be developed to guide

what key assays will be performed in such patients in order to maximize the investigations and assays that can be performed on this 30ml (those that impact on patient management will be prioritized).

Potential benefits: This study will not directly benefit participants. The study results will contribute to management and hopefully improve survival of patients with similar problems in the future. Many of the study investigations will be performed in real time (these are indicated in the table above) and results will be made available to the medical teams responsible. Participants will have an experienced and dedicated study team who will advise on their medical treatment during the first three months of tuberculosis treatment and during ART initiation (if applicable). People who choose not to participate in the study will still receive standard of care treatment for tuberculosis and HIV.

Informed consent process:

Process: A study team member will approach patients in the emergency room or medical wards. Every effort will be made to make the space as confidential as possible, i.e. draw curtains around the bed, look for a side room if patient is well enough to mobilize. The informed consent process will be delegated to the study nurse and the study counselor. The study medical officer will support the process. All study personnel will have Good Clinical Practice training and will follow these principles closely. The Principal Investigator will provide training of the study-specific informed consent process and monitor that informed consent is being taken that fulfills GCP requirements throughout the study. The informed consent document will be discussed with the patient in detail in their language of choice (Xhosa or English). The ICF's will be available in both languages. The patient will be given time to consider the study and time to discuss with their relatives if required. The patient will be given time to ask questions. At each follow up visit there will be opportunity to revisit any aspects of the study the participant may wish to.

Capacity to consent: If a potential participant appears confused, the study medical officer will assess the patient. If the patient is unable to give informed consent, and the participant is eligible for the study (i.e. known HIV infected and to be started on TB treatment during admission), the participant will be enrolled on the study. This will be discussed with relatives as outlined above. Once the patient regains cognitive ability to consent, the study will be discussed with them. If the patient is agreeable, they will sign the informed consent document. If they do not want to take part in the study, their samples and data will be destroyed. If the participant does not regain the ability to consent, specific permission will be sought from the UCT HREC to use that patient's data and sample for the study.

Comprehension of information: Patient's understanding will be tested with standard questions prior to signing the informed consent documents.

No information regarding the study will be withheld from participants.

The Informed Consent Form, Genetic Consent Form, Controls Consent Form and PK Study Consent Form will be translated into Xhosa once approved by the ethics committee.

Privacy and confidentiality:

Each participant will have a unique identifying study number and this will be recorded together with the patient's name and contact details in a study log. The study log will be kept in a locked cabinet in a limited-access office at Khayelitsha District Hospital. Only the study team will have access to these records. All clinical and laboratory data forms and samples will only be labelled with the patient's study ID number (apart from NHLS samples which will have the patient's name on them as outlined above). All data on the electronic database will be anonymised.

Reimbursement for participation:
Participants will be reimbursed for traveling expenses when they attend outpatient clinic visits (R100). Patients will be given a small food parcel on enrollment as a token of appreciation (total cost < R50). This will not be used as an incentive to persuade patients to be enrolled on the study, but rather be given after enrolment to say thank you for enrolling on the study. We are aware of the fact that we will be collecting a number of additional samples from these patients, which will take some time and effort from their side, especially at time of enrolment, and thus think a token of appreciation that also serves to provide additional nutrition is appropriate. Patients who also participate in the PK-studies on day 3 of TB treatment will be reimbursed transport money if they are attending an outpatient clinic as a control patient. This will be a minimum of R100 but may be more, depending on when recruitment for PK control patients start. Inpatients who are recruited in hospital will not receive a transport fee but will receive a toiletry hamper which contains basic toiletries that can be used during the patients hospital stay. If a patient is too ill to receive and use these items themselves every effort will be made to give this to an adult relative who spends time with this patient in hospital or the nurse who cares for this person at the time of the study.

Emergency care and insurance for research-related injuries:
The UCT Ethics Committee no faults insurance will cover all injuries incurred as a result of participation in the study. Emergency care will be provided by the clinical service at Khayelitsha District Hospital.

What happens at the end of the study?
Participants will be transferred to their appropriate primary care clinic with a referral letter at the end of 12 weeks. Results will be disseminated in local and international meetings and peer reviewed journals.

Study timelines:

**Months 1-6**	**Months 7-42**	**Month 43-49**	**Months 50-60**
Prepare SOPs, administration, recruit staff, ethical and PGWC permission			

	Recruitment and follow-up of clinical cohort and controls		
	Laboratory experiments		
			Analysis, interpretation and write-up of results

References:

1. Ansari NA, Kombe AH, Kenyon TA, Hone NM, Tappero JW, Nyirenda ST*, et al.* Pathology and causes of death in a group of 128 predominantly HIV-positive patients in Botswana, 1997-1998. *Int J Tuberc Lung Dis* 2002,**6**:55-63.
2. Lucas SB, De Cock KM, Hounnou A, Peacock C, Diomande M, Honde M*, et al.* Contribution of tuberculosis to slim disease in Africa. *BMJ* 1994,**308**:1531-1533.
3. Rana FS, Hawken MP, Mwachari C, Bhatt SM, Abdullah F, Ng'ang'a LW*, et al.* Autopsy study of HIV-1-positive and HIV-1-negative adult medical patients in Nairobi, Kenya. *J Acquir Immune Defic Syndr* 2000,**24**:23-29.
4. *Global Tuberculosis Control 2009: Epidemiology, strategy, financing*: World Health Organisation, Geneva; 2009.
5. Abdool Karim SS, Churchyard GJ, Abdool Karim Q, Lawn SD. HIV infection and tuberculosis in South Africa: an urgent need to escalate the public health response. *Lancet* 2009,**374**:921-933.
6. Blanc FX, Sok T, Laureillard D, Borand L, Rekacewicz C, Nerrienet E*, et al.* Earlier versus later start of antiretroviral therapy in HIV-infected adults with tuberculosis. *The New England journal of medicine* 2011,**365**:1471-1481.
7. Havlir DV, Kendall MA, Ive P, Kumwenda J, Swindells S, Qasba SS*, et al.* Timing of antiretroviral therapy for HIV-1 infection and tuberculosis. *The New England journal of medicine* 2011,**365**:1482-1491.
8. Abdool Karim SS, Naidoo K, Grobler A, Padayatchi N, Baxter C, Gray AL*, et al.* Integration of antiretroviral therapy with tuberculosis treatment. *The New England journal of medicine* 2011,**365**:1492-1501.
9. Edginton ME, Wong ML, Phofa R, Mahlaba D, Hodkinson HJ. Tuberculosis at Chris Hani Baragwanath Hospital: numbers of patients diagnosed and outcomes of referrals to district clinics. *Int J Tuberc Lung Dis* 2005,**9**:398-402.
10. Behroozi F, **Meintjes G**, Crede T, Burch V, Rebe K. . Causes of mortality in relation to HIV status and antiretroviral access at a referral hospital in Cape Town, South Africa. In: *5th International AIDS Society Conference on HIV Pathogenesis, Treatment and Prevention*. Cape Town, South Africa; 2009.

11. Kyeyune R, den Boon S, Cattamanchi A, Davis JL, Worodria W, Yoo SD*, et al.* Causes of early mortality in HIV-infected TB suspects in an East African referral hospital. *J Acquir Immune Defic Syndr*,**55**:446-450.
12. Vadillo M, Corbella X, Carratala J. AIDS presenting as septic shock caused by Mycobacterium tuberculosis. *Scand J Infect Dis* 1994,**26**:105-106.
13. Martinson NA, Karstaedt A, Venter WD, Omar T, King P, Mbengo T*, et al.* Causes of death in hospitalized adults with a premortem diagnosis of tuberculosis: an autopsy study. *AIDS* 2007,**21**:2043-2050.
14. Wong EB, Omar T, Setlhako G, Osih R, Murdoch D, Martinson N, Feldman C, Bangsberg D, Venter WDF. Causes of death in ART-treated adults: a post-mortem study from Johannesburg. Poster WEPE0154. In: *XVIII International AIDS Conference*. Vienna; 2010.
15. Frances R, Benlloch S, Zapater P, Gonzalez JM, Lozano B, Munoz C*, et al.* A sequential study of serum bacterial DNA in patients with advanced cirrhosis and ascites. *Hepatology* 2004,**39**:484-491.
16. Zapater P, Frances R, Gonzalez-Navajas JM, de la Hoz MA, Moreu R, Pascual S*, et al.* Serum and ascitic fluid bacterial DNA: a new independent prognostic factor in noninfected patients with cirrhosis. *Hepatology* 2008,**48**:1924-1931.
17. Brenchley JM, Price DA, Schacker TW, Asher TE, Silvestri G, Rao S*, et al.* Microbial translocation is a cause of systemic immune activation in chronic HIV infection. *Nat Med* 2006,**12**:1365-1371.
18. Kamat A, Ancuta P, Blumberg RS, Gabuzda D. Serological markers for inflammatory bowel disease in AIDS patients with evidence of microbial translocation. *PLoS One*,**5**:e15533.
19. Pepper DJ, Marais S, Wilkinson RJ, Bhaijee F, Maartens G, McIlleron H*, et al.* Clinical deterioration during antituberculosis treatment in Africa: incidence, causes and risk factors. *BMC Infect Dis*,**10**:83.
20. Pepper DJ, Rebe K, Morroni C, Wilkinson RJ, Meintjes G. Clinical deterioration during antitubercular treatment at a district hospital in South Africa: the importance of drug resistance and AIDS defining illnesses. *PLoS One* 2009,**4**:e4520.
21. Meintjes G, Wilkinson RJ, Morroni C, Pepper DJ, Rebe K, Rangaka MX*, et al.* Randomized placebo-controlled trial of prednisone for paradoxical tuberculosis-associated immune reconstitution inflammatory syndrome. *AIDS* 2010,**24**:2381-2390.
22. Meintjes G, Lawn SD, Scano F, Maartens G, French MA, Worodria W*, et al.* Tuberculosis-associated immune reconstitution inflammatory syndrome: case definitions for use in resource-limited settings. *The Lancet infectious diseases* 2008,**8**:516-523.
23. Meintjes G, Rangaka MX, Maartens G, Rebe K, Morroni C, Pepper DJ*, et al.* Novel relationship between tuberculosis immune reconstitution inflammatory syndrome and antitubercular drug resistance. *Clinical infectious diseases : an official publication of the Infectious Diseases Society of America* 2009,**48**:667-676.
24. Meintjes G, Wilkinson KA, Rangaka MX, Skolimowska K, van Veen K, Abrahams M*, et al.* Type 1 helper T cells and FoxP3-positive T cells in HIV-tuberculosis-associated immune reconstitution inflammatory syndrome. *Am J Respir Crit Care Med* 2008,**178**:1083-1089.

25. Tadokera R, Meintjes G, Skolimowska KH, Wilkinson KA, Matthews K, Seldon R, *et al.* Hypercytokinaemia accompanies HIV-tuberculosis immune reconstitution inflammatory syndrome. *The European respiratory journal : official journal of the European Society for Clinical Respiratory Physiology* 2011,**37**:1248-1259.
26. Cohen J. The immunopathogenesis of sepsis. *Nature* 2002,**420**:885-891.
27. Lever A, Mackenzie I. Sepsis: definition, epidemiology, and diagnosis. *BMJ* 2007,**335**:879-883.
28. Levy MM, Fink MP, Marshall JC, Abraham E, Angus D, Cook D*, et al.* 2001 SCCM/ESICM/ACCP/ATS/SIS International Sepsis Definitions Conference. *Intensive Care Med* 2003,**29**:530-538.
29. Russell JA. Management of sepsis. *N Engl J Med* 2006,**355**:1699-1713.
30. Lawn SD, Wiktor S, Coulibaly D, Ackah AN, Lal RB. Serum C-reactive protein and detection of tuberculosis in persons co-infected with the human immunodeficiency virus. *Trans R Soc Trop Med Hyg* 2001,**95**:41-42.
31. Nyamande K, Lalloo UG. Serum procalcitonin distinguishes CAP due to bacteria, Mycobacterium tuberculosis and PJP. *The international journal of tuberculosis and lung disease : the official journal of the International Union against Tuberculosis and Lung Disease* 2006,**10**:510-515.
32. Schleicher GK, Herbert V, Brink A, Martin S, Maraj R, Galpin JS*, et al.* Procalcitonin and C-reactive protein levels in HIV-positive subjects with tuberculosis and pneumonia. *The European respiratory journal : official journal of the European Society for Clinical Respiratory Physiology* 2005,**25**:688-692.
33. Maartens G, Willcox PA, Benatar SR. Miliary tuberculosis: rapid diagnosis, hematologic abnormalities, and outcome in 109 treated adults. *Am J Med* 1990,**89**:291-296.
34. Sandler NG, Wand H, Roque A, Law M, Nason MC, Nixon DE*, et al.* Plasma levels of soluble CD14 independently predict mortality in HIV infection. *The Journal of infectious diseases* 2011,**203**:780-790.
35. Derikx JP, Poeze M, van Bijnen AA, Buurman WA, Heineman E. Evidence for intestinal and liver epithelial cell injury in the early phase of sepsis. *Shock* 2007,**28**:544-548.
36. Pan L, Wang X, Li W, Li N, Li J. The intestinal fatty acid binding protein diagnosing gut dysfunction in acute pancreatitis: a pilot study. *Pancreas*,**39**:633-638.
37. Pelsers MM, Hermens WT, Glatz JF. Fatty acid-binding proteins as plasma markers of tissue injury. *Clin Chim Acta* 2005,**352**:15-35.
38. Wiercinska-Drapalo A, Jaroszewicz J, Siwak E, Pogorzelska J, Prokopowicz D. Intestinal fatty acid binding protein (I-FABP) as a possible biomarker of ileitis in patients with ulcerative colitis. *Regul Pept* 2008,**147**:25-28.
39. Ecker DJ, Sampath R, Li H, Massire C, Matthews HE, Toleno D*, et al.* New technology for rapid molecular diagnosis of bloodstream infections. *Expert Rev Mol Diagn*,**10**:399-415.
40. Leggieri N, Rida A, Francois P, Schrenzel J. Molecular diagnosis of bloodstream infections: planning to (physically) reach the bedside. *Curr Opin Infect Dis*,**23**:311-319.

41. Wolk DM, Blyn LB, Hall TA, Sampath R, Ranken R, Ivy C*, et al.* Pathogen profiling: rapid molecular characterization of Staphylococcus aureus by PCR/electrospray ionization-mass spectrometry and correlation with phenotype. *J Clin Microbiol* 2009,**47**:3129-3137.
42. Gutierrez A, Holler E, Zapater P, Sempere L, Jover R, Perez-Mateo M*, et al.* Antimicrobial peptide response to blood translocation of bacterial DNA in Crohn's disease is affected by NOD2/CARD15 genotype. *Inflamm Bowel Dis*.
43. Chevret S, Scieux C, Garrait V, Dahel L, Morinet F, Modai J*, et al.* Usefulness of the cytomegalovirus (CMV) antigenemia assay for predicting the occurrence of CMV disease and death in patients with AIDS. *Clin Infect Dis* 1999,**28**:758-763.
44. Deayton JR, Prof Sabin CA, Johnson MA, Emery VC, Wilson P, Griffiths PD. Importance of cytomegalovirus viraemia in risk of disease progression and death in HIV-infected patients receiving highly active antiretroviral therapy. *Lancet* 2004,**363**:2116-2121.
45. Griffiths PD. CMV as a cofactor enhancing progression of AIDS. *J Clin Virol* 2006,**35**:489-492.
46. Podzamczer D, Ferrer E, Garcia A, Ramon JM, Niubo J, Santin M*, et al.* pp65 antigenemia as a marker of future CMV disease and mortality in HIV-infected patients. *Scand J Infect Dis* 1997,**29**:223-227.
47. Wohl DA, Zeng D, Stewart P, Glomb N, Alcorn T, Jones S*, et al.* Cytomegalovirus viremia, mortality, and end-organ disease among patients with AIDS receiving potent antiretroviral therapies. *J Acquir Immune Defic Syndr* 2005,**38**:538-544.
48. Fielding K, Koba A, Grant AD, Charalambous S, Day J, Spak C*, et al.* Cytomegalovirus Viremia as a Risk Factor for Mortality Prior to Antiretroviral Therapy among HIV-Infected Gold Miners in South Africa. *PLoS One* 2011,**6**:e25571.
49. Moss P. The emerging role of cytomegalovirus in driving immune senescence: a novel therapeutic opportunity for improving health in the elderly. *Current opinion in immunology* 2010,**22**:529-534.
50. Kalil AC, Levitsky J, Lyden E, Stoner J, Freifeld AG. Meta-analysis: the efficacy of strategies to prevent organ disease by cytomegalovirus in solid organ transplant recipients. *Annals of internal medicine* 2005,**143**:870-880.
51. Goyert SM, Silver J. Editorial: PD-1, a new target for sepsis treatment: better late than never. *Journal of leukocyte biology* 2010,**88**:225-226.
52. Wang TS, Deng JC. Molecular and cellular aspects of sepsis-induced immunosuppression. *Journal of molecular medicine* 2008,**86**:495-506.
53. Cavaillon JM, Adib-Conquy M. Bench-to-bedside review: endotoxin tolerance as a model of leukocyte reprogramming in sepsis. *Critical care* 2006,**10**:233.
54. Venet F, Chung CS, Kherouf H, Geeraert A, Malcus C, Poitevin F*, et al.* Increased circulating regulatory T cells (CD4(+)CD25 (+)CD127 (-)) contribute to lymphocyte anergy in septic shock patients. *Intensive care medicine* 2009,**35**:678-686.
55. Hotchkiss RS, Opal S. Immunotherapy for sepsis--a new approach against an ancient foe. *The New England journal of medicine* 2010,**363**:87-89.

56. Guignant C, Lepape A, Huang X, Kherouf H, Denis L, Poitevin F*, et al.* Programmed death-1 levels correlate with increased mortality, nosocomial infection and immune dysfunctions in septic shock patients. *Critical care* 2011,**15**:R99.
57. Said EA, Dupuy FP, Trautmann L, Zhang Y, Shi Y, El-Far M*, et al.* Programmed death-1-induced interleukin-10 production by monocytes impairs CD4+ T cell activation during HIV infection. *Nature medicine* 2010,**16**:452-459.
58. Zhang Y, Li J, Lou J, Zhou Y, Bo L, Zhu J*, et al.* Upregulation of programmed death-1 on T cells and programmed death ligand-1 on monocytes in septic shock patients. *Critical care* 2011,**15**:R70.
59. Huang X, Venet F, Wang YL, Lepape A, Yuan Z, Chen Y*, et al.* PD-1 expression by macrophages plays a pathologic role in altering microbial clearance and the innate inflammatory response to sepsis. *Proceedings of the National Academy of Sciences of the United States of America* 2009,**106**:6303-6308.
60. Waitt CJ, Peter KBN, White SA, Kampmann B, Kumwenda J, Heyderman RS*, et al.* Early deaths during tuberculosis treatment are associated with depressed innate responses, bacterial infection, and tuberculosis progression. *The Journal of infectious diseases* 2011,**204**:358-362.
61. Friedland JS, Hartley JC, Hartley CG, Shattock RJ, Griffin GE. Inhibition of ex vivo proinflammatory cytokine secretion in fatal Mycobacterium tuberculosis infection. *Clinical and experimental immunology* 1995,**100**:233-238.
62. Monneret G, Venet F, Pachot A, Lepape A. Monitoring immune dysfunctions in the septic patient: a new skin for the old ceremony. *Molecular medicine* 2008,**14**:64-78.
63. Gogos CA, Drosou E, Bassaris HP, Skoutelis A. Pro- versus anti-inflammatory cytokine profile in patients with severe sepsis: a marker for prognosis and future therapeutic options. *The Journal of infectious diseases* 2000,**181**:176-180.
64. Muller M, Wandel S, Colebunders R, Attia S, Furrer H, Egger M. Immune reconstitution inflammatory syndrome in patients starting antiretroviral therapy for HIV infection: a systematic review and meta-analysis. *The Lancet infectious diseases* 2010,**10**:251-261.
65. Eshun-Wilson I, Havers F, Nachega JB, Prozesky HW, Taljaard JJ, Zeier MD*, et al.* Evaluation of paradoxical TB-associated IRIS with the use of standardized case definitions for resource-limited settings. *Journal of the International Association of Physicians in AIDS Care* 2010,**9**:104-108.
66. Haddow LJ, Moosa MY, Easterbrook PJ. Validation of a published case definition for tuberculosis-associated immune reconstitution inflammatory syndrome. *Aids* 2010,**24**:103-108.
67. Manosuthi W, Van Tieu H, Mankatitham W, Lueangniyomkul A, Ananworanich J, Avihingsanon A*, et al.* Clinical case definition and manifestations of paradoxical tuberculosis-associated immune reconstitution inflammatory syndrome. *Aids* 2009,**23**:2467-2471.
68. Sharma SK, Dhooria S, Barwad P, Kadhiravan T, Ranjan S, Miglani S*, et al.* A study of TB-associated immune reconstitution inflammatory syndrome

using the consensus case-definition. *The Indian journal of medical research* 2010,**131**:804-808.

69. McIlleron H, Wash P, Burger A, Norman J, Folb PI, Smith P. Determinants of rifampin, isoniazid, pyrazinamide, and ethambutol pharmacokinetics in a cohort of tuberculosis patients. *Antimicrobial agents and chemotherapy* 2006,**50**:1170-1177.
70. Chideya S, Winston CA, Peloquin CA, Bradford WZ, Hopewell PC, Wells CD*, et al.* Isoniazid, rifampin, ethambutol, and pyrazinamide pharmacokinetics and treatment outcomes among a predominantly HIV-infected cohort of adults with tuberculosis from Botswana. *Clinical infectious diseases : an official publication of the Infectious Diseases Society of America* 2009,**48**:1685-1694.
71. Saleri N, Dembele SM, Villani P, Carvalho AC, Cusato M, Bonkoungou V*, et al.* Systemic exposure to rifampicin in patients with tuberculosis and advanced HIV disease during highly active antiretroviral therapy in Burkina Faso. *The Journal of antimicrobial chemotherapy* 2011.
72. Nahata MC, Morosco RS, Hipple TF. Stability of rifampin in two suspensions at room temperature. *Journal of clinical pharmacy and therapeutics* 1994,**19**:263-265.
73. McIlleron H, Willemse M, Schaaf HS, Smith PJ, Donald PR. Pyrazinamide plasma concentrations in young children with tuberculosis. *The Pediatric infectious disease journal* 2011,**30**:262-265.
74. McIlleron H, Willemse M, Werely CJ, Hussey GD, Schaaf HS, Smith PJ*, et al.* Isoniazid plasma concentrations in a cohort of South African children with tuberculosis: implications for international pediatric dosing guidelines. *Clinical infectious diseases : an official publication of the Infectious Diseases Society of America* 2009,**48**:1547-1553.
75. Schaaf HS, Willemse M, Cilliers K, Labadarios D, Maritz JS, Hussey GD*, et al.* Rifampin pharmacokinetics in children, with and without human immunodeficiency virus infection, hospitalized for the management of severe forms of tuberculosis. *BMC medicine* 2009,**7**:19.
76. Lawn SD, Edwards DJ, Kranzer K, Vogt M, Bekker LG, Wood R. Urine lipoarabinomannan assay for tuberculosis screening before antiretroviral therapy diagnostic yield and association with immune reconstitution disease. *AIDS* 2009,**23**:1875-1880.
77. Cox HS, McDermid C, Azevedo V, Muller O, Coetzee D, Simpson J*, et al.* Epidemic levels of drug resistant tuberculosis (MDR and XDR-TB) in a high HIV prevalence setting in Khayelitsha, South Africa. *PloS one* 2010,**5**:e13901.
78. World Health Organisation. Improving the diagnosis and treatment of smear-negative pulmonary and extrapulmonary tuberculosis among adults and adolescents. Recommendations for HIV-prevalent resource-constrained settings. In. Geneva: WHO Press; 2006.
79. Wilson D, Nachega J, Morroni C, Chaisson R, Maartens G. Diagnosing smear-negative tuberculosis using case definitions and treatment response in HIV-infected adults. *The international journal of tuberculosis and lung disease : the official journal of the International Union against Tuberculosis and Lung Disease* 2006,**10**:31-38.

80. Jarvis JN, Harrison TS, Govender N, Lawn SD, Longley N, Bicanic T*, et al.* Routine cryptococcal antigen screening for HIV-infected patients with low CD4+ T-lymphocyte counts--time to implement in South Africa? *South African medical journal = Suid-Afrikaanse tydskrif vir geneeskunde* 2011,**101**:232-234.
81. Cohen T, Murray M, Wallengren K, Alvarez GG, Samuel EY, Wilson D. The prevalence and drug sensitivity of tuberculosis among patients dying in hospital in KwaZulu-Natal, South Africa: a postmortem study. *PLoS Med*,**7**:e1000296.

Appendices:

Appendix 1:

Causes for deterioration on TB treatment among patients presenting to a referral hospital (GF Jooste Hospital) in Cape Town. Causes for deterioration among 352 patients who deteriorated clinically while on TB treatment and required assessment at the referral hospital (GF Jooste Hospital) over a 3-month period in 2007 [20].

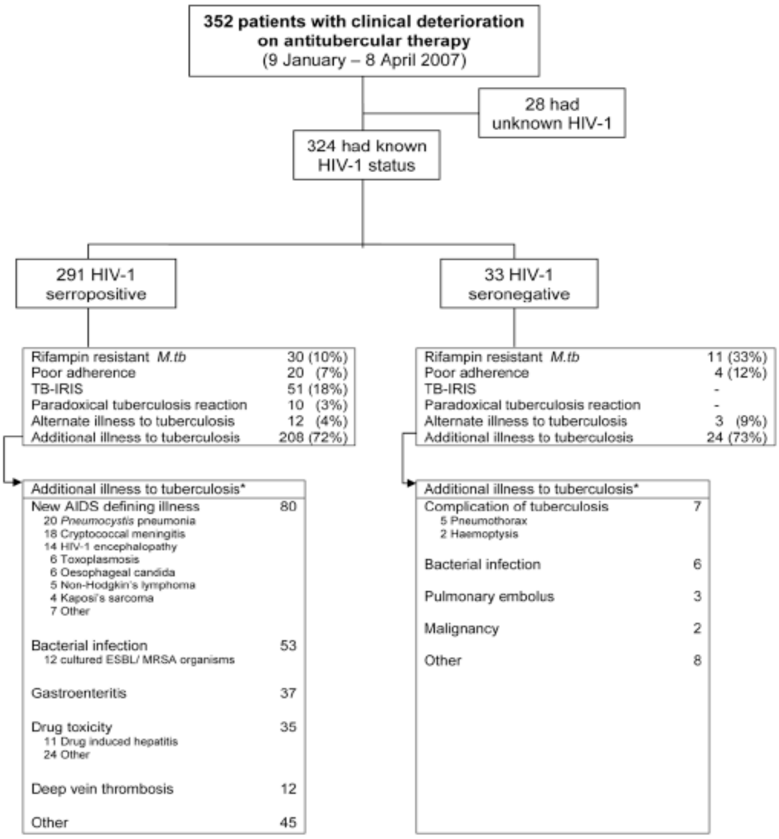

Reference: Pepper DJ, Rebe K, Morroni C, Wilkinson RJ, Meintjes G. 2009. Clinical deterioration during antitubercular treatment at a district hospital in South Africa: the importance of drug resistance and AIDS defining illnesses. *PLoS One* 4:e4520.

Appendix 2:
Positive blood culture results from GF Jooste Hospital over a 2-year period
June 2008 – June 2010. Isolates that were non-pathogenic organisms have been excluded.

**Genus or species**	**Number of isolates**
Acinetobacter species	9
Alpha-haemolytic *streptococci*	16
Beta-haemolytic *streptococci*	24
Citrobacter species	1
Enterobacter species	14
Enterococcus species	15
E.coli	89
Haemophilus species	9
Klebsiella species	49
Morganella species	4
Neisseria meningitidis	2
Proteus species	12
Pseudomonas species	8
Non-typhi Salmonella species	26
Salmonella typhi	2
Serratia species	1
Shigella species	4
Staphylococcus aureus	57
Streptococcus pneumoniae	60
TOTAL	402

Appendix 3:
Factors associated with 2-month mortality in univariate analysis in cohort study of hospitalized HIV-TB patients at Madwaleni Hospital, Eastern Cape, South Africa (preliminary analysis of first participants to complete follow-up, n=79). Consecutive HIV-infected admitted patients started on TB treatment were enrolled in the study.

Variable*	Survivors N=51	Died N=28	p-value for comparison**
Respiratory rate	30 (23-34)	30 (20-35)	0.90
Heart rate	124 (110-140)	117 (103-138)	0.24
Oxygen saturation	96 (95-99)	96.5 (90-98)	0.48
Glucose (mmol/l)	5 (4.1-6.1)	4.5 (2.4-6.5)	0.17
Weight (kg)	49 (43-56)	48 (40-56)	0.68
C-reactive protein (mg/l)	121 (56-209)	207 (117-305)	**0.01**
Creatinine (μmol/l)	63 (51-104)	150 (77-227)	**0.0003**
Lactate (mmol/l)	3.2 (2.2-4.1)	5.4 (3.7-6.1)	**<0.0001**
Haemoglobin (g/dl)	9.0 (6.9-10.2)	7.6 (5.7-9.5)	0.15
CD4 count (cells/μl)	76 (25-190)	31 (14-136)	0.08

* Median and IQR are shown (admission values)
** Wilcoxon rank-sum test

Lactate measurements on admission comparing those who died versus those that survived 2 months:

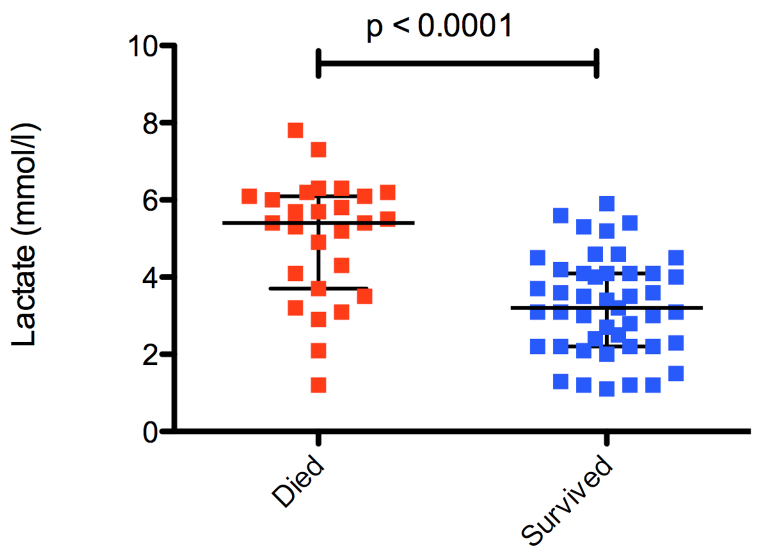

Appendix 4:
International Network for the Study of HIV-associated IRIS (INSHI) consensus case definition for paradoxical TB-IRIS that will be used:

Table I. Case definition for paradoxical TB-IRIS[3]

There are 3 components to this case definition:

A. Antecedent requirements

Both of the 2 following requirements must be met:

1. Diagnosis of TB: the TB diagnosis was made before starting ART and this should fulfil WHO criteria for diagnosis of smear-positive PTB, smear-negative PTB or extrapulmonary TB
2. Initial response to TB treatment: the patient's condition should have stabilised or improved on appropriate TB treatment before ART initiation – e.g. cessation of night sweats, fevers, cough, weight loss. (Note: this does not apply to patients starting ART within 2 weeks of starting TB treatment since insufficient time may have elapsed for a clinical response to be reported.)

Clinical criteria

The onset of TB-IRIS manifestations should be within 3 months of ART initiation, re-initiation, or regimen change because of treatment failure.
Of the following, at least 1 major criterion or 2 minor clinical criteria are required:

Major criteria

1. New or enlarging lymph nodes, cold abscesses or other focal tissue involvement – e.g. tuberculous arthritis
2. New or worsening radiological features of TB (found by chest X-ray, abdominal USS, CT or MRI)
3. New or worsening central nervous system TB (meningitis or focal neurological deficit – e.g. caused by tuberculoma)
4. New or worsening serositis (pleural effusion, ascites, or pericardial effusion)

Minor criteria

1. New or worsening constitutional symptoms such as fever, night sweats, or weight loss
2. New or worsening respiratory symptoms such as cough, dyspnoea, or stridor
3. New or worsening abdominal pain accompanied by peritonitis, hepatomegaly, splenomegaly, or abdominal adenopathy

C. Alternative explanations for clinical deterioration must be excluded if possible

1. Failure of TB treatment due to TB drug resistance
2. Poor adherence to TB treatment
3. Another opportunistic infection or neoplasm (it is particularly important to exclude an alternative diagnosis in patients with smear-negative PTB and extrapulmonary TB where the initial TB diagnosis has not been microbiologically confirmed)
4. Drug toxicity or reaction

Reference: Meintjes G, Lawn SD, Scano F, Maartens G, French MA, Worodria W, Elliott JH, Murdoch D, Wilkinson RJ, Seyler C, John L, van der Loeff MS, Reiss P, Lynen L, Janoff EN, Gilks C, Colebunders R; International Network for the Study of HIV-associated IRIS.et al. 2008. Tuberculosis-associated immune reconstitution inflammatory syndrome: case definitions for use in resource-limited settings. *Lancet Infectious Diseases* 8:516-523.
