# Supplementary material for: Clinical, microbiologic, and immunologic determinants of mortality in hospitalized patients with HIV-associated tuberculosis: A prospective cohort study
Source: PLoS Med. 2019 Jul 5;16(7):e1002840. doi: 10.1371/journal.pmed.1002840 (PMC6611568; doi:10.1371/journal.pmed.1002840)
Supplement: S1 STROBE checklist — (DOCX) [file pmed.1002840.s013.docx]

STROBE Statement—checklist of items that should be included in reports of observational studies

|  | Item No | Recommendation |
| --- | --- | --- |
| **Title and abstract** | 1 | (*a*) Indicate the study’s design with a commonly used term in the title or the abstract  **Our study design is indicated in the title and abstract (prospective cohort study)** |
|  |  | (*b*) Provide in the abstract an informative and balanced summary of what was done and what was found  **We provide details of what was done and our findings in the abstract: Methods and Findings** |
| Introduction | | |
| Background/rationale | 2 | Explain the scientific background and rationale for the investigation being reported  **We provide background and rationale for the investigation: Section: Introduction** |
| Objectives | 3 | State specific objectives, including any prespecified hypotheses  **We state the objectives and hypothesis in the Introduction section, last paragraph** |
| Methods | | |
| Study design | 4 | Present key elements of study design early in the paper  **We present the study design in the first paragraph of the Methods section: Study design and Setting.** |
| Setting | 5 | Describe the setting, locations, and relevant dates, including periods of recruitment, exposure, follow-up, and data collection  **We present the setting, location, recruitment dates, follow-up time, methods of follow-up and data collection in the Methods section: Study design and setting, Participants, Data collection and definitions.** |
| Participants | 6 | (*a*) *Cohort study*—Give the eligibility criteria, and the sources and methods of selection of participants. Describe methods of follow-up  **We provide these details in Methods section: Participants.**  *Case-control study*—Give the eligibility criteria, and the sources and methods of case ascertainment and control selection. Give the rationale for the choice of cases and controls  *Cross-sectional study*—Give the eligibility criteria, and the sources and methods of selection of participants |
|  |  | (*b*) *Cohort study*—For matched studies, give matching criteria and number of exposed and unexposed  **Not applicable**  *Case-control study*—For matched studies, give matching criteria and the number of controls per case |
| Variables | 7 | Clearly define all outcomes, exposures, predictors, potential confounders, and effect modifiers. Give diagnostic criteria, if applicable  **We provide these details in the Methods section: Data collection and definitions.** |
| Data sources/ measurement | 8* | For each variable of interest, give sources of data and details of methods of assessment (measurement). Describe comparability of assessment methods if there is more than one group  **We provide these details in the Methods section: Data collection and definitions and Laboratory assays.** |
| Bias | 9 | Describe any efforts to address potential sources of bias  **We used a random selection method if there were more potentially eligible patients than we had capacity to enrol (Methods section: Participants). We randomly selected samples to test immunological markers (Methods section: Laboratory Assays).** |
| Study size | 10 | Explain how the study size was arrived at  **The sample size calculations were performed for major experiments in the original protocol. We include this in the Methods section: Statistical analysis, first paragraph** |
| Quantitative variables | 11 | Explain how quantitative variables were handled in the analyses. If applicable, describe which groupings were chosen and why  **We describe this in the Methods section: Statistical analysis.** |
| Statistical methods | 12 | (*a*) Describe all statistical methods, including those used to control for confounding  **We describe this in the Methods section: Statistical analysis.** |
|  |  | (*b*) Describe any methods used to examine subgroups and interactions  **We describe this in the Methods: Statistical analysis section** |
|  |  | (*c*) Explain how missing data were addressed  **We describe this in the Methods section: Laboratory assays and Statistical analysis. Details about missing data is included in the footnotes of tables where relevant.** |
|  |  | (*d*) *Cohort study*—If applicable, explain how loss to follow-up was addressed  **We describe this in the Methods section: Statistical analysis, first paragraph**  *Case-control study*—If applicable, explain how matching of cases and controls was addressed  *Cross-sectional study*—If applicable, describe analytical methods taking account of sampling strategy |
|  |  | (*e*) Describe any sensitivity analyses  **We describe this in the Methods section: Statistical analysis (second last paragraph) and Results section: Variance of host soluble inflammatory mediators.** |

Continued on next page

| Results | | |
| --- | --- | --- |
| Participants | 13* | (a) Report numbers of individuals at each stage of study—eg numbers potentially eligible, examined for eligibility, confirmed eligible, included in the study, completing follow-up, and analysed  **We report this in the Results section: Participant characteristics and mortality and Figure 1.** |
|  |  | (b) Give reasons for non-participation at each stage  **We report this in Figure 1.** |
|  |  | (c) Consider use of a flow diagram  **See Figure 1.** |
| Descriptive data | 14* | (a) Give characteristics of study participants (eg demographic, clinical, social) and information on exposures and potential confounders  **We provide this in the Results section: Participant characteristics and mortality, and Table 2** |
|  |  | (b) Indicate number of participants with missing data for each variable of interest  **We provide this in Results section: Participant characteristics and mortality and Table 2.** |
|  |  | (c) *Cohort study*—Summarise follow-up time (eg, average and total amount)  **All patients were followed up for 12 weeks. 12-week outcome was ascertained, and we provide this data in the Methods section: Participants (last paragraph) and Data collection and definitions (first paragraph).** |
| Outcome data | 15* | *Cohort study*—Report numbers of outcome events or summary measures over time  **We provide this in the Results section: Participant characteristics and mortality and Table 2.** |
|  |  | *Case-control study—*Report numbers in each exposure category, or summary measures of exposure |
|  |  | *Cross-sectional study—*Report numbers of outcome events or summary measures |
| Main results | 16 | (*a*) Give unadjusted estimates and, if applicable, confounder-adjusted estimates and their precision (eg, 95% confidence interval). Make clear which confounders were adjusted for and why they were included  **We report raw and adjusted p-values and 95% confidence intervals where appropriate throughout the Results section and in the legends of relevant figures.** |
|  |  | (*b*) Report category boundaries when continuous variables were categorized  **We report the category boundaries in the Cox proportional hazards models where we categorized age and HIV viral load: Results section: Variance of host soluble inflammatory mediators and Figure 5.** |
|  |  | (*c*) If relevant, consider translating estimates of relative risk into absolute risk for a meaningful time period  **Not applicable.** |
| Other analyses | 17 | Report other analyses done—eg analyses of subgroups and interactions, and sensitivity analyses  **We describe all analyses in detail in the Methods section: Statistical analysis and in the figure legends. We also describe a sensitivity analysis performed on request of a reviewer in the Methods section: Statistical analyses (second last paragraph) and Results: Variance of host soluble inflammatory mediators.** |
| Discussion | | |
| Key results | 18 | Summarise key results with reference to study objectives  **We summarised our findings in the first paragraph of the Discussion section.** |
| Limitations | 19 | Discuss limitations of the study, taking into account sources of potential bias or imprecision. Discuss both direction and magnitude of any potential bias  **We discuss the limitations of the study in the Discussion section, paragraph 11.** |
| Interpretation | 20 | Give a cautious overall interpretation of results considering objectives, limitations, multiplicity of analyses, results from similar studies, and other relevant evidence  **We compare our finding with other studies in the field and give cautious interpretation of findings throughout the Discussion section.** |
| Generalisability | 21 | Discuss the generalisability (external validity) of the study results  **We discuss generalizability of these results in the Discussion section under strengths, paragraph 12.** |
| Other information | | |
| Funding | 22 | Give the source of funding and the role of the funders for the present study and, if applicable, for the original study on which the present article is based  **We provide details of funding and the role of the funders separately as requested by this journal. We also include the original study protocol. This paper constitutes the main study results.** |

*Give information separately for cases and controls in case-control studies and, if applicable, for exposed and unexposed groups in cohort and cross-sectional studies.

**Note:** An Explanation and Elaboration article discusses each checklist item and gives methodological background and published examples of transparent reporting. The STROBE checklist is best used in conjunction with this article (freely available on the Web sites of PLoS Medicine at http://www.plosmedicine.org/, Annals of Internal Medicine at http://www.annals.org/, and Epidemiology at http://www.epidem.com/). Information on the STROBE Initiative is available at www.strobe-statement.org.
